# Supplementary material for: Hierarchical effects of historical and environmental factors on lizard assemblages in the upper Madeira River, Brazilian Amazonia
Source: PLoS One. 2020 Jun 2;15(6):e0233881. doi: 10.1371/journal.pone.0233881 (PMC7266318; doi:10.1371/journal.pone.0233881)
Supplement: S1 Data — Predictors used as independent variables in the ecological models to test lizard assemblage structuring in the upper Madeira River, Brazilian Amazonia. (PDF) [file pone.0233881.s001.pdf]

S 1. Protocols

NUMBER OF TREES. –The density sampling method used transects that varied in size depending on the size of the plant class being surveyed, as follows: (Transect 1) plants with a diameter at breast height (DBH) ≥ 1 cm were sampled in 1m-wide band to the left side of the centerline, for the entire length of the sample plot; (Transect 2) plants with DBH ≥ 10 cm were sampled in a 20m wide band, with 10 m on either side of the plot center line. On the left, this band included Transects 1 and 3; (Transect 3) plants with DBH ≥ 30 cm were sampled in a range of 40 m wide, 20 m being on each side of the center line of the plot. On the left, this range overlapped with Transects 1 and 2, where all plants with DBH greater than or equal to 1 or 10 cm were measured. On the right side, this band includes Transect 2. More details of the plant data collection component are available at: http://ppbio.inpa.gov.br/sites/default/files/Estrutura_vegetacao.pdf. For statistical analyzes the total density of plants (sum values from Transects 1, 2 and 3 combined) per plot was used.

SOIL NUTRIENT COMPOSITION. –Soil pH was obtained from the effective H+ ion concentration, determined with a combined electrode directly immersed in a soil solution diluted with distilled water at a 1:2.5ml ratio. Calcium, Magnesium and exchangeable Aluminum were extracted with KCl 1M. Exchangeable Al+3 was titrated with NaOH 0.025M using bromothymol blue as an indicator. Ca+2 and Mg+2 levels were determined by atomic absorption spectrophotometry (AAS). Potassium and soil micronutrients (iron, zinc and manganese) was extracted with Mehlich I2 extraction solution (double-acid solution), consisting of a mixture of HCl 0.05M + H2SO4 0.0125M. The mL extract ratio was 1:10. K, Fe, Zn and Mn were determined by AAS. Available phosphorus was determined with a colorimetric spectrophotometer, using 3% ammonium molybdate and

ascorbic acid. Using these values, formula was applied to the sum of bases, following the methods of Quesada et al. (2010), which allowed soil fertility in each plot to be inferred. For statistical analyzes the value for the sum of bases in each plot was used.

SOIL SAND AND CLAY CONTENT. –Soil samples were collected at six points on each plot (0, 50, 100, 150, 200 e 250 m along the plot length), at depths of 0 and 5 cm, once surface leaves had been removed. Samples were collected with a 5.5 cm diameter manual auger, stored in plastic bags and subsequently dried at room temperature and cleaned with tweezers, removing all pieces of leaf, root and charcoal. The material was processed and screened with a 2mm mesh sieve, then separated from other soil impurities to yield Fine Air-Dried Earth – FADE. Particle size analysis was made with composite samples (mixing sub-samples from soil obtained at 0, 50,100, 150, 200 and 250 m) at INPA's Soil and Plant Science Laboratory. Particle size was estimated from an aliquot of 10 grams of soil by adding the chemical dispersant sodium pyrophosphate to separate soil particles. Organic matter was oxidized by heating with hydrogen peroxide. The proportion of clay was determined by dry weight of 20 ml of soil suspension. The coarse fraction (fine and coarse sand) were separated by sieving, dried in an oven (105 ° C for 24 hours) and weighed to obtain the respective percentages, following the PPBio methodology (http://ppbio.inpa.gov.br/knb/metacat). The average percentage of clay in the soil of each plot was used to represent particle size and this was then used in statistical analyzes.

ELEVATION−A professional surveyor measured the elevation above sea level at the starting point of the plot. This was done because, to minimize variation in vegetation, soil type and drainage, each plot follows the local contour line and, consequently, variation in

elevation is minimal along its length. The value of the elevation of each plot was used for statistical analysis.

Literature Cited

Quesada CA, Lloyd J, Schwarz M, Patiño S, Baker RT, Czimczik C et al. Variations in chemical and physical properties of Amazon forest soils in relation to their genesis. Biogeosciences. 2010; 7: 1515–1541. doi: 10.5194/bg-7-1515-2010.
