# Supplementary figures and images for: Hierarchical effects of historical and environmental factors on lizard assemblages in the upper Madeira River, Brazilian Amazonia
Source: PLoS One. 2020 Jun 2;15(6):e0233881. doi: 10.1371/journal.pone.0233881 (PMC7266318; doi:10.1371/journal.pone.0233881)

S2 Fig 1.

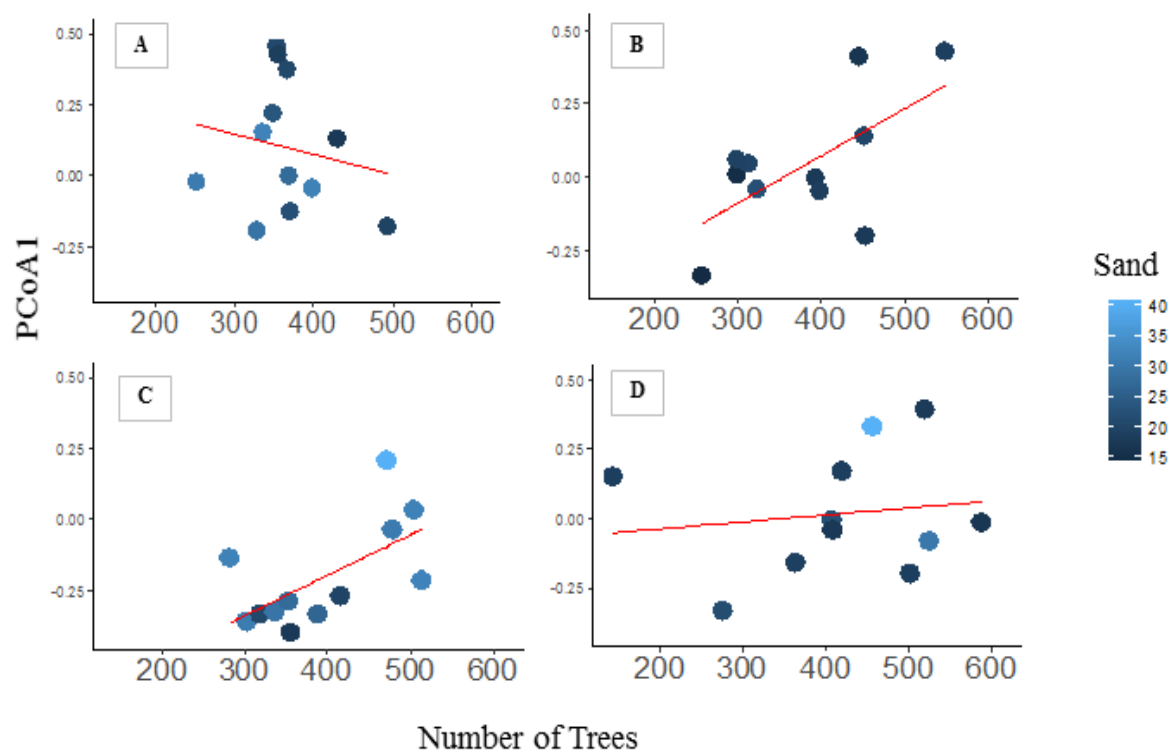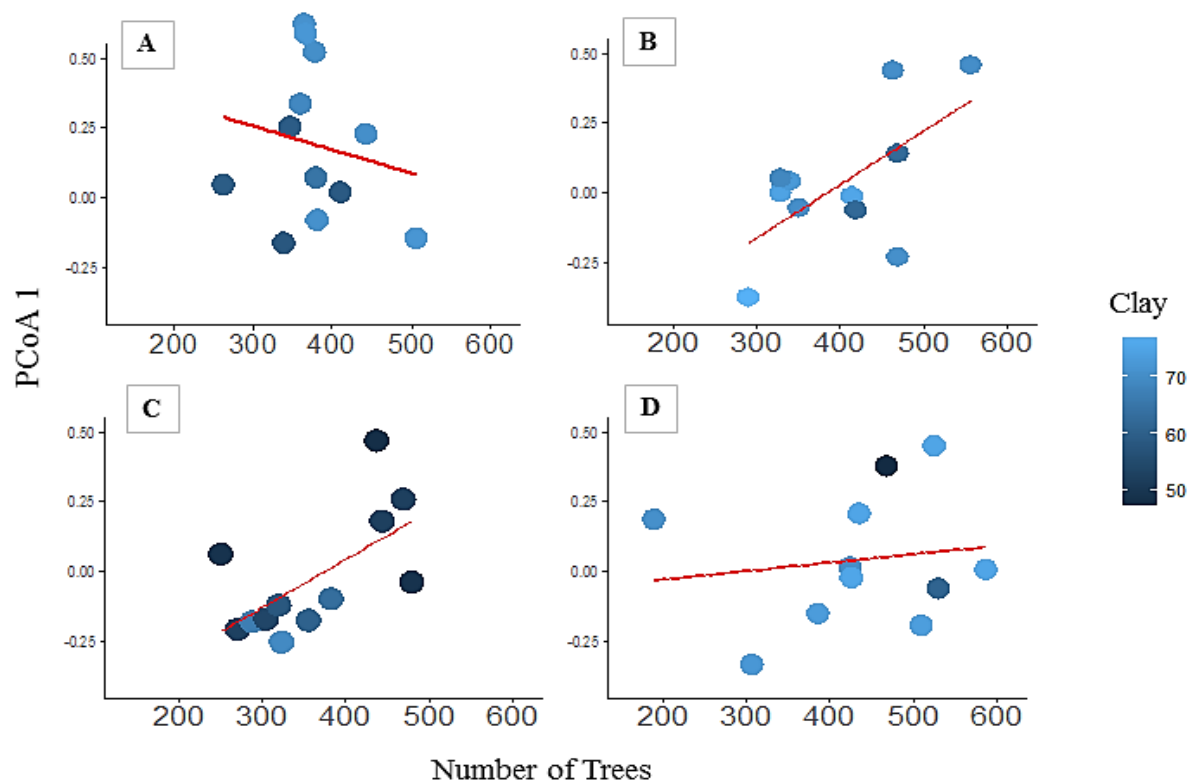

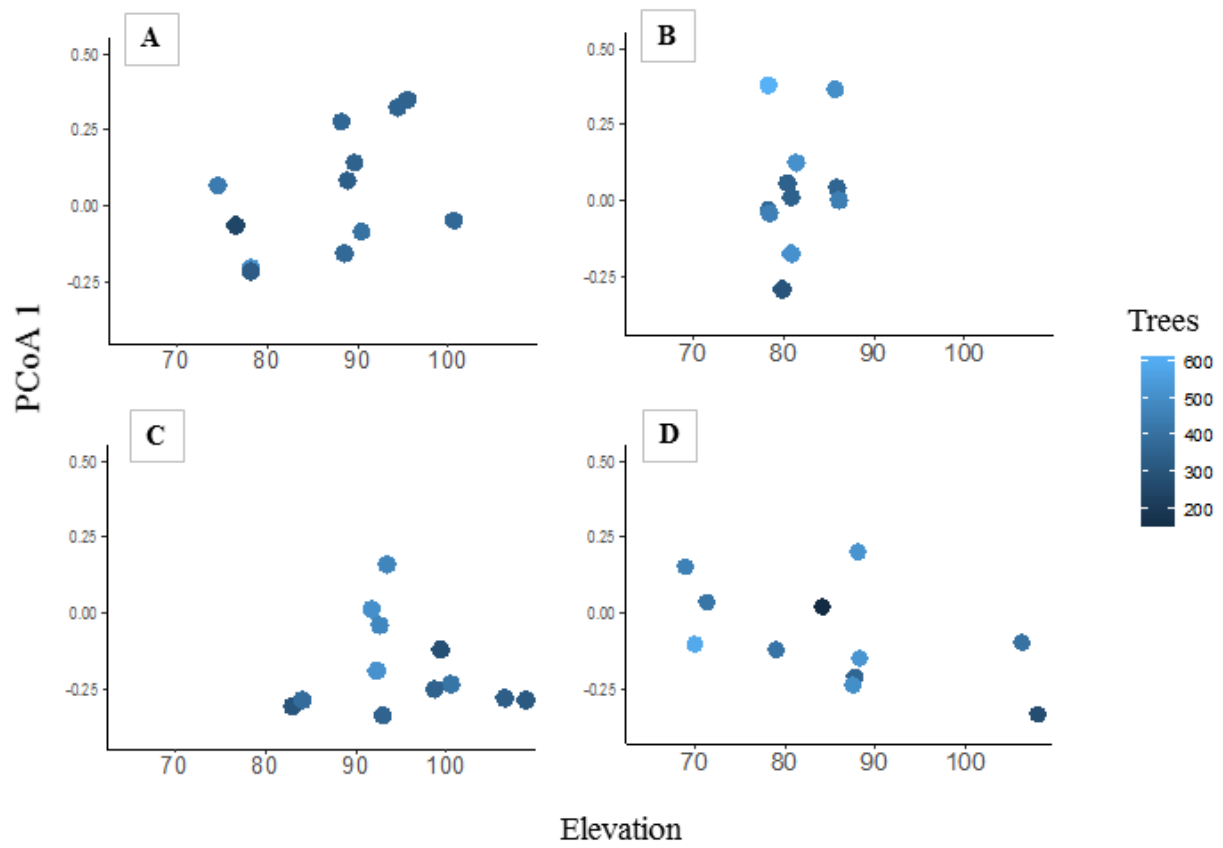

Supplement: S1 Fig — Effect of environmental predictors on lizard assemblages composition (PCoA axis 1). The models were selected by ΔAICc < 2. (A) Ilha das Pedras (B) Ilha dos Búfalos (C) West Jirau (D) Teotônio. (PDF) [file pone.0233881.s002.pdf]

S3 Fig 2.

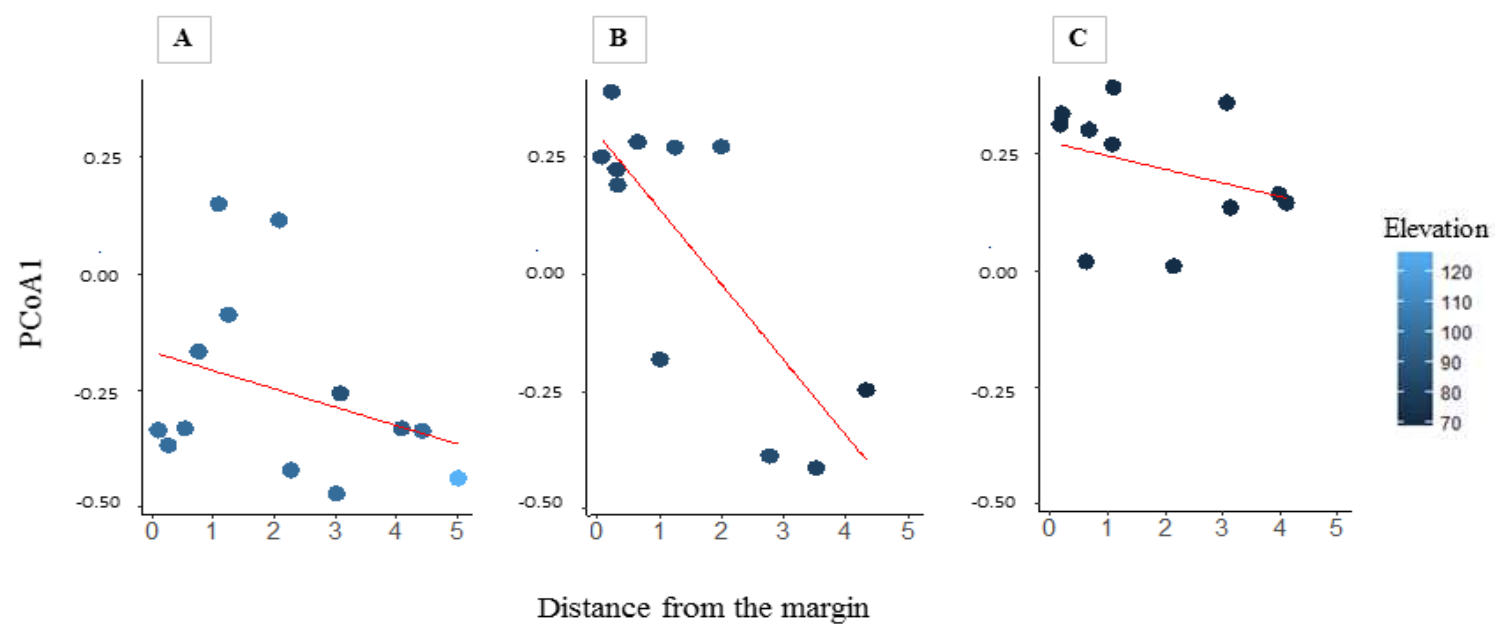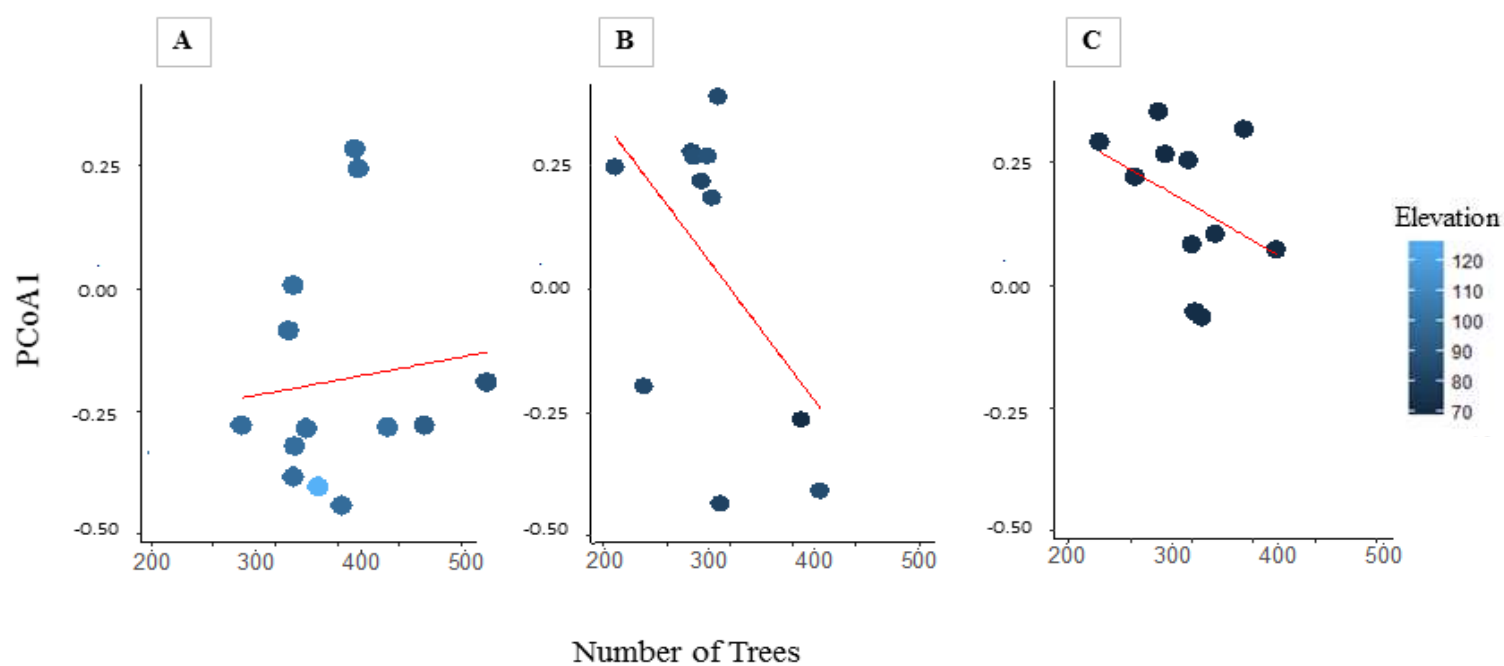

Supplement: S2 Fig — Effect of environmental predictors on lizard assemblages composition (PCoA axis 1). The models were selected by ΔAICc < 2. (A) Jaci-Paraná (B) East Jirau (C) Morrinhos. (PDF) [file pone.0233881.s003.pdf]
