# Supplementary material for: Hierarchical effects of historical and environmental factors on lizard assemblages in the upper Madeira River, Brazilian Amazonia
Source: PLoS One. 2020 Jun 2;15(6):e0233881. doi: 10.1371/journal.pone.0233881 (PMC7266318; doi:10.1371/journal.pone.0233881)

PLOS ONE

Hierarchical effects of historical and environmental factors on lizard assemblages in the upper Madeira river, Amazonian Brazil

--Manuscript Draft--

**Manuscript Number:**	PONE-D-19-15180R1
**Article Type:**	Research Article
**Full Title:**	Hierarchical effects of historical and environmental factors on lizard assemblages in the upper Madeira river, Amazonian Brazil
**Short Title:**	Effects of historical and environmental factors on lizard assemblages
**Corresponding Author:**	Gabriela Marques Peixoto, Ph.d candidate<br>Instituto Nacional de Pesquisas da Amazônia<br>Manaus, AM BRAZIL
**Keywords:**	Amazon forest; Environmental gradients; River barrier; reptiles; Squamata.
**Abstract:**	Investigating the role of historical and ecological factors structuring assemblages is relevant to understand mechanisms and processes affecting biodiversity across heterogeneous habitats. Considering that community assembly often involves scale-dependent processes, different spatial scales may reveal distinct factors structuring assemblages. In this study we use lizard abundance data from 83 plots to investigate assemblage spatial structure at two distinct scales in southwestern Amazonian Brazil. At a regional scale, we test the general hypothesis that the Madeira River acts as a barrier to dispersal of some lizard species, which results in distinct assemblages between river banks. At a local scale, we test the hypothesis that assemblages are not evenly distributed across heterogeneous habitats but respond to a continuum of inadequate-to-optimal portions of environmental gradients. Our results show that regional lizard assemblages are structured by the upper Madeira River acting as barrier to 29.62% of the species sampled. This finding suggests species have been historically isolated at one of the river banks, although the strength of the barrier may depend on the regional shape of the river. At a local scale, different sets of environmental gradients affected assemblage composition between river banks or even within a river bank. These findings indicate that environmental filtering is a major cause of lizard assemblage spatial structure in the upper Madeira River, but predictor variables cannot be generalized over the study area. Based on a single study system we demonstrate that lizard assemblages along the forests near the banks of the upper Madeira River are not randomly structured but respond to multiple factors acting at different and hierarchical spatial scales.
**Order of Authors:**	Gabriela Marques Peixoto, Ph.d candidate
	Rafael De Fraga
	Maria C. Araújo
	Igor Luis Kaefer
	Albertina Pimentel Lima
**Response to Reviewers:**	Responses to editor comments:<br>1. Please ensure that your manuscript meets PLOS ONE's style requirements, including those for file naming. The PLOS ONE style templates can be found at http://www.journals.plos.org/plosone/s/file?id=wjVg/PLOSOne_formatting_sample_main_body.pdf and http://www.journals.plos.org/plosone/s/file?id=ba62/PLOSOne_formatting_sample_title_authors_affiliations.pdf<br>Response: All PloS ONE style standards have been revised.<br>2. We note that Figure 1 in your submission contain [map/satellite] images which may be copyrighted. All PLOS content is published under the Creative Commons Attribution License (CC BY 4.0), which means that the manuscript, images, and Supporting Information files will be freely available online, and any third party is permitted to access, download, copy, distribute, and use these materials in any way, even commercially, with proper attribution. For these reasons, we cannot publish previously

Powered by Editorial Manager® and ProduXion Manager® from Aries Systems Corporation

copyrighted maps or satellite images created using proprietary data, such as Google software (Google Maps, Street View, and Earth). For more information, see our copyright guidelines: http://journals.plos.org/plosone/s/licenses-and-copyright.
We require you to either (1) present written permission from the copyright holder to publish these figures specifically under the CC BY 4.0 license, or (2) remove the figures from your submission:
Response: The satellite image used to compose Figure 1 was changed following the proposed guidelines and a new image was added in its place from The Gateway to Astronaut Photography of Earth (public domain).

Response to Reviewers

1.My main concern is that all inference here is based on count and presence/absence data, which may be influenced by sampling errors. For instance, you may find 20 individuals of species A and 19 individuals of species B in a given plot, when in fact there was 120 individuals of species A and 20 individuals of species B. This is typically a false-negative type error. Alternatively, you may have misidentified species B, inducing false-positive errors in your counts. Similar problems occur when using presence/absence data when absence is not included and estimated in the model. This subject is well covered in the literature. You may want to have a look at Kéry and Schmidt 2008 (Community Ecology 9: 207-216), Fitzpatrick et al. 2009 (Ecological Applications 19: 1673-79), Miller et al. 2012 (Ecological Applications 22: 1665-74) and so on. Since sampling effort seems to be enough, I recommend applying a modeling technique that accounts for sampling errors (for instance, N-mixture models), which I find more appealing to discuss results arising from count data. However, I understand that you would have to make a major change in your manuscript, especially if results change, which I would not be surprised. At least, I recommend including a paragraph discussing this subject, since I find hard to believe that you have captured the exactly number of individuals and species present at a given site, and thus, the results may be biased.

Response:

We agree with the reviewer that when we study ecological data based on abundance and presence / absence data, we may be masking sampling errors since every technique is biased and will depend on the probability of species detection. However, as in our study we conducted multiple data collections at every point, combined with more than one sampling method, we think that we minimized sampling bias as much as possible.
As for the suggested modeling technique (N-mixture models): we opted for the second option suggested by the reviewer, which would be to continue using the models already proposed and not change our analyzes. We add a paragraph discussing these type I and II errors and the possible biased results generated from the sampling and that could be altering our results in some way. We justify our decision not to use N-mixture models because the same consist of two linked generalized linear models, however, for the realization of this model, the ordering method chosen by us (principal coordinate analysis - PCoA) cannot be used since it does not support the input of negative values. Given that the PCoA returns negative values of scores on its axes we could not be using this sorting method. Therefore, we chose to continue with PcoA to the detriment of the other sorting methods that could be substituted to meet the assumption of generalized linear models, since our choices followed the one suggested by GOTELLI & ELLISON (2011, page 446 of the book Principles of Statistics in Ecology). These authors recommend the use of PcoA for “sorting applications in which the objective is to preserve the original multivariate distances between observations in the reduced space (sorting)”, especially when we intend to use the axes generated in the ordering in later analyzes. With regard to the use of other methods such as the widely used NMDS, the axes are not orthogonal, that is, we could not choose just one of the axes as a predictor of our species composition.
We carried out several previous analyzes with other sorting methods, different types of returns, and simulations including the removal of species that presented low abundance. In each simulation we had the precaution to show to other researchers in the area and request their opinions before deciding on the methods presented in this

version of our manuscript. Among these simulations, we carried out the NMDS which captured 46.64% of the variation in species composition with two axes and demonstrated that the lizard assemblages differed significantly in relation to the banks of the Madeira River (MANOVA: Pillai trace = 0,205, $F_{1,80}$= 10,229, p <0,001). As for the results of the regressions with the environmental variables, the ordering of the NMDS plots for the left side of the river was only possible using three NMDS axes due to the little variation in the data with two axes (which led us to prefer the analysis main coordinates - PcoA), and as a result captured 70% of the variation in species composition. The explanation for the right margin using two NMDS axes was 57%. As for the possible structuring environmental variables, the effect of the vegetation structure (number of trees) on the left side ($F_{7,38}$=2,567, p=0,04), and for the right margin, only elevation was identified as a factor influencing the composition of the lizard assemblages ($F_{1,70}$=7,737, p<0,001). With all the simulations with our data we can see a strong effect of the modules themselves and the need to analyze the modules Ilha da Pedra (west bank) and Jirau-D (east bank) separately from the rest of the sample units, so we arrive at our current results .

2.Your sample includes temporal variation (extended for a year, or so) but there are no temporal covariates among your predictors. Lizard activity is usually related to temperature, sunlight, rainfall and so on. You probably sampled plots in different days and different hours within a day, and ciclicity occurs even within individuals. How do you think the absence of temporal predictors affect your results? For instance, Figure 2 shows species ‘abundances on each river bank. Some species seems to be common on one bank, but not the other. Is this really the barrier effect of the river? By ignoring temporal variability, you assume sampling did not vary along the year.

Response:
Each sampling campaign was carried out at a different time of the year: Campaign I- 24 February to 26 April 2010; Campaign II- July 30 to August 19 2010; Campaign III- November 5 to 26 2010; and Campaign IV- January 13 to February 4 2011. In each of these campaigns, all plots inserted in the different modules were sampled, that is, each plot was sampled in four different times of the year. Therefore, we think we minimized the sampling bias regarding the presence / absence of species in the plots. In fact, the activity of the lizards is closely related to variations in temperature, light and precipitation in a given environment. With this in mind, the plots were sampled at different times. We believe that our sampling method that combined active visual search, with two simultaneous observers, supplemented by sweeping the leaf-litter, minimized the lack of temporal predictors in the presence/absence results.

3.Authors suggest that the pattern found – the river acting as a barrier – was important. However, they mention that the lizard fauna found is widely distributed over the Amazon. This is somewhat confusing to me because if species were well-distributed, they would probably be found everywhere. To explain that, authors mention that the barrier effect of the river may vary, suggesting seasonality. This makes me wonder if species are truly isolated, as suggested. The barrier effect could be only temporarily and the results found are explained by the time frame of the study. I would like authors to clarify that point.

Response:

Actually, most of the lizard species found in our study are widely distributed across Amazonia, and when we say that these species are isolated, we are not trying to extrapolate our results to the entire length of the river, so we were careful to keep that registered in the discussion (from line 385). as we do not have information for other locations along the length of the Madeira River.
We did not want to suggest seasonality when we wrote that the river barrier effect may vary. The different Amazonian rivers (and even different parts of a same large river) have distinct widhts, current speeds, water types and geological dynamics, which may affect their role as an effective geological barrier.
Even widely distributed species in Amazonia are not necessarily found everywhere, since the configuration of the Amazon Basin can be related to a mosaic of different phyto-regions, observed both on a local and regional scale, with different geological ages and origins between the different fractions of the basin, a fact that leads to historical evolutionary differences between areas and that become determinant factors

Powered by Editorial Manager® and ProduXion Manager® from Aries Systems Corporation

for the differences in lizard species richness in the region. Even within each area of endemism, with no current geographical barriers, ecological differentiation can occur (Ferrão et al., 2017; Ortiz et al. 2018). The isolation of lizard species in one of the banks of the river may be related to geomorphological heterogeneity along the course of the Madeira River, and even at different sampling dates, we still observe the restricted presence of some species to certain banks of the river.

Ferrão, M.; Moravec, J.; Fraga, R.; Almeida, A.P.; Kaefer, I.L.; Lima, A.P. 2017. A new species of Scinax from the Purus–Madeira interfluve, Brazilian Amazonia (Anura, Hylidae). ZooKeys, 706: 137–162.
Ortiz DA, Lima AP, Werneck FP (2018) Environmental transition zone and rivers shape intraspecific population structure and genetic diversity of an Amazonian rain forest tree frog. Evolutionary Ecology 32(4):359-378

4.I think your manuscript would benefit from contrasting your results with other lizard systems around the world. For instance, are there examples of rivers acing as barriers for lizards elsewhere? What about other reptiles? What are the covariates related to isolation for lizards or reptiles in general? In addition, you could provide some information about the Amazon basin. For instance, what is the mean width of the big rivers? Finally, concerning the species found. What is the habit of the most abundant species? Did you find terrestrial, arboreal and fossorial species equally? Does you results relate to species ‘habits? I believe that finding terrestrial species (the teiids, for instance) may be easier than arboreal (Enyalius?) or fossorial. In the same way, diurnal species will be easier to find during the day and the opposite for nocturnal. How was the sampling scheme? Can you really talk about lizards in general of a guild?

Response:
We thank the reviewer and agree that some information needs to be added, so we have included in the discussion other examples of lizards in the Amazon and around the world, and data on the covariates associated with restriction of geographic distributions. Information on the width of the largest Amazonian rivers was added to the methodology when mentioning the Madeira River, as it is one of the largest and most important rivers in the Amazon basin.
Regarding the species habits, we found the tree species Gonatodes humeralis, and Norops fuscuoratus as the most abundant, followed by the teiid Ameiva ameiva. The fossorial or semi-fossorial species were the ones with the lowest abundances, a fact already expected and observed in different studies on lizard assemblages. We think that the results are related to the habits of the species, as included in the discussion (starting from line 428), where we associate the results of the gradient number of trees to some tree species found.
Regarding the species activity time (more detail in the following answers to the reviewer below), most species are diurnal or crepuscular and few are nocturnal, but easily detectable to experienced researchers in the region.
We observed that our sample is within the richness already highlighted in the local and regional literature for Amazonia, and we think we gave a good representation of how the lizard community in that region is structured.
With regard to the term guild, we decided not to apply because the observed species use different and complementary resources, thus not constituting a specific guild with regard to diet or habitat use, for example.

Minor comments
✓
L62: substitute "design" by "designed"

Response: Replaced in text
✓
L84: change "the latter" by "lizards"
Response: Replaced in text
✓
L161: I am not sure I would exclude those sites since you may overestimate predictor effects.

Response: We decided to maintain the exclusion, as some of these sites coincided with areas of difficult access, for example, due to fallen trunks, making it difficult to travel along the plot, or even partially flooded, and could contribute to the low probability of detection. Thus, we decided to exclude all sites that contained zero individuals.

✓
L166-167: you have four surveys on each plot. I suppose there was variation on specie´s counts over surveys, due to several aspects, including seasonality (which you ignored). I advise on modeling this variability on counts.

Response: Question previously answered.
✓
L168-169: what time did you search? Are all lizards diurnal/nocturnal? Any potential bias here?
Response: Most of Amazonian lizards are diurnal. Even those that start their activity at dusk, such as Uranoscodon superciliosus (Linnaeus, 758), are easy to detect because they usually rest over tree branches while inactive. Regarding campaign schedules, all were carried out during the day. However, the first and last were carried out in the afternoon and early evening, and the second and third were carried out in the morning and early afternoon. Thus, we contemplated different activity periods, maximizing detectability.

✓
L177: I would rather call these 'environmental predictors', since gradients may remind other things as well.

Response: Replaced in text.
✓
L183: change '.' by ','
Response:Replaced in text.
✓
L221-223: maybe it is just me, but the way it is written seems that you avoided correlated models, not predictors.
Response: The sentence was rewritten for clarity.
✓
L223-226: what was the cut-off point?
Response: From r = 0.51. We added this information in the text.

✓L233: what do you mean by "corrected for few parameters"?

Response: We wanted to say that we did use Akaike´s Information Criterion (AIC) for model selection, but the one corrected for small samples: the AICc. The sentence was poorly written in the first version and rephrased for clarity.
✓
L234-235: what about the non-nested models? Why using the cut-off point if important model weight may be left out? Isn´t model averaging a better strategy depending on the case?
Response: I am not sure if I understood the question, and in what our analysis differs from what has already been done in the literature. We stipulated the ∆AICc <2 cut, due to the number of possible models with different combinations between the variables, and this cut would inevitably exclude some models at the expense of those more parsimonious models.

✓
L261-262: how can you be sure those species were not there?
Table 2. what kind of models did you build? Only those with main effects, excluding additive and interactive effects? Did you try combining all best predictors in one model?
Figure 3. which model did you use to plot the relationship between abundance and number of trees? The top model or all three models? Notice that model weights are similar and all of them include the number of trees.

Table 3: This table is somewhat hard to read. Please, insert a backspace between West and East or a line between them. In addition, maybe you could reduce the names of the predictors or show their first letters only.

Response: Yes, several models were built, with different combinations between different predictors that did not correlate with each other. In relation to figure 3, we show a direct ordination of the values from the gradient number of trees and the relative abundance of species by plots. With regard to the table, we added additional lines as suggested by the reviewer for a better visualization.
✓L293: again, I would rather call ‘predictor’ or ‘covariate’ instead of ‘gradient’.

Response: Replaced in text.
✓
L294: which model? Not the top model nor the third best-ranked model, if I read the table correctly (please, revise the table configuration). However, all models present similar AIC weights. How was the rationale behind using the only model that presented a significant effect? Did you try model averaging? I would not pick just one model among several to present a result, especially if they diverge.
Figure 4. Same comment as in Figure 3.
Response: We decided to graphically show only the significant models (P < 0.05). We could really include the images of all the models (which were marginally significant), but we wanted to be synthetic and illustrate these results with a single image due to the length of the article.

✓
L352: there is a typo in this line.
Response: Corrected in text.
✓
L389-390: I am not convinced about that without including temporal predictors in the analysis. Was it too hot or too cold during some of the samplings? Were there thunderstorms or heavy rainfall in, at least, some of the sampling occasions?
Response: Samples were not taken in the rain. When this happened the samplings were postponed.

✓
L400-405: I find the discussion on conservation implications interesting, but I am not sure you provided all the elements for that. For instance, what kind of forest did you measure, native on invasive? Were there pine plantations (which are also categorized as forests)? How is the relationship between lizard assemblage and forests in general?

Response: All sampling modules were installed in original, preserved Amazonian terra-firme forests that are not influenced by other types of vegetation.

✓
L419: you start this paragraph stating that species turnover was influenced by the distance from the river. But this was not found in all samples, as you stated in the same paragraph. Given that your samples covered only 5 km from the river, which in the Amazon, does not seem to be a huge area, what distance do you think you would find such effect?

Response: We believe that species turnover is related to several aspects in addition to the distance from the river, and that stipulating a margin distance value for the Amazon is a risk, since the region has a large environmental heterogeneity between the interfluvial regions, and even over small distances of 5km we could have significant results in the turnover of species, especially those less vagile. However, many gaps need to be filled for Amazonian lizards, despite recent efforts to answer such a question.
✓
L427-428: what do you mean by "...was returned..."?
Response: We used returned in the sense that it was observed, but the word was

	replaced in the text.<br><br>✓<br>L451-452: what do you mean by "efficient methods for multi-scale sampling"? Can you provide suggestions about what you would say is an efficient method?<br>Response: Methods that present a spatial standardization, easily replicable for different species (modular), and preferably that can be carried out for a long time. An example of a good method is the one used here in our study, the RAPELD- Rapid Assessments and Long-term Ecological Research (Magnusson et al., 2005). These modules are part of a network of permanent standardized transects installed in the Amazon by the Biodiversity Research Program (PPBio) of the Brazilian Science, Technology, Innovations and Communications Ministry (Magnusson et al., 2013). An example of sample desing is shown in the figure below. In this method the plots follow the terrain level curve, minimizing possible topographic (environmental) effects within the plot (sampling unit). RAPELD has already been used in several localities around the world https://ppbio.inpa.gov.br/sites/default/files/Biodiversidade_e_monitoramento_ambiental_integrado.pdf. cited 10 March 2019.
Additional Information:	
Question	Response
**Financial Disclosure**<br><br>Enter a financial disclosure statement that describes the sources of funding for the work included in this submission. Review the submission guidelines for detailed requirements. View published research articles from *PLOS ONE* for specific examples.<br><br>This statement is required for submission and **will appear in the published article** if the submission is accepted. Please make sure it is accurate.<br><br>**Unfunded studies**<br>Enter: *The author(s) received no specific funding for this work.*<br><br>**Funded studies**<br>Enter a statement with the following details:<br>• Initials of the authors who received each award<br>• Grant numbers awarded to each author<br>• The full name of each funder<br>• URL of each funder website<br>• Did the sponsors or funders play any role in the study design, data collection and analysis, decision to publish, or preparation of the manuscript?<br>• **NO** - Include this sentence at the end of your statement: *The funders had no role in study design, data collection and analysis, decision to publish, or preparation of the manuscript.*<br>• **YES** - Specify the role(s) played.	Data collection was financially supported by Programa de Conservação da Vida Selvagem da Santo Antônio Energia S.A.; Conselho Nacional de Desenvolvimento Científico e Tecnológico (CNPq) and Coordenação de Aperfeiçoamento de Pessoal de Nível Superior (CAPES) granted PhD scholarships to GMP. CAPES provided a PNPD postdoc grant to RF and CNPq provided productivity grants to ILK and APL. The funders had no role in study design, data collection and analysis, decision to publish, or preparation of the manuscript.

* typeset	
**Competing Interests**<br><br>Use the instructions below to enter a competing interest statement for this submission. On behalf of all authors, disclose any competing interests that could be perceived to bias this work—acknowledging all financial support and any other relevant financial or non-financial competing interests.<br><br>This statement **will appear in the published article** if the submission is accepted. Please make sure it is accurate. View published research articles from *PLOS ONE* for specific examples.<br><br>**NO authors have competing interests**<br><br>Enter: *The authors have declared that no competing interests exist.*<br><br>**Authors with competing interests**<br><br>Enter competing interest details beginning with this statement:<br><br>*I have read the journal's policy and the authors of this manuscript have the following competing interests: [insert competing interests here]*<br><br>* typeset	The authors have declared that no competing interests exist.
**Ethics Statement**<br><br>Enter an ethics statement for this submission. This statement is required if the study involved:<br><br>• Human participants<br>• Human specimens or tissue<br>• Vertebrate animals or cephalopods<br>• Vertebrate embryos or tissues<br>• Field research<br><br>Write "N/A" if the submission does not require an ethics statement.	All collected species were deposited at the herpetological collection of the National Institute of Amazonian Research (INPA-H) in the municipality of Manaus, state of Amazonas, Brazil. We collected data under RAN-ICMBio / IBAMA permit nº 13777-2.

General guidance is provided below. Consult the submission guidelines for detailed instructions. **Make sure that all information entered here is included in the Methods section of the manuscript.**<br><br>**Format for specific study types**<br><br>**Human Subject Research (involving human participants and/or tissue)**<br>• Give the name of the institutional review board or ethics committee that approved the study<br>• Include the approval number and/or a statement indicating approval of this research<br>• Indicate the form of consent obtained (written/oral) or the reason that consent was not obtained (e.g. the data were analyzed anonymously)<br><br>**Animal Research (involving vertebrate animals, embryos or tissues)**<br>• Provide the name of the Institutional Animal Care and Use Committee (IACUC) or other relevant ethics board that reviewed the study protocol, and indicate whether they approved this research or granted a formal waiver of ethical approval<br>• Include an approval number if one was obtained<br>• If the study involved *non-human primates*, add *additional details* about animal welfare and steps taken to ameliorate suffering<br>• If anesthesia, euthanasia, or any kind of animal sacrifice is part of the study, include briefly which substances and/or methods were applied<br><br>**Field Research**<br><br>Include the following details if this study involves the collection of plant, animal, or other materials from a natural setting:<br>• Field permit number<br>• Name of the institution or relevant body that granted permission	
**Data Availability**<br><br>Authors are required to make all data	Yes - all data are fully available without restriction

Powered by Editorial Manager® and ProduXion Manager® from Aries Systems Corporation

underlying the findings described fully available, without restriction, and from the time of publication. PLOS allows rare exceptions to address legal and ethical concerns. See the PLOS Data Policy and FAQ for detailed information.<br><br>A Data Availability Statement describing where the data can be found is required at submission. Your answers to this question constitute the Data Availability Statement and **will be published in the article**, if accepted.<br><br>**Important:** Stating 'data available on request from the author' is not sufficient. If your data are only available upon request, select 'No' for the first question and explain your exceptional situation in the text box.<br><br>Do the authors confirm that all data underlying the findings described in their manuscript are fully available without restriction?	
**Describe where the data may be found in full sentences. If you are copying our sample text, replace any instances of XXX with the appropriate details.**<br><br>• If the data are **held or will be held in a public repository**, include URLs, accession numbers or DOIs. If this information will only be available after acceptance, indicate this by ticking the box below. For example: *All XXX files are available from the XXX database (accession number(s) XXX, XXX.*).<br>• If the data are all contained **within the manuscript and/or Supporting Information files**, enter the following: *All relevant data are within the manuscript and its Supporting Information files.*<br>• If neither of these applies but you are able to provide **details of access elsewhere**, with or without limitations, please do so. For example:<br><br>*Data cannot be shared publicly because of [XXX]. Data are available from the*	All relevant data are within the manuscript and its Supporting Information files.

XXX Institutional Data Access / Ethics Committee (contact via XXX) for researchers who meet the criteria for access to confidential data.<br><br>*The data underlying the results presented in the study are available from (include the name of the third party and contact information or URL).*<br>• This text is appropriate if the data are owned by a third party and authors do not have permission to share the data.<br><br>* typeset	
Additional data availability information:	Tick here if your circumstances are not covered by the questions above and you need the journal's help to make your data available.

Powered by Editorial Manager® and ProduXion Manager® from Aries Systems Corporation

Manuscript
Click here to access/download;Manuscript;Manuscript-Peixoto et al_Lizard assemblages Madeira-02-03.docx

Hierarchical effects of historical and environmental factors on lizard assemblages in the upper Madeira river, Amazonian Brazil

Gabriela Marques Peixoto[1*¶], Rafael De Fraga[2¶], Maria C. Araújo[1¶], Igor Luis Kaefer[1,3¶], Albertina Pimentel Lima[1¶]

1 Pós-Graduação em Ecologia, Instituto Nacional de Pesquisas da Amazônia, Avenida André Araújo, Manaus, Amazonas, Brasil,

2 Pós-Graduação em Recursos Naturais Amazônicos, Universidade Federal do Oeste do Pará, Santarém, Pará, Brasil,

3 Instituto de Ciências Biológicas, Universidade Federal do Amazonas, Manaus, Amazonas, Brasil

***** Corresponding author

E-mail: gabriela.marquespd@gmail.com (GMP)

[¶]These authors contributed equally to this work.

Abstract

Investigating the role of historical and ecological factors structuring assemblages is relevant to understand mechanisms and processes affecting biodiversity across heterogeneous habitats. Considering that community assembly often involves scale-dependent processes, different spatial scales may reveal distinct factors structuring assemblages. In this study we use lizard abundance data from 83 plots to investigate assemblage spatial structure at two distinct scales in southwestern Amazonian Brazil. At a regional scale, we test the general hypothesis that the Madeira River acts as a barrier to dispersal of some lizard species, which results in distinct assemblages between river banks. At a local scale, we test the hypothesis that assemblages are not evenly distributed across heterogeneous habitats but respond to a continuum of inadequate-to-optimal portions of environmental predictors. Our results show that regional lizard assemblages are structured by the upper Madeira River acting as barrier to 29.62% of the species sampled. This finding suggests species have been historically isolated at one of the river banks, although the strength of the barrier may depend on the regional shape of the river. At a local scale, different sets of environmental predictors affected assemblage composition between river banks or even within a river bank. These findings indicate that environmental filtering is a major cause of lizard assemblage spatial structure in the upper Madeira River, but predictor variables cannot be generalized over the study area. Based on a single study system we demonstrate that lizard assemblages along the forests near the banks of the upper Madeira River are not randomly structured but respond to multiple factors acting at different and hierarchical spatial scales.

Introduction

Investigating historical and ecological factors structuring assemblages may reveal patterns of biodiversity distribution across time and space [1,2]. However, defining mechanisms and processes that potentially affect assemblage structure is often highly dependent on the spatial scale applied [3-5]. Such dependence results from the fact that assemblage composition (e.g. taxonomic diversity) is influenced by complex hierarchical interactions among processes that operate at multiple spatio-temporal scales [6]. In highly heterogeneous habitats such as the Amazonian tropical rainforests the relative contribution of historical and ecological processes to assemblage structuring is poorly understood for many taxa, mainly because multi-scale ecological approaches depend on standardized sampling systems, which have been specifically designed for such purpose [e.g. 7-12]. Regarding lizards, poor knowledge on assemblage structure also results from lack of refined data on individual species distribution [13], despite few unpublished studies have shown assemblage spatial structure defined by environmental heterogeneity [e.g. 14-16].

At broad spatial scales (e.g. Amazon Basin), it has been suggested that many organisms are restrictedly distributed by their inability to cross large rivers. From the classic studies of Alfred R. Wallace on primate distribution across the Amazon Basin [e.g. 17], it has been known that the Amazon River and some of its main tributaries (e.g. Madeira, Negro) may be important biogeographic barriers to dispersal. Testing the Wallace´s hypothesis has revealed the riverine barrier as a major factor explaining limited distribution of plants [18], birds [19-22], frogs [23,12], primates [24,25] and spiny rats [26]. Additionally, studies have shown that gene flow reduced or blocked by a riverine barrier may cause genotypic and phenotypic divergence in Amazonia [27-29]. Specifically for lizards, riverine barriers may cause intraspecific genetic divergence [27], although they do not necessarily produce different

morphotypes [30]. Interspecifically, species distribution regionally limited to a single river bank may cause distinct assemblage compositions between banks [12,31].

At local scales, environmental predictors may affect species occurrence and abundance due to the filter effect of the spatial variation in habitat suitability [32,33]. In general, it is expected that habitat-specialist species find inadequate-to-optimum continuums of environmental conditions for survival and reproduction [34]. Environmental filtering has been found in Amazonia for plants [35,36], frogs [37,38], birds [39,40], snakes [41,42] and lizards [43,15]. For lizards, local assemblages may differ due to variation in individual abundance or species turnover along gradients of distance from water courses [44,31], elevation [45], climate seasonality [46], and number of trees [47,43]. Additionally, lizard assemblages may be indirectly structured by species turnover along gradients of canopy openness affecting the availability of thermoregulation sites [48,49], understory-plant density affecting the availability of foraging sites for perching species [50], and clay content in the soil affecting plant composition and food availability [43].

Integrating multiple spatial scales is relevant to estimating simultaneous effects of historical and ecological factors on assemblage structure, especially in heterogeneous habitats such as rainforests in Amazonia [51]. However, designing a sampling system which is efficient to quantify assemblages and habitats at multiple scales may be challenging. The RAPELD [1] method (Brazilian acronym for rapid sampling plus long-term ecological research) has been shown to be efficient for this purpose in the region of the upper Madeira River [13], due to (i) the adequate distribution of plot sets (5 km^2 each) so that hypotheses based on the effects of historical factors on regional assemblages may be tested (e.g. riverine barriers), and (ii) the plots following altitudinal contours reduce within-plot environmental variation, which allows them to be assumed as environmental units to test hypotheses based on environmental filtering [1]. The rationale behind testing such hypotheses in southwestern

Amazonia is that the Madeira River has been recognized as a barrier to dispersal of Squamata reptiles, which causes species turnover along a longitudinal gradient [52], and the region covers two endemism zones (Rondônia e Inambari) that are distinct regarding geological history and environmental heterogeneity [53].

In this study we use plot-based lizard abundance data from the upper Madeira River (southwestern Amazonian Brazil) to investigate patterns of assemblage structure at two distinct spatial scales. At a regional scale, we test the hypothesis that lizard assemblages differ between the river banks. We expect differences in species composition and abundance as a consequence of the Madeira River historically limiting lizard dispersal. At local scale, we test the hypothesis that environmental heterogeneity causes species turnover, because species are absent or occur at low densities in suboptimal portions of environmental predictors. Specifically, we quantify the filtering effects on lizard abundance driven by gradients of number of trees, soil nutrient composition, shrub density, elevation, clay and sand content in the soil, and distance from the river bank. We expect that analyzing assemblages from two distinct perspectives will provide us with deep insights into factors that cause and maintain biodiversity at megadiverse regions such as the upper Madeira River.

Materials and methods

Study area

The study area is located near the banks of the upper Madeira River (coordinates of the centroid 08 ° 48004.0 "S; 63 ° 56059.8" W), an important tributary of the Amazon River classified as a white and muddy river with a total length of 1,459 km. The upper Madeira River extends from the outskirts of Porto Velho (state of Rondônia) to about 600 km upstream, in southwestern Amazonia. We also sampled plots close to the Jaci Paraná river, a tributary on the east bank of the upper Madeira river (Fig. 1).

Fig 1. Location of the upper Madeira River, state of Rondônia, Brazil. Sampling 5 km^2 modules (circles) near the banks. Gray circles show modules in the Inambari endemism zone, blue circles are modules in the Rondônia endemism zone [according to 20]. The acronyms summarize sampling modules´ local names: TO = Teotônio, IB = Ilha dos Búfalos, IP = Ilha das Pedras, JL = East Jirau, JR = West Jirau, JP = Jaci-Paraná, MO = Morrinhos. In detail on the left side, the standard configuration of each module, with 14 plots (squares), 250 m-long each, distributed along a gradient of distance from the river bank (0–5,000 m).

In this study we quantified environmental heterogeneity as continuous gradients that may be broadly classified for descriptive purposes in three main habitat types. They mainly differ in canopy height, soil texture, and understory-plant density and species composition [following 54]. In the upland (terra-firme) forests habitats are never flooded by overflowing large rivers, the canopy is 30 m high, and the understory-plant density and clay content in the soil often depends on elevation [55]. The várzea forests are seasonally flooded by overflowing sediment-rich rivers, which produces nutrient-rich soils that are water-saturated for long periods. The canopy is 20 m high, and the understory is rich in bromeliads. The campinaranas are patches of palm tree-rich forests growing on a white-sand soil, which is highly drained and nutrient-poor [54].

The climate of the study area is tropical humid, with annual average temperature at 25.5 °C and average precipitation at 2,287 mm. Precipitation is distributed throughout the year in well-marked dry (May to September) and rainy (October to April) seasons. During the dry season, small streams can dry completely [56].

Sampling design

We collected lizard abundance data in seven 5 km^2 RAPELD sampling sites (hereinafter modules), that were installed perpendicularly to the river bank. RAPELD [1] is a

modification of the Gentry´s sampling method based on 1-ha plots [57], with the main difference being that the RAPELD plot central lines follow the altitudinal curves to reduce environmental variation within plots (PPBio - http://ppbio.inpa.gov.br). We sampled three modules on the east bank of the Madeira River (East-Jirau, Jaci-Paraná and Morrinhos), and four modules on the west bank (West-Jirau, Ilha das Pedras, Ilha dos Búfalos and Teotônio). The average distance between neighboring modules was 120 km. Each RAPELD module was composed of two 5-km long parallel trails, separated by 1 km. We surveyed seven 250 m plots (20 m wide) on each trail, totaling 98 plots (14 plots in each of the seven modules). The plots were distributed along a gradient of distance from the river bank, at 0, 500, 1000, 2000, 3000, 4000 and 5000 m.

We were not able to find lizards in 15 plots, and the excess of zeros in the dataset prevented us to reliably estimate pairwise distances among plots to summarize assemblage composition (see Data analysis). Therefore, we excluded zero-valued plots and our analyzes are based on 83 plots.

Sampling effort

We sampled each plot in four different periods (24 February to 26 April 2010, ~~July 30~~ to ~~August 19~~ 2010, ~~November 5~~ to 26 2010, and ~~January 13~~ to ~~February 4~~ 2011) to cover large portions of the regional variation in temperature and precipitation along a year. We used species' maximum abundance values per plot in the analyzes.

We found lizards using active visual search, with two simultaneous observers positioned 10 m apart. In addition, we supplemented the sampling effort by sweeping the leaf litter and removing debris in a 2 m strip following the center line of the plot. This approach was particularly useful to increase the efficiency of sampling fossorial and leaf-litter species (e.g. Alopoglossidae, Gymnophthalmidae). The searching time in each plot varied between 40 and 60 minutes.

Environmental variables

We measured eight environmental predictors in each plot in order to quantify spatial heterogeneity in habitat suitability. We quantified vegetation structure by measuring (i) number of trees and (ii) shrub density. Those predictors potentially affect squamates abundance by influencing availability of foraging, resting and thermoregulation sites [58-60]. We also measured edaphic gradients related to soil texture, fertility, and flat-level deviation, which are (iii) clay content, (iv) sand content, (v) nutrient composition (Soil pH, Calcium, Magnesium, Potassium, Zinc and exchangeable Aluminum), (vi) elevation, and (vii) terrain declivity. Those variables potentially affect lizard abundance by causing variation in the overall primary production [61]. and availability of invertebrate prey [62]. Additionally, we measured (viii) distance from the river bank, because it has been found as a major factor structuring plant [36] and animal [31,38,39,41] assemblages in Amazonia. The methods used to measure each predictor are described in detail in Appendix 1.

Data analysis

To quantify assemblage composition, we applied the Bray-Curtis index to estimate pairwise distances in species abundance among plots. We reduced dimensionalities using Principal Coordinate Analysis (PCoA) and represented assemblage composition by the first one or two axes produced (see below).

At regional scale (riverine barrier effects) we modeled the PCoA using all data (83 plots). The two first axes captured 30% (PCoA 1 = 16%. PCoA 2 = 14%) of the original variance in species abundance, and we used them to represent assemblage composition. To assess assemblage structuring, we used Multivariate Analysis of Variance MANOVA to test differences in assemblage composition (PCoA axes 1 and 2) between the river banks. We implemented a MANOVA using the vegan [63] R-package [64].

Analyzes at regional scale revealed two distinct lizard assemblages between the river banks (see Results). In addition, preliminary analyzes at local scale revealed that in two modules (Ilha das Pedras and East Jirau) environmental predictors may affect assemblage composition in opposite directions compared to the other modules (S2 and S3 Fig). These findings suggested that the banks of the Madeira River and some of the sampling modules ~~within a~~ river bank are distinct environmental units, which contain distinct spatial structures of lizard assemblage composition. Therefore, to assess assemblage structure at local scale we modeled four distinct PCoA ordinations, using data from (i) the west bank, except for the module Ilha das Pedras (37 plots), which captured 86% of the original variance (PCoA 1 = 0.50, PCoA 2 = 0.36); (ii) the east bank, except for the module East Jirau (23 plots), which captured 45% of the original variance (PCoA 1 = 0.30, PCoA 2 = 0.15); (iii) the module Ilha das Pedras (12 plots), which captured 45% of the original variance (PCoA 1 = 0.32, PCoA 2 = 0.13); and (iv) the module East Jirau (11 plots), which captured 85% of the original variance (PCoA 1 = 0.49, PCoA 2 = 0.36).

The environmental predictors measured are expressed in different units and therefore in different orders of magnitude, so we transformed them using the “scale” function of the vegan R-package. This function subtracts mean values from each variable and scales centralized variables by dividing them by their standard deviation [63]. We used Mixed Linear Models to test the effects of scaled environmental predictors on assemblage composition based on data from multiple sampling modules. By using this method, we were able to include sampling modules as random effects to minimize potential abrupt differences in environmental predictors and lizard assemblages among the modules analyzed in a same model [65]. We set up two different groups of mixed models, according to the assemblage compositions summarized by PCoA for the west and east banks of the Madeira River. Each group was composed of as many models as necessary to test all possible combinations of

environmental predictors, except for those predictors that were highly correlated. For instance, clay and sand content in the soil were not used in a same model because they were highly correlated on both river banks ($r \geq 0.93$). In addition, elevation was correlated with terrain declivity on both river banks ($r \geq 0.78$) and soil-nutrient composition on the east bank ($r = 0.66$). The cut-off point was $r = 0.51$.

For the two modules that were analyzed separately (Ilha das Pedras and East Jirau), it was not necessary to control random effects of sampling sites, so we tested the effects of environmental predictors on the assemblage composition using multiple linear regression models. We tested models with assemblage composition (PCoA 1) as dependent variable, and all possible combinations of uncorrelated environmental predictors as independent variables.

To select the most parsimonious mixed-effects and multiple-regression models we ranked all the models by the corrected Akaike´s Information Criterion (AICc) [66]. We refined the model selection by penalizing nested models assuming ΔAICc < 2 as a cut-off point. All selected models were validated by normal distribution of residuals (Shapiro-Wilk $W > 0.95$, $P > 0.05$ in all cases).

For visually checking the distribution of lizard abundance values per species along river banks and environmental predictors (only those that significantly affected assemblage composition) we plotted ordinated sampling plots. These graphs will be used in this study for assessing how spread the distributions of abundance values are over the river banks and the environmental heterogeneity measured.

RESULTS

We found 27 lizard species, which are classified in 18 genera and 10 families. The most frequently found species were *Norops fuscoarautus* (Dactyloidae), *Gonatodes humeralis* (Sphaerodactylidae), and *Ameiva ameiva* (Teiidae), which occurred in both banks of the

Madeira River, in 55, 49 and 30% of the plots respectively. Contrarily, *Alopoglossus angulatus* (Alopoglossidae) and *Enyalius leechii* (Leiosauridae) were found in one single plot (Table 1).

Table 1. List of lizard species sampled in the upper Madeira River, Brazil. N = total abundance per species, East and West = Madeira river banks filled with presence (1) and absence (0) data.

**Family/Species**	**N**	**East**	**West**
**Dactyloidae**			
Norops fuscoauratus (D'Orbigny, 1847)	103	1	1
Norops tandai (Wagler, 1830)	2	0	1
Norops ortonii (Cope, 1869)	2	1	1
Dactyloa punctata (Daudin, 1802)	27	1	1
Dactyloa transversalis (Dumeril, 1851)	9	0	1
**Alopoglossidae**			
Alopoglossus angulatus (Linnaeus, 1758)			
**Gymnophthalmidae**	2	0	1
Arthrosaura reticulata (O'Shaughnessy, 1881)	5	1	0
Cercosaura argulus (Peters, 1863)	5	1	1
Cercosaura eigenmanni (Griffin, 1917)	11	1	1
Cercosaura bassleri (Ruibal, 1952)	8	0	1
Iphisa elegans (Gray, 1851)	8	1	1
Loxopholis percarinatum (Muller, 1923)	10	1	1
**Hoplocercidae**			
Enyalioides laticeps (Guichenot, 1855)	3	1	1
Hoplocercus spinosus (Fitzinger, 1843)	2	1	1
**Leiosauridae**			
Enyalius leechii (Boulenger,1885)	2	1	0
**Scincidae**			
Copeoglossum nigropunctatum (Spix, 1825)	14	1	1
**Phyllodactylidae**			
Thecadactylus rapicauda (Houttuyn, 1782)	21	1	1
**Sphaerodactylidae**			
Chatogekko amazonicus (Andersson, 1918)	12	1	1
Gonatodes hasemani (Griffin, 1917)	29	1	1
Gonatodes humeralis (Guichenot, 1855)	432	1	1

**Teiidae**			
Kentropyx altamazonica (Cope, 1876)	14	1	1
Kentropyx calcarata (Spix, 182)	37	1	0
Kentropyx pelviceps (Cope, 1868)	29	0	1
Ameiva ameiva (Linnaeus, 1758)	48	1	1
**Tropiduridae**			
Plica plica (Linnaeus, 1758)	7	1	1
Plica umbra ochrocollaris (Spix, 1825)	21	1	1
Uranoscodon superciliosus (Linnaeus, 758)	5	1	1
**Total**	**868**		

Regional assemblage structuring - Madeira River as a biogeographic barrier

We found 19 species on both banks of the Madeira River, which is equivalent to 70.37% of the total diversity sampled. This finding suggests that most of the species sampled are widely distributed throughout the study area. However, for several of the species found on both sides of the river (e.g. *Loxopholis percarinatum*, *Kentropyx altamazonica*, *Cercosaura eigenmanni*, *Plica plica*, *Uranoscodon superciliosus*, *Copeoglossum nigropunctatum*), plot-related frequency and abundance were not even between the river banks (Fig 2). Additionally, five species (18.52%) were restricted to the west bank – *Alopoglossus angulatus*, *Norops tandai*, *Dactyloa transversalis*, *Cercosaura bassleri* and *Kentropyx pelviceps*, and three species (11.11%) were restricted to the east bank –*Arthrosaura reticulata*, *Kentropyx calcarata* and *Enyalius leechii*. These findings suggest two distinct assemblage compositions delimited by the Madeira River, which is strongly supported by differences in the PCoA scores (based on 83 plots) between the river banks (MANOVA Pillai Trace = 0.315, $F_{1–81}$ = 18.40, P <0.001).

Fig 2. Plots ordinated according to their position of the upper Madeira River (west and east). The heights of the black rectangles are relative to species abundance values.

Local assemblage structuring – the role of environmental predictors

On the west bank of the Madeira River (except for the module Ilha das Pedras) three mixed-effects models were selected by ΔAICc < 2 (Table 2). All the selected models consistently returned number of trees as a major gradient affecting assemblage composition ($P < 0.001$ in all cases). Despite some species occupied large portions of the gradient of number of trees (e.g. *Ameiva ameiva*, *Norops fuscoauratus*), species absence or low abundance in specific intervals between 144 and 613 trees caused species turnover (Fig 3). According to the models selected, assemblage composition was not affected by elevation ($P = 0.76$), and soil-content of sand ($P = 0.84$) or clay ($P = 0.91$).

Table 2. **Summary of the results returned by Linear Mixed-Effects Models.** The models were set up using data from the west (Teotônio, Ilha dos Búfalos and West Jirau) and east (Morrinhos and Jaci-Paraná) banks of the upper Madeira River. The models were selected by AICc Δ < 2. Shapiro-Wilk tests were applied on the residuals from each model to test normality. Bolded p-values show cases in which the null hypothesis was rejected.

**Margins**	**Fixed effects**	**AICc**	**Weight**	***df***	***t***	***p***	**Total variance**	**Shapiro-Wilk**
**West**	Number of Trees and Elevation	12.89	0.314	Intercept:2.18	-6.92	<**0.001**	54%	P=0.109
				Trees:3.01	18.8	<**0.001**		
				Elevation:1.43	-0.31	0.76		
	Sand and Number of Trees	12.88	0.309	Intercept:3.39	-14.69	**0.001**	69%	P=0.066
				Sand:1.86	-0.19	0.84		
				Trees:3.29	18.8	<**0.001**		

	Clay and Number of Trees	12.88	0.305	Intercept:1.00	-9.07	<**0.001**	76%	P=0.153
				Clay:9.76	0.10	0.91		
				Trees:3.27	18.88	<**0.001**		
**East**	Elevation and Margin distance	2.0	0.412	Intercept:2.30	6.37	<**0.001**	71%	P=0.782
				Elevation:2.30	-6.27	<**0.001**		
				Margim:2.30	1.72	0.09		
	Number of Trees and Elevation	2.0	0.400	Intercept:2.30	5.92	<**0.001**	72%	P=0.413
				Trees:2.10	18.8	0.10		
				Elevation:2.30	-0.31	<**0.001**		

Fig. 3. Plots ordinated according to their position relative the number of trees measured in the west bank of the upper Madeira River, state of Rondônia, Brazil. The heights of the black rectangles depict the relative species abundances.

Three multiple-regression models were selected for Ilha das Pedras sampling module (Table 3), all of them containing elevation as an independent variable. This predictors significantly affected assemblage composition according to a model constructed with soil sand content as an additional independent variable (P = 0.05) (Fig 4). However, the effects of elevation on the assemblage composition were marginally significant in models containing number of trees (P = 0.06) and soil clay content (P = 0.07) as independent variables.

Table 3. Summary of the results returned by Linear Mixed-Effects Models. The models were set up using data from the Ilha das Pedras (west river bank) and East Jirau (east river bank) modules to test the effects of environmental predictors on lizard assemblage composition. The models were selected by AICc Δ < 2. Shapiro-Wilk tests were applied on the residuals from each model to test normality. Bolded p-values show cases in which the null hypothesis was rejected.

**Margins**	**Variables**	**AICc**	**Weight**	**Std. error**	***t***	**P**	***F***	**r2**
West	Number of Trees and Elevation	15.7	0.31	Intercept:8.76	0.00	1.00	2.746	0.37
				Trees:9.19	0.90	0.39		
				Elevation:9.19	-2.06	0.06		
	Sand and Elevation	15.8	0.29	Intercept:8.81	0.00	1.00	2.66	0.37
				Sand:9.21	0.85	0.42		
				Elevation:9.21	-2.18	**0.05**		
	Clay and Elevation	16.2	0.25	Intercept: 1.21	0.1	1.10	2.45	0.35
				Clay:6.12	-0.64	0.53		
				Elevation:1.88	-1.98	0.07		
East	Clay and Distance from the margin	6.5	0.655	Intercept: 5.69	0.00	1.00	15.42	0.81
				Clay: -1.05	-1.69	0.13		
				Margim;2.89	4.63	<**0.001**		
	Sand and	7.9	0.367	Intercept: 2.30	5.92	<**0.001**	13.07	0.78

	Distance from the margin	Sand:8.81	1.28	0. 24
		Margim:2.85	4.15	<**0.001**

Fig. 4. Partials from a multiple linear model for the Ilha das Pedras module. Model for the effects from the elevation and sand contents in the soil on lizard assemblage composition. Assemblage composition was summarized by the first axis of an ~~Analysis of~~ Principal Coordinates based on abundance data of the upper Madeira River, state of Rondônia, Brazil. The shades of blue show values of sand content in the soil.

On the east river bank (except for the East Jirau module) two models were selected as most parsimonious. Both models consistently showed strong effects of elevation on assemblage composition (P < 0.001 in both cases). This finding suggests species turnover along an elevational gradient of 69.12–100.59 m (Fig 5). According to the same models, distance from the river bank (P = 0.09) and number of trees (P = 0.1) did not affect assemblage composition.

Fig 5. Plots ordinated according to their position relative to a gradient of elevation (meters above the sea level) in the east bank of the upper Madeira River, state of Rondônia, Brazil. The heights of the black rectangles depict the relative species abundances.

Two multiple-regression models were selected for the East Jirau module. Both models consistently returned distance from the river bank (Fig 6) as a relevant gradient affecting assemblage composition (P < 0.001 in both cases). Soil-content of sand (P = 0.24) and clay (P = 0.13) did not affect assemblage composition.

Fig 6. Partials from a multiple linear model from the East-Jirau module. The effects of distance from the river bank, sand and clay contents in the soil on lizard assemblage composition. Assemblage composition was summarized by the first axis of an ~~Analysis of~~ Principal Coordinates based on abundance data from the East Jirau sampling module, located on the east bank of the upper Madeira River, state of Rondônia, Brazil. The shades of blue show values of sand and clay contents in the soil.

Discussion

At regional scale, we found that lizard assemblages are spatially structured by differences in assemblage composition between river banks. This finding is consistent with large Amazonian rivers acting as dispersal barriers for several organisms, which have caused different species subsets composed of plants [18], birds [19,21], primates [24] and diurnal frogs [12]. At local scale, we showed that lizard assemblages are spatially structured by species turnover along environmental predictors. However, a set of environmental predictors cannot be assumed as generalized predictors among sampling sites. Our overall results are broadly consistent with frog assemblages sampled in the same plots [12], which suggests multi-taxa ecological patterns. We relied on a single dataset to provide understanding about assemblage structure based on interacting historical and ecological processes. Therefore, we highlight the relevance of investigating multi-scale assemblage structuring for ecology and conservation decision making.

In the upper Madeira River, assemblage divergence between river banks has been attributed to historical processes regionally reducing species dispersal [12]. Approximately half of the species present in the assemblage of diurnal frogs (13 species) of that region were restricted to one of the river banks [12]. The smaller proportion of regionally isolated lizard

species (29.63%) is reasonably explained by the lower dispersal capacity of small and site-attached frogs compared with most lizards. Nonetheless, we investigated aseemblages in which about 30% of the sampled species were isolated by the river, and another 30% of the species occurred at low relative frequency or abundance at one of the river banks. This was a sufficiently adequate scenario to assume the river as a historical factor segregating assemblages between the river banks. We highlight that most of regionally isolated species in our sample are widely distributed throughout Amazonia outside our study area [13]. Such inconsistency may be explained by the strength of the river as a dispersal barrier varying along the river course, or even being nullified in response to meandering shapes [67- 69,30]. Additionally, the barriers may be seasonal, because bridges for stepping-stone dispersal may be revealed during the dry season, which allows gene flow between river banks [70]. Therefore, our results for assemblage structure at regional scale should not be extrapolated to unsampled stretches of the Madeira River or other Amazonian rivers, because lizards probably have found multiple dispersal routes through evolutionary time [27].

The isolation of species on one of the river banks may be related to the geomorphological heterogeneity of the Madeira River across our study area. The Madeira river flows over an incisive fluvial valley, with predominantly crystalline and a geologically ancient basement (ca. 16 Ma). The morphodynamical development was mainly influenced by the geomorphological and climatic changes resulting from the Andean Orogeny in the Cenozoic [71], which have produced a relatively stable course along recent geological times [72]. Such stability in the shape of the river course has prevented meandering across most of the study area, which could facilitate for species to cross the river [73]. Exceptionally, the modules located further upstream (East and West Jirau) have rocky outcrops that are exposed in the middle of the river course during the dry season, which can act as bridges for stepping-stone dispersal (field observation). Although lizard species used alternative dispersal routes

to widespread their distribution throughout Amazonia, our study showed that they were regionally prevented from colonizing or maintaining populations on both banks of the upper Madeira River. One could argue that our results of a river-barrier effect are biased due to the low detection probability of lizards, which resulted in false absence of species [74,75]. However, we think that a possible sampling bias was mitigated by the large sampling effort associated to the combination of different sampling methods employed in this study.

Besides affecting assemblage composition, the effect of rivers as barriers can also be observed at the intraspecific level in different biogeographic domains, resulting in genetic and morphological divergence among lizard populations due to restriction to gene flow [30,76,77]. Studies on the genetic and phenotypic differentiation of populations of a same species on opposite banks of the Madeira River should be performed as they might help to understand the initial steps of allopatric speciation in Amazonian lizards.

At local scale, lizard assemblages were spatially structured by environmental filtering causing non-random assemblage composition. Environmental conditions selected species that were unable to survive and maintain viable conditions in given sampling plots [78]. Despite we sampled species that are generalist in relation to the environmental predictors measured (e.g. *Ameiva ameiva*, *Norops fuscoauratus*), species for which distributions were restricted to narrow regions of gradients (e.g. *Cercosaura argulus*, *Norops ortonii*, *Uranoscodon superciliosus*) caused species turnover across sampling plots. Species turnover mediated by environmental filtering is a major factor structuring local assemblages in Amazonia [e.g. 41,36,39], and in the upper Madeira River it has efficiently explained assemblage structure in bats [79], frogs [12] and snakes [80]. However, we cannot generalize a single environmental dataset as a predictor for assemblage composition in all plots. Environmental predictors for assemblage composition differed between the river banks or even within a river bank. This finding suggests that the scale at which lizard assemblages respond to environmental

heterogeneity may be more refined than the classification of the Madeira river banks as distinct endemism zones [81,20].

Number of trees was a major factor causing species turnover in the west bank of the Madeira River. This gradient ranged from 144 to 613 trees, which shows that the vegetation structure is quite heterogeneous throughout our study area. Heterogeneity in vegetation structure affects species occurrence and abundance due to variation in the availability of foraging, nesting, resting, and thermoregulating sites [58,60]. Additionally, tree cover may directly affect food availability, protection against predators, light intensity, temperature, humidity and wind speed [59,60]. The evidence for assemblage structuring along a gradient of number of trees is of concern from a conservation point of view, because our study area has been intensely deforested by the agribusiness and large hydroelectric plants [82]. It is widely expected that species dependent on high levels of tree cover (e.g. *Norops tandai*, *Norops ortonii*, *Dactyloa transversalis*) will either be locally extinct or migrate to more suitable habitats.

We found species turnover along an elevational gradient, although this finding was most evident on the east bank of the Madeira River. On the east bank the plots were installed on the depression of the Ji-Paraná river, which generated elevation values below 30 m. Low elevation is often related to outcropping of groundwater and high drainage density [83,71], which favors the occurrence of habitat-specific species for high humidity. For instance, *Arthrosaura reticulata* and *Uranoscodon superciliosus* typically occupy humid low areas [84,85], and in this study those species were found only on the east bank of the Madeira River. Additionally, elevation indirectly influences assemblage composition because it affects water availability and soil fertility [86-87], and therefore the overall structure of available habitats [88,89]. Extreme variation in elevation may cause behavioral and morphological differentiation in lizards [90]. In this study we showed that even subtle variation in elevation

(24 to 128 m) may be sufficient for species to be locally filtered. A similar finding was observed using frog assemblage data from the Guiana Shield [37].

The gradient of distance from the river caused species turnover in the East Jirau module. Although habitats may be classified in riparian and non-riparian zones [91], gradients of distance from water courses carry multiple continuous interacting variables of microclimate, nutrient availability, vegetation cover and edaphic structure. Habitats continuously changing along gradients of distance from streams (< 12 m wide) have caused species turnover structuring plant [36], frog [38], bird [39] and snake [41] assemblages. We have shown a similar pattern using lizard abundance data, with the main difference being that the gradient we measured refers to the distance from the bank of one of the major tributaries of the Amazon River. However, no significant effect of distance from the river on assemblage composition was observed using data from the other modules. This finding suggests that assemblages diverging between riparian and non-riparian zones should not be generalized in relation to gigantic rivers, or assemblage segregation should occur at distances that are greater than 5 km away from the river bank.

Some of the results found may be associated to environmental variables that were not explicitly measured in this study. For example, *Hoplocercus spinosus* (Hoplocercidae) occurred on both banks of the Madeira River but occurrence was restricted to plots with rocky outcrops. Such condition was only found in the westernmost sampling modules of the study area (East and West Jirau), where the species finds optimal availability of thermoregulation and refuge sites [91]. This finding reflects relationships between species and habitats that are dependent of biological traits affecting survival [92,93] and dispersal capacity [94,95], such as body size, diet [96], specificity level in habitat use [89], reproductive [49] and foraging mode [97]. Therefore, although patterns of assemblage structure are usually described based

on dissimilarities among plots regarding subsets of cooccurring species, they may be determined by ecological requirements of individual species.

We have shown that lizard assemblages in the upper Madeira River are structured by scale-dependent hierarchical factors. Historical processes related to the Andes uplift [98] have isolated regional assemblages between the river banks, and have also generated distinct habitat patches, which in turn generate distinct local lizard assemblages. It is generally well established that interacting historical and environmental factors explain hierarchical structures of assemblages [5]. However, empirical application is not common because it relies on efficient sampling designs to capture multiple scales [1]. In the megadiverse Amazonian rainforests this has been achieved by a few studies [73,12,31]. Considering the fine levels in which those studies have understood processes affecting biodiversity, efficient methods for multi-scale sampling should be prioritized by ecology and conservation biology.

Acknowledgments

Data collection was logistically supported by Programa de Pesquisas em Biodiversidade (PPBio), Centro de Estudos Integrados da Biodiversidade Amazônica (INCT-CENBAM), and Programa de Conservação da Vida Selvagem da Santo Antônio Energia S.A. Conselho Nacional de Desenvolvimento Científico e Tecnológico (CNPq) and Coordenação de Aperfeiçoamento de Pessoal de Nível Superior (CAPES) granted PhD scholarships to GMP. CAPES provided a PNPD postdoc grant to RF and CNPq provided productivity grants to ILK and APL. We collected data under RAN-ICMBio / IBAMA permit nº 13777-2.

References

1. Magnusson WE, Braga-Neto R, Pezzini F, Baccaro F, Bergallo H, Penha J, et al. Biodiversidade e monitoramento ambiental integrado: o sistema Rapeld na Amazônia. First ed. Santo André- SP: Attema Editorial; 2013. Available from: https://ppbio.inpa.gov.br/sites/default/files/Biodiversidade_e_monitoramento_ambiental_integrado.pdf. cited 10 March 2019.
2. Pianka ER. Ecology and natural history of desert lizards: analyses of the ecological niche and community structure. New Jersey. Princeton University Press; 1986.
3. Schneider DC. The rise of the concept of scale in Ecology. Bioscience. 2001; 51: 545–556. doi: 10.1641/0006-3568(2001)051[0545:TROTCO]2.0.CO;2.
4. Gaston KJ, Blackburn TM. Macroecology and conservation biology. In: Blackburn TM, Gaston KJ, editors. Macroecology: Concepts and consequences. Oxford: Blackwell Science; 2003. pp. 345–367.
5. Vellend M. Conceptual synthesis in community ecology. Q Rev Biol. 2010; 85: 183–206. doi: 10.1086/652373; PMID: 20565040.
6. Antonelli A, Quijada-Mascareñas A, Crawford AJ, Bates JM, Velazco PM, Wüster W. Molecular studies and phylogeography of Amazonian tetrapods and their relation to geological and climatic models. In: Hoorn C, Wesselingh FP, editors. Amazonia, landscape and species evolution: a look into the past: Blackwell Publishing; 2010. pp. 386–404.
7. Buhrnheim CM, Cox-Fernandes C. Structure of fish assemblages in Amazonian Rain-Forest Streams: Effects of habitats and locality. Copeia. 2003; 2: 255–262. doi: 10.1643/0045-8511(2003)003[0255:SOFAIA]2.0.CO;2.

8. Barros OG, Cintra R. The effects of forest structure on occurrence and abundance of three owl species (Aves: Strigidae) in the Central Amazon Forest. Rev Bras Zoo. 2009; 26: 86–95. doi: 10.1590/S1984-46702009000100014.
9. Costa FRC, Magnusson WE, Luizão RC. Mesoscale distribution patterns of Amazonian understorey herbs in relation to topography, soil and watersheds. J Ecol. 2005; 93: 863–878. doi: 10.1111/j.1365-2745.2005.01020.x.
10. Haugaasen T, Peres CA. Mammal assemblage structure in Amazonian floodedand unflooded forests. J Trop Ecol. 2005; 2: 133–145. doi: 10.1017/S026646740400207X.
11. Menin M, Waldez F, Lima AP. Effects of environmental and spatial factors on the distribution of anuran species with aquatic reproduction in central Amazonia. Herpetol J. 2011; 21: 255–261.
12. Dias-Terceiro RG, Kaefer IL, Fraga R, Araújo MC, Simões PI, Lima AP. A matter of scale: historical and environmental factors structure anuran assemblages from the upper Madeira River, Amazonia. Biotropica. 2015; 47: 259–266. doi: 10.1111/btp.12197.
13. Ribeiro-Júnior MA. Catalogue of distribution of lizards (Reptilia: Squamata) from the Brazilian Amazonia. I. Dactyloidae, Hoplocercidae, Iguanidae, Leiosauridae, Polychrotidae, Tropiduridae. Zootaxa. 2015; 3983: 001–110. doi: 10.11646/zootaxa.3983.1.1.
14. Lobão PSP. Associações no uso do habitat por cinco espécies de lagartos amazônicos. M.Sc. Thesis, Instituto Nacional de Pesquisas da Amazônia. 2008. Available from: https://bdtd.inpa.gov.br/handle/tede/731. cited 10 March 2019.
15. Moraes LFP. Diversidade beta em comunidades de lagartos em duas Ecorregiões distintas na Amazônia. M.Sc. Thesis, Instituto Nacional de Pesquisas da Amazônia,

Manaus. 2008. Available from: https://bdtd.inpa.gov.br/handle/tede/736. cited 10 March 2019.

16. Bittencourt S. A insularização como agente de fragmentação florestal em comunidades de lagartos da Amazônia Central. M.Sc. Thesis, Instituto Nacional de Pesquisas da Amazônia. 2008. Available from: https://bdtd.inpa.gov.br/handle/tede/1846. cited 15 March 2019.
17. Wallace AR. On the monkeys of the Amazon. J Nat Hist. 1954; 14 (2): 451–454. doi: 10.1080/037454809494374.
18. Dexter KG, Terborgh JW, Cunningham CW. Historical effects on beta diversity and community assembly in Amazonian trees. PNAS. 2012; 109: 7787–7792. doi: 10.1073/pnas.1203523109.
19. Hayes FE, Sewlal JN. The Amazon River as a dispersal barrier to passerine birds: effects of river width, habitat and taxonomy. J Biogeogr. 2004; 31: 1809–1818. doi: 10.1111/j.1365-2699.2004.01139. x.
20. Ribas CC, Aleixo A, Nogueira ACR, Miyaki CY, Cracraft J. A palaeobiogeographic model for biotic diversification within Amazonia over the past three million years. Proc Bio Sci. 2012; 279: 681–689. doi: 10.1098/rspb.2011.1120.
21. Pomara LY, Ruokolainen K, Young KR. Avian species composition across the Amazon River: the roles of dispersal limitation and environmental heterogeneity. J Biogeogr. 2014; 41: 784–796. doi: 10.1111/jbi.12247.
22. Fernandes AM, Gonzalez J, Wink M, Aleixo A. Multilocus phylogeography of the Wedge-billed Woodcreeper *Glyphorynchus spirurus* (Aves, Furnariidae) in lowland Amazonia: Widespread cryptic diversity and paraphyly reveal a complex diversification pattern. Mol Phylogenet Evol. 2013; 66(1): 270–282. doi: 10.1016/j.ympev.2012.09.033.

23. Simões PI, Lima AP, Magnusson WE. Acoustic and morphological differentiation in the frog *Allobates femoralis*: Relationships with the upper Madeira river and other potential geological barriers. Biotropica. 2008; 40: 607–614. doi: 10.1111/j.1744-7429.2008.00416.x.

24. Ayres JM, Clutton-Brock TH. River boundaries and species range size in Amazonian primates. Am Nat. 1992; 140: 531–537. doi: 10.1086/285427.

25. Boubli JP, Ribas C, Alfaro JWL, et al. Spatial and temporal patterns of diversification on the Amazon: A test of the riverine hypothesis for all diurnal primates of Rio Negro and Rio Branco in Brazil. Mol Phylogenet Evol. 2015; 82: 400–412. doi: 10.1016/j.ympev.2014.09.005.

26. Patton JL, da Silva MNF, Malcolm JR. Gene genealogy and differentiation among arboreal spiny rats (Rodentia: Echimyidae) of the Amazon Basin: a test of the riverine barrier hypothesis. Evol Lett. 1994; 48: 1314–1323. doi: 10.1111/j.1558-5646.1994.tb05315.x.

27. Ávila-Pires TC, Mulcahy DG, Werneck FP, Sites-Jr JW. Phylogeography of the teiid lizard *Kentropyx calcarata* and the Sphaerodactylid *Gonatodes humeralis* (Reptilia: Squamata): testing a geological scenario for the lower Amazon-Tocantins Basins, Amazonia, Brazil. Herpetologica. 2012; 68: 272–287. doi: 10.1655/HERPETOLOGICA-D-11-00021.1.

28. Kaefer IL, Tsuji-Nishikido BM, Mota EP, Farias IP, Lima AP. The early stages of speciation in Amazonian forest frogs: phenotypic conservatism despite strong genetic structure. Evol Biol. 2013; 40: 228–245. doi: 10.1007/s11692-012-9205-4.

29. Maia GF, Lima AP, Kaefer IL. Not just the river: genes, shapes, and sounds reveal population-structured diversification in the Amazonian frog *Allobates tapajos*

(Dendrobatoidea). Biol J Linn Soc Lond. 2017; 121: 95–108. doi: 10.1093/biolinnean/blw017.

30. Souza SM, Rodrigues MT, Cohn-Haft M. Are Amazonia rivers biogeographic barriers for lizards? A study on the geographic variation of the spectacled lizard *Leposoma osvaldoi* Ávila-Pires (Squamata, Gymnophthalmidae). J Herpetol. 2013; 47:511–519. doi: 10.1670/12-124.
31. Moraes LJ, Pavan D, Barros MC, Ribas CC. The combined influence of riverine barriers and flooding gradients on biogeographical patterns for amphibians and squamates in south-eastern Amazonia. J Biogeogr. 2016; 43(11): 2113–2124. doi: 10.1111/jbi.12756.
32. Mariac C, Jehin L, Saïdou AA, Thuillet AC, Couderc M, Sire P, et al. Genetic basis of pearl millet adaptation along an environmental gradient investigated by a combination of genome scan and association mapping. Mol Ecol. 2011; 20(1): 80–91. doi: 10.1111/j.1365-294X.2010.04893.x.
33. Hangartner S, Laurila A, Räsänen K. Adaptive divergence in moor frog (*Rana arvalis*) populations along an acidification gradient: inferences from QST–FST correlations. Evol Letters. 2012; 66(3): 867–881. doi: 10.1111/j.1558-5646.2011.01472.x.
34. Kinupp VF, Magnusson WE. Spatial patterns in the understorey shrub genus Psychotria in central Amazonia: effects of distance and topography. J Trop Ecol. 2005; 21: 363–374. doi: 10.1017/S0266467405002440.
35. Emilio T, Quesada CA, Costa FRC, Magnusson WE, Schietti J, Feldpausch TR, et al. Soil physical conditions limit palm and tree basal area in Amazonian forests. Plant Ecol Divers. 2013; 7: 215–229. doi: 10.1080/17550874.2013.772257.

36. Drucker DP, Costa FRC, Magnusson WE. How wide is the riparian zone of small streams in tropical forests? A test with terrestrial herbs. J Trop Ecol. 2008; 24: 65–74. doi: 10.1017/S0266467407004701.
37. Ribeiro-Jr JW, Lima AP, Magnusson WE. The effect of Riparian Zones on species diversity of frogs in Amazonian Forests. Copeia. 2012; 3: 375–381. doi: 10.1643/CE-11-117.
38. Rojas-Ahumada DP, Landeiro VL, Menin M. Role of environmental and spatial processes in structuring anuran communities across a tropical rain forest. Austral Ecol. 2012; 37: 865–873. doi: 10.1111/j.1442-9993.2011.02330.x.
39. Bueno AS, Bruno RS, Pimentel TP, Sanaiotti TM, Magnusson WE. The width of riparian habitats for understory birds in an Amazonian forest. Ecol Appl. 2012; 22(2): 722–734. doi: 10.1890/11-0789.1.
40. Menger J, Magnusson WE, Anderson MJ, Schlege M, Pe'er G, Henle K. Environmental characteristics drive variation in Amazonian understorey bird assemblages. PLoS ONE. 2017; 2(2): e0171540. doi: 10.1371/journal.pone.0171540.
41. Fraga R, Lima AP, Magnusson WE. Mesoscale spatial ecology of a tropic snake assemblage: the width of riparian corridors in central Amazonia. Herpetol J. 2011; 21: 51–57.
42. Fraga R, Ferrão M, Stow AJ, Magnusson WE, Lima AP. Different environmental gradients affect different measures of snake β-diversity in the Amazon rainforests. PeerJ. 2018; 6: e5628. doi: 10.7717/peerj.5628.
43. Pinto MGM. Diversidade Beta, métodos de amostragem e influência de fatores ambientais sobre uma comunidade de lagartos na Amazônia Central. M.Sc. Thesis,

Instituto Nacional de Pesquisas da Amazônia. 2006. Available from: https://bdtd.inpa.gov.br/handle/tede/947. cited 15 March 2019.

44. Faria AS, Menin M, Kaefer IL. Riparian zone as a main determinant of the structure of lizard assemblages in upland Amazonian forests. Austral Ecol. 2019; 1: 1–9. doi: 10.1111/aec.12754.
45. Fauth JF, Crother BI, Slowinski EJB. Elevational patterns of species richness, evenness, and abundance of the Costa Rican leaf-litter herpetofauna. Biotropica.1989; 21: 178–185. doi: 10.2307/2388708.
46. Lieberman SS. Ecology of a leaf litter herpetofauna of a neotropical rain litter in functioning of forest ecosystems. Biol Rev. 1986; 81: 1–31.
47. Driscoll DA. Extinction and outbreaks accompany fragmentation of a reptile community. Ecol Appl. 2004; 14: 220–240. doi: 10.1890/02-5248.
48. Vitt LJ, Blackburn DG. Ecology and life history of the viviparous lizard *Mabuya bistriata* (Scincidae) in the Brazilian Amazon. Copeia. 1999; 916–927. doi: 10.2307/1446087.
49. Vitt LJ, Zani PA, Lima AP. Heliotherms in tropical rain forest: the ecology of *Kentropyx calcarata* (Teiidae) and *Mabuya nigropunctata* (Scincidae) in the Curuá-Una of Brazil. J Trop Ecol. 1997; 13: 199–220. doi: 10.1017/S0266467400010415.
50. Dixo MBO. Efeito da fragmentação da floresta sobre a comunidade de sapos e lagartos de serapilheira no sul da Bahia. M.Sc. Thesis. Instituto de Biociências da Universidade de São Paulo. 2001.
51. Aleixo A. Knowledge gaps, research priorities, and future perspectives on bird conservation in the Brazilian Amazon. In: De Luc AC, Devele PE, Benck GA, Goerck JM, editors. Áreas importantes para a Conservação das Aves no Brasil. Parte

II-Amazônia, Cerrado e Pantanal. 1nd ed. São Paulo -SP: Save Brasil; 2009. pp. 59-69.

52. Ávila-Pires TCS, Vitt LJ, Sartorius SS, Zani PA. Squamata (Reptilia) from four sites in southern Amazonia, with a biogeographic analysis of Amazonian lizards. Bol Mus Para Emílio Goeldi Cienc Nat. 2009; 4(2): 99–118.
53. Schietti J, Martins D, Emilio T, Souza PF, Levis C, Baccaro FB. Forest structure along a 600 km transect of natural disturbances and seasonality gradients in central-southern Amazonia. J Ecol. 2016; 104(5): 1335–1346. doi: 10.1111/1365-2745.12596.
54. Cavalcante MMA. Hidroelétricas do Rio Madeira - RO: território, tecnificação e meio ambiente. M.Sc. Thesis, Universidade Federal do Paraná. 2012.
55. IBGE Instituto Brasileiro de Geografia e Estatística. Recursos naturais e meio ambiente: uma visão do Brasil. Rio de Janeiro: IBGE; 1997. Available from: http://biblioteca.ibge.gov.br/biblioteca-catalogo.html?id=27704&view=detalhes. cited 10 April 2019.
56. Bezerra RB, Trindade AG. Caracterização de parâmetros pluviométricos, térmicos do balanço hídrico climatológico e desmatamento de Porto Velho – RO. Geografia. 2006; 15(1): 65-80. doi: 10.5433/2447-1747.2006v15n1p65.
57. Gentry AH. Changes in plant community diversity and floristic composition on environmental and geographical gradients. Ann Mo Bot Gard. 1988; 75: 1–34. doi: 10.2307/2399464.
58. Burger J, Zappalorti RT. Nest site selection by Pine Snakes, *Pituophis melanoleucus*, in the New Jersey Pine Barrens. Copeia.1986; (1): 116–121.

59. Webb JK, Shine R. Out on a limb: conservation implications of tree-hollow use by a threatened snake species (*Hoplocephalus bungaroides*: Serpentes, Elapidae). Biol Conserv. 1997; 81(12): 21–33. doi: 10.1016/S0006-3207(96)00160-7.
60. Pringle RM, Webb JK, Shine R. 2003. Canopy structure, microclimate, and habitat selection by a nocturnal snake, *Hoplocephalus bungaroides*. Ecology. 2003; 84(10): 2668–2679. doi: 10.1890/02-0482.
61. Cintra BBL, Schietti J, Emilio T, Martins D, Moulatlet G, Souza P, et al. Soil physical restrictions and hydrology regulate stand age and wood biomass turnover rates of Purus-Madeira interfluvial wetlands in Amazonia. Biogeosciences. 2013; 10: 7759–7774. doi: 10.5194/bg-10-7759-2013.
62. Franklin E, Magnusson WE, Luizão FJ. Relative effects of biotic and abiotic factors on the composition of soil invertebrates' communities in an Amazonian savannah. Appl Soil Ecol. 2005; 29: 259–273. doi: 10.1016/j.apsoil.2004.12.004.
63. Oksanen J, Blanchet FG, Friendly M, Kindt R, Legendre P, McGlinn D, et al. Vegan: Community Ecology Package. R package version 1.17-1. 2010. Available from: http://CRAN.Rproject.org/package=vegan. cited 2 March 2019.
64. R Core Team. R: A language and environment for statistical computing. Vienna, Austria: R Foundation for Statistical Computing. 2019. Available from: https://cran.r-project.org/doc/manuals/r-release/fullrefman.pdf. cited 2 March 2019.
65. Laird NM, Ware JH. Random-effects models for longitudinal data. Biometrics. 1982; 38: 963–974.
66. Hurvich CM, Tsai CL. Regression and time series model selection in small samples. Biometrics. 1989; 76(2): 297–307. doi: 10.1093/biomet/76.2.297.

67. Gascon C, Lougheed SC, Bogart JP. Genetic and morphological variation in *Vanzolinius discodactylus*: a test of the river hypothesis of speciation. Biotropica. 1996; 28: 376–387. doi: 10.2307/2389201.

68. Smith AL, Bull CM, Gardner MG, Driscoll DA. Life history influences how fire affects genetic diversity in two lizard species. Mol Ecol. 2014; 23: 2428–2441. doi: 10.1111/mec.12757.

69. Dambros CS, Azevedo RA, Gotelli NJ. Isolation by distance, not rivers, control the distribution of termite species in the Amazonian rain forest. Ecography. 2016; 39: 1–9. doi: 10.6084/m9.figshare.1319582.v10.

70. Gibbs HL, Sovic M, Amazonas D, Chalkidis H, Salazar-Valenzuela D, Moura-Da-Silva AM. Recent lineage diversification in a venomous snake through dispersal across the Amazon River. Biol J Linn Soc Lond. 2018; 123(3): 651–665. doi: 10.1093/biolinnean/blx158.

71. Souza-Filho PWM, Quadros MLES, Scandolara JE, da Silva-Filho EP, Reis MR. Compartimentação morfoestrutural e neotectônica do sistema fluvial Guaporé-Mamoré-alto Madeira, Rondônia-Brasil. Braz J Geol. 1999; 29: 469–476. doi: 11005/10461.

72. Tikuka MM. Geoarqueologia e paleohidrologia da planície aluvial holocênica do rio Madeira entre Porto Velho e Abunã/RO. Amazon. Rev Antrop. 2012; 4: 252–257.

73. Gascon C, Malcolm JR, Patton JL, da Silva MNF, Bogart JP, Lougheed SC, et al. Riverine barriers and the geographic distribution of Amazonian species. PNAS. 2000; 97: 13672–13677. doi: 10.1073/pnas.230136397.

74. Williams BK, Nichols JD, Conroy MJ. Analysis and Management of Animal Populations. Acad Press. 2002; 1–817.

75. Miller DA, Weir LA, McClintock BT, Grant EHC, Bailey LL, Simons TR. Experimental investigation of false positive errors in auditory species occurrence surveys. Ecol App. 2012; 1665-1674.

76. Ansari MH, Cooper SJ, Schwarz MP, Ebrahimi M, Dolman G, Reinberger L, Gardner MG. Plio-Pleistocene diversification and biogeographic barriers in southern Australia reflected in the phylogeography of a widespread and common lizard species. Mol phylogen evol, 2019; 107–119.

77. Lamborot M, Eaton L. The Maipo River as a biogeographical barrier to Liolaemus monticola (Tropiduridae) in the mountain ranges of central Chile. J Zool Syst Evol. 1997;105–111.doi: 0947-5745/97/3503-0 105$14.00/0.

78. Kraft NJB, Adler PB, Godoy O, James EC, Fuller S, Levine JM. Community assembly, coexistence and the environmental metaphor. Funct Ecol. 2014; 29: 592–599. doi: 10.1111/1365-2435.12345.

79. Bobrowiec PED, Tavares VdC. Establishing baseline biodiversity data prior to hydroelectric dam construction to monitoring impacts to bats in the Brazilian Amazon. PloS ONE. 2017; 12(9): e0183036. doi: 10.1371/journal.pone.0183036.

80. Fraga RD, Lima AP, Magnusson WE, Ferrão M, Stow AJ. Contrasting patterns of gene flow for Amazonian snakes that actively forage and those that wait in ambush. J Heredity. 2017; 108(5): 524–534. doi: 10.1093/jhered/esx051.

81. Silva JMC, Rylands AB, Fonseca GAB. The fate of the Amazonian areas of endemism. Conserv Biol. 2005; 19: 689–694. doi: 10.1111/j.1523-1739.2005.00705.x.

82. Fearnside MP. Brazil's Madeira River dams: A setback for environmental policy in Amazonian development. Water Altern. 2014; 7: 256–269.

83. Bernardi JVE, Lacerda LD, Dórea JG, et al. Aplicação da análise das componentes principais na ordenação dos parâmetros físico-químicos no alto Rio Madeira e afluentes, Amazônia Ocidental. Geochim Brasiliensis. 2012; 23: 79–90.
84. Hoogmoed MS, Ávila-Pires TC. Studies on the species of the South American lizard genus *Arthrosaura Boulenger* (Reptilia: Sauria: Teiidae), with the resurrection of two species. Zool Meded. 1992; 66(35): 453–484.
85. Vitt LJ, Zani PA, Espósito MC. Historical ecology of Amazonian lizards: implications for community ecology. Oikos. 1999; 87: 286–294. doi: 10.2307/3546743.
86. Daws M, Mullins C, Burslem D, Paton S, Dalling J. Topographic position affects the water regime in a semideciduous tropical forest in Panama. Plant Soil Environ. 2002; 238: 79–90. doi: 10.1023/A:1014289930621.
87. Fischer J, Lindenmayer DB. The sensitivity of lizards to elevation: A case study from south-eastern Australia. Divers Distrib. 2005; 11: 225–233. doi: 10.1111/j.1366-9516.2005.00139.x.
88. Pansonato MP, Costa FRC, Castilho CV, Zuquim G. Spatial scale or amplitude of predictors as determinants of the relative importance of environmental factors to plant community structure. Biotropica. 2013; 45: 299–307. doi: 10.1111/btp.12008.
89. Tews J, Brose U, Grimm V, Tielborger K, Wichmann MC, Schwager M, et al. Animal species diversity driven by habitat heterogeneity/diversity: the importance of keystone structures. J Biogeogr. 2004; 31: 79–92. doi: 10.1046/j.0305-0270.2003.00994.x.
90. Ballinger RE. Intraspecific variation in demography and life history of the lizard, *Sceloporus jarrovi*, along an altitudinal gradient in southeastern Arizona. Ecology. 1979; 60: 901–909. doi: 10.2307/1936858.

91. Naiman RJ, Decamps H, Pollock M. The roles of riparian corridors in maintaining regional biodiversity. Ecol Appl. 1993; 3: 209–212. doi: 10.2307/1941822.

92. Rocha CFD. Introdução à ecologia de lagartos brasileiros. In: Nascimento LB, Bernerdes AT, Cotta GA, editors. Herpetologia no Brasil. 1nd ed. Minas Geirais, BR: PUCMG, Fundação biodiversidade and Fundação Ezequiel Dias; 1994. pp. 1–68.

93. Richardson JL. Divergent landscape effects on population connectivity in two co-occurring amphibian species. Mol Ecol. 2012; 21: 4437–4451. doi: 10.1111/j.1365-294X.2012.05708.x.

94. Fouquet A, Courtois EA, Baudain D, Lima JD, Souza SM, Noonan DP, et al. The trans-riverine genetic structure of 28 Amazonian frog species is dependent on life history. J Trop Ecol. 2015; 31: 361–373. doi: 10.1017/S0266467415000206.

95. Brandt R, Navas CA. Life-history evolution on Tropiduridae lizards: influence of lineage, body size and climate. PLoS ONE. 2011; 6: e20040. doi: 10.1371/journal.pone.0020040.

96. Cole EM, Bustamante MR, Almeida-Reinoso D, Funk WC. Spatial and temporal variation in population dynamics of Andean frogs: Effects of forest disturbance and evidence for declines. Glob Ecol Conserv. 2014; 1: 60–70. doi: 10.1016/j.gecco.2014.06.002.

97. Vitt LJ, Carvalho CMD. Life in the trees: the ecology and life history of *Kentropyx striatus* (Teiidae) in the Lavrado area of Roraima, Brazil, with comments on the life histories of tropical teiid lizards. Can J Zool. 1992; 70(10): 1995–2006. doi: 10.1139/z92-270.

98. Hoorn C, Wesselingh FP, Ter Steege H, Bermudez MA, Mora A, Sevink J, et al. Amazonia through time: Andean uplift, climate change, landscape evolution, and biodiversity. Science. 2010; 330(6006): 927–931. doi: 10.1126/science.1194585.

Supporting information captions

S1 Text. Protocols for measuring the environmental predictors**.** Predictors used as independent variables in the ecological models to test lizard assemblage structuring in the upper Madeira River, Amazonian Brazil.

S2 Fig 1. Partials from Mixed Linear Models for the west bank of the Madeira River. Test the effects of environmental predictors on lizard assemblages composition (PCoA axis 1). The models were selected by ΔAICc < 2. (A) Ilha das Pedras (B) Ilha dos Búfalos (C) ~~Jirau-West~~ (D) Teotônio.

S3 Fig 2. Partials from Mixed Linear Models for the East bank of the Madeira River. Test the effects of environmental predictors on lizard assemblages composition (PCoA axis 1). The models were selected by ΔAICc < 2. (A) Jaci-Paraná (B) ~~Jirau-East~~ (C) Morrinhos.

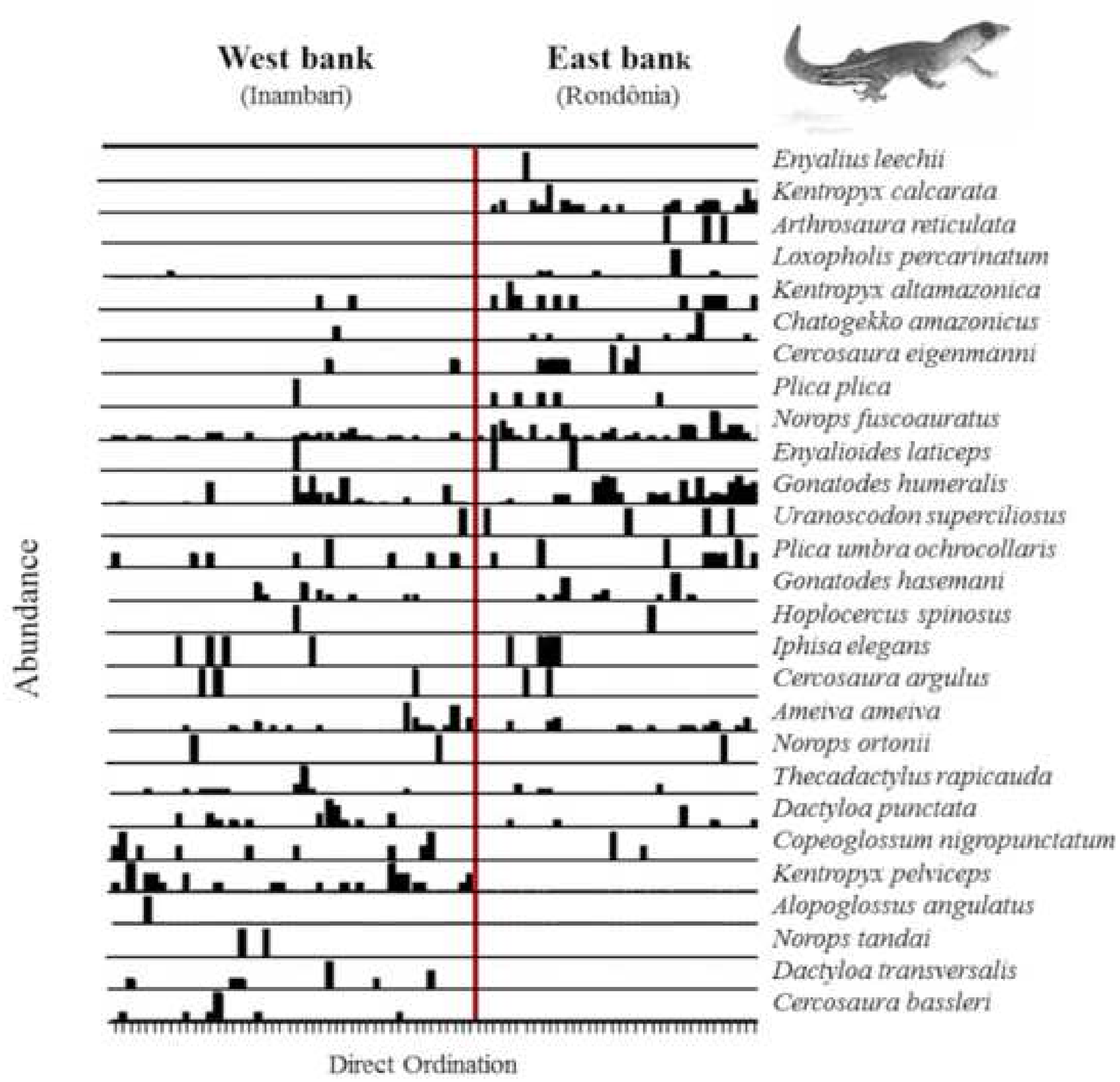
West bank
(Inambari)
East bank
(Rondônia)
Abundance
Direct Ordination
Enyalius leechii
Kentropyx calcarata
Arthrosaura reticulata
Loxopholis percarinatum
Kentropyx altamazonica
Chatogekko amazonicus
Cercosaura eigenmanni
Plica plica
Norops fuscoauratus
Enyalioides laticeps
Gonatodes humeralis
Uranoscodon superciliosus
Plica umbra ochrocollaris
Gonatodes hasemani
Hoplocercus spinosus
Iphisa elegans
Cercosaura argulus
Ameiva ameiva
Norops ortonii
Thecadactylus rapicauda
Dactyloa punctata
Copeoglossum nigropunctatum
Kentropyx pelviceps
Alopoglossus angulatus
Norops tandai
Dactyloa transversalis
Cercosaura bassleri

Figure

Click here to access/download;Figure;Fig 3.tif

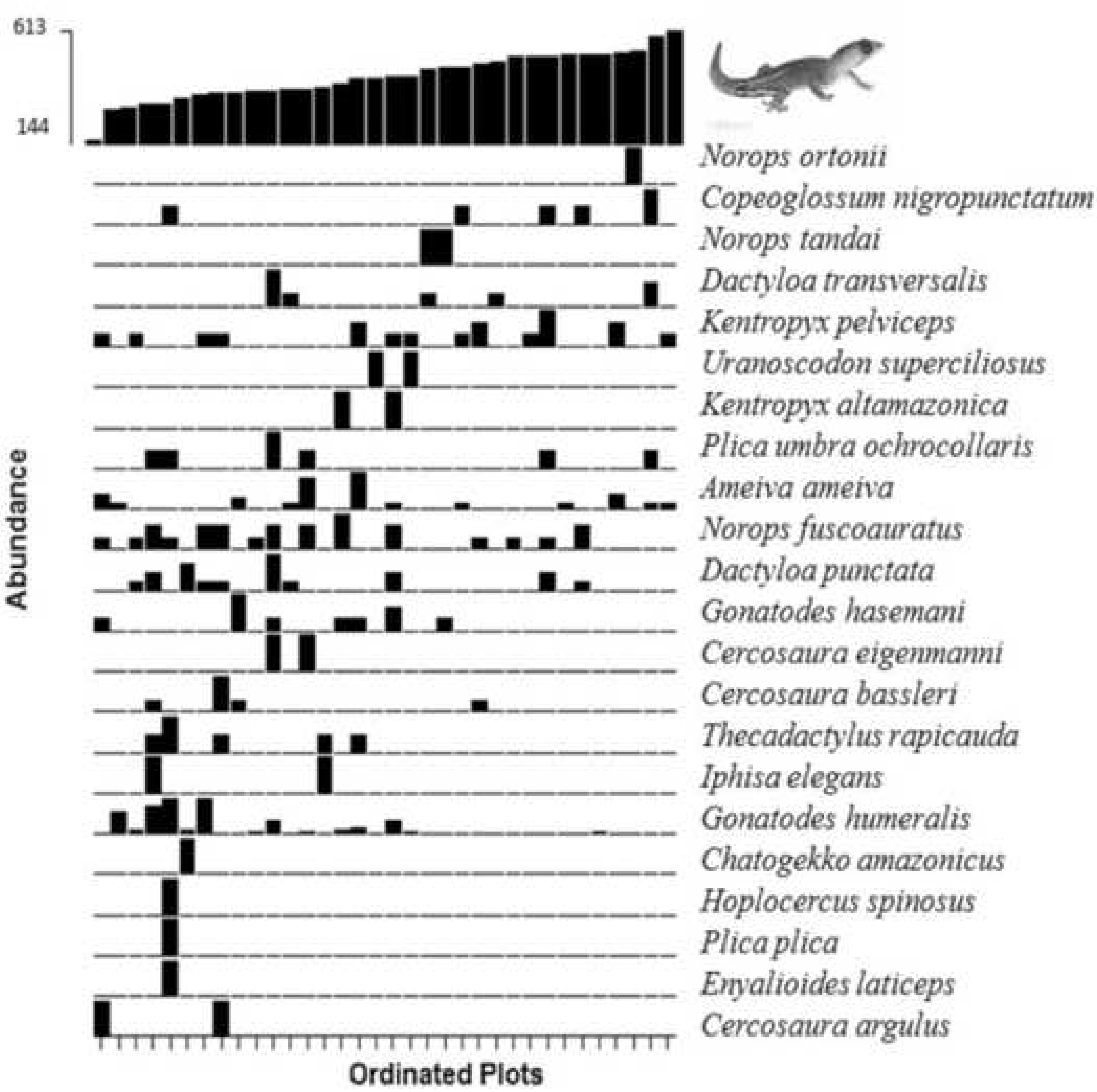

Figure
Click here to access/download;Figure;Fig 4.tif

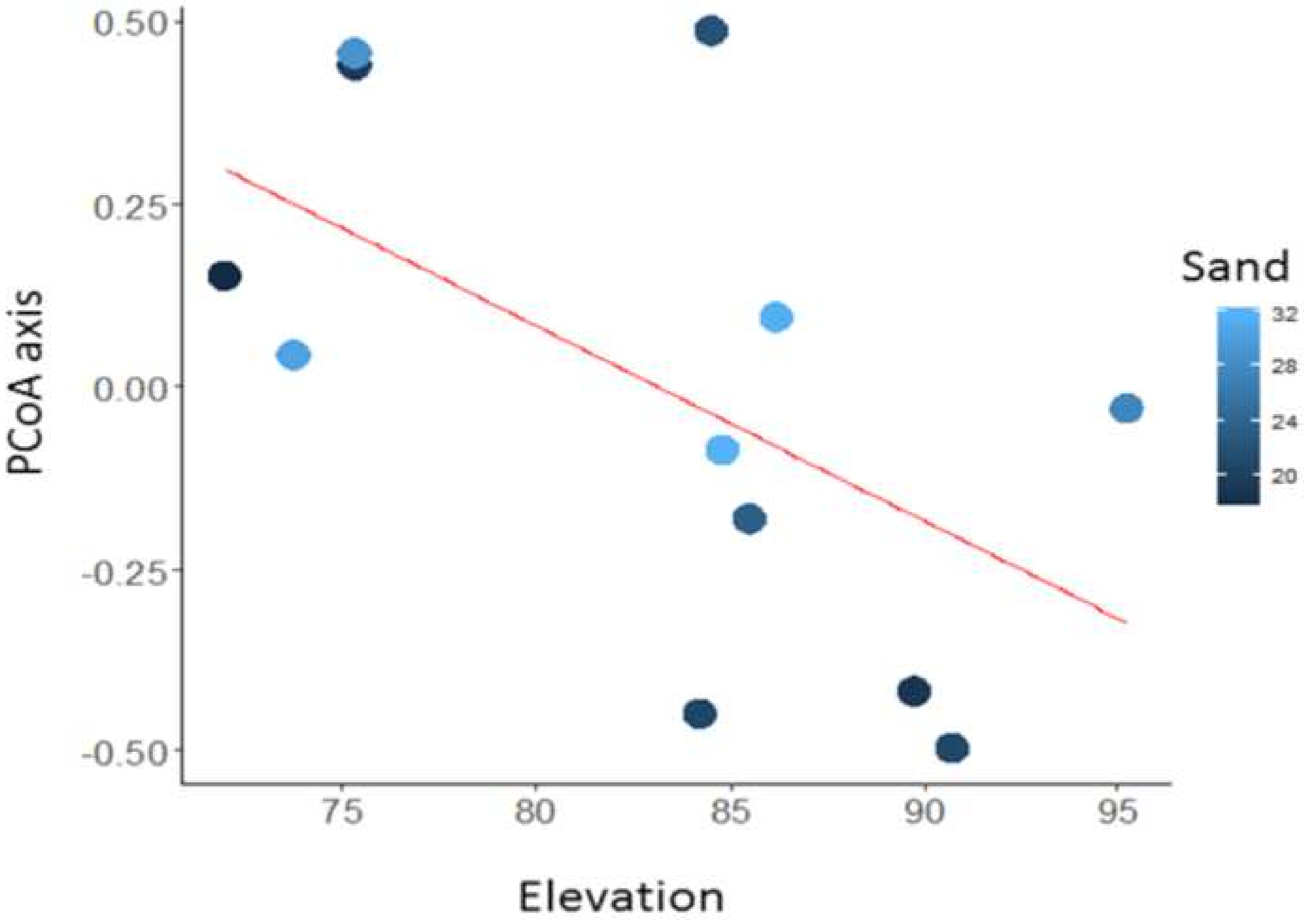

Click here to access/download;Figure;Fig 5.tif

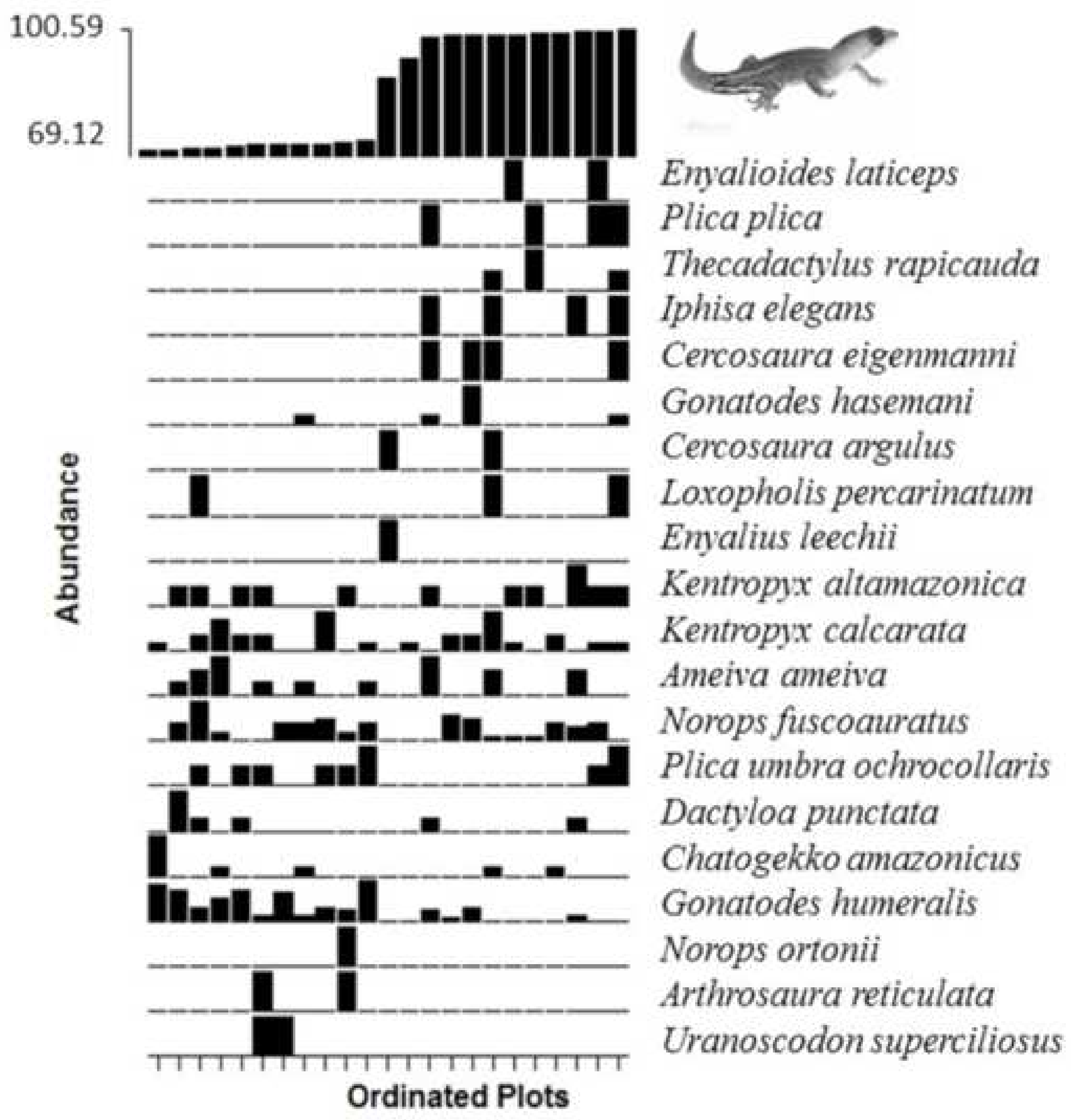

Figure

Click here to access/download;Figure;Fig 6.tif

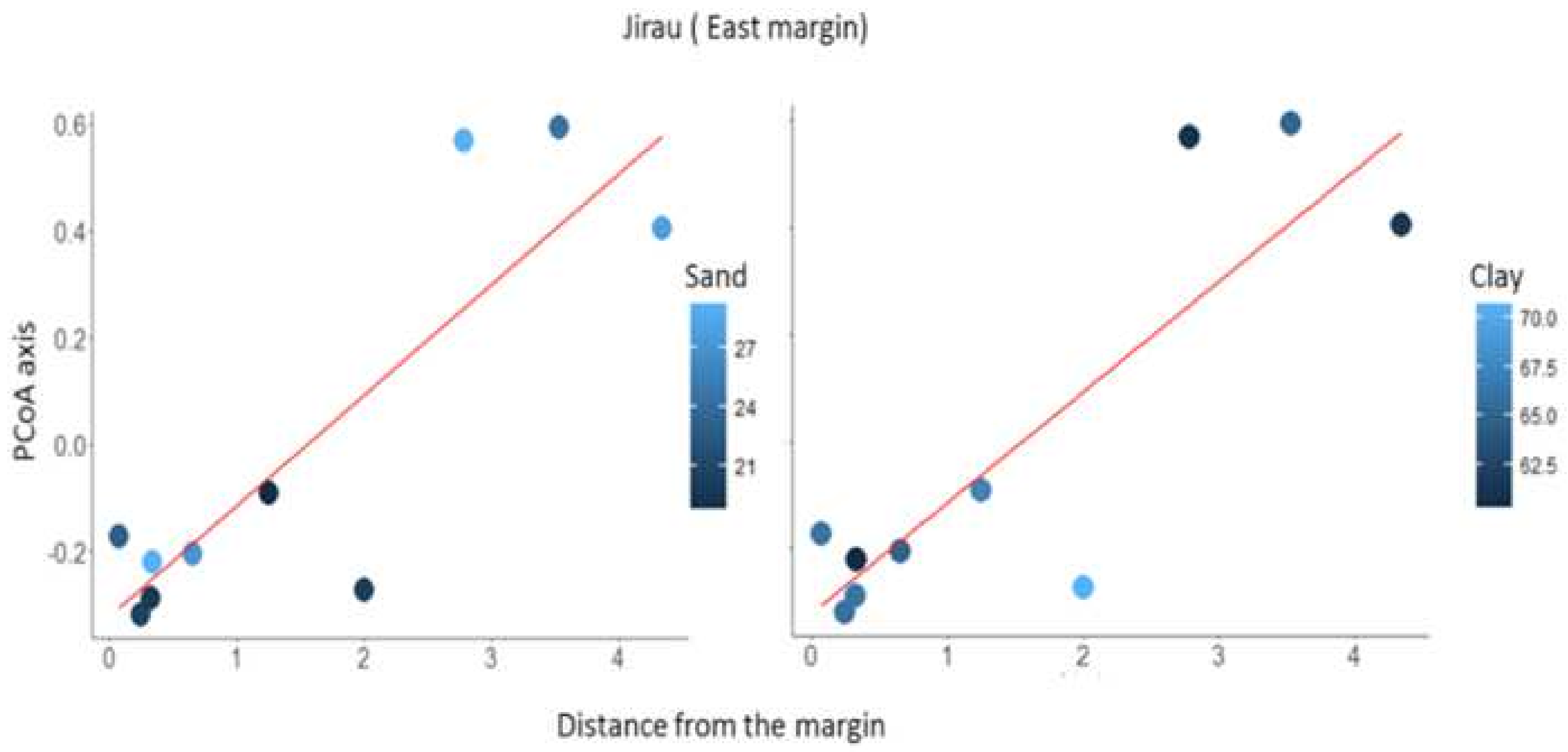

Figure.1

Click here to access/download;Figure;Fig 1.tif

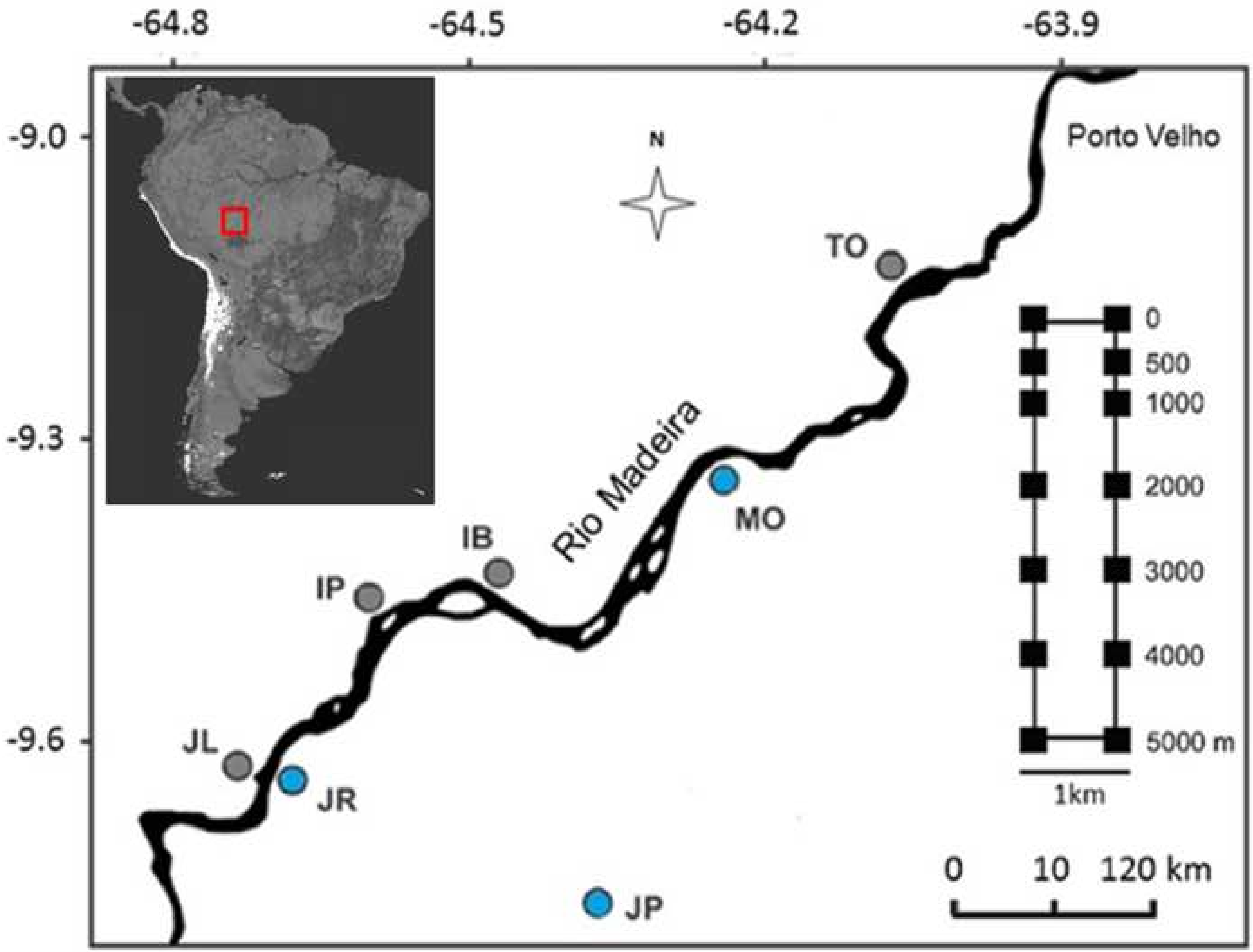

Click here to access/download
Supporting Information
S1 text.pdf

Click here to access/download
Supporting Information
S2 Fig 1.pdf

Click here to access/download
Supporting Information
S3 Fig 2.pdf

Hierarchical effects of historical and environmental factors on lizard assemblages in the upper Madeira river, Amazonian Brazil

Gabriela Marques Peixoto[1*¶], Rafael De Fraga[2¶], Maria C. Araújo[1¶], Igor Luis Kaefer[1,3¶], Albertina Pimentel Lima[1¶]

1 Pós-Graduação em Ecologia, Instituto Nacional de Pesquisas da Amazônia, Avenida André Araújo, Manaus, Amazonas, Brasil,

2 Pós-Graduação em Recursos Naturais Amazônicos, Universidade Federal do Oeste do Pará, Santarém, Pará, Brasil,

3 Instituto de Ciências Biológicas, Universidade Federal do Amazonas, Manaus, Amazonas, Brasil

***** Corresponding author

E-mail: gabriela.marquespd@gmail.com (GMP)

¶These authors contributed equally to this work.

Formatted: Portuguese (Brazil)

Field Code Changed

Formatted: Portuguese (Brazil)

Formatted: Portuguese (Brazil)

Abstract

Investigating the role of historical and ecological factors structuring assemblages is relevant to understand mechanisms and processes affecting biodiversity across heterogeneous habitats. Considering that community assembly often involves scale-dependent processes, different spatial scales may reveal distinct factors structuring assemblages. In this study we use lizard abundance data from 83 plots to investigate assemblage spatial structure at two distinct scales in southwestern Amazonian Brazil. At a regional scale, we test the general hypothesis that the Madeira River acts as a barrier to dispersal of some lizard species, which results in distinct assemblages between river banks. At a local scale, we test the hypothesis that assemblages are not evenly distributed across heterogeneous habitats but respond to a continuum of inadequate-to-optimal portions of environmental predictors ~~gradients~~. Our results show that regional lizard assemblages are structured by the upper Madeira River acting as barrier to 29.62% of the species sampled. This finding suggests species have been historically isolated at one of the river banks, although the strength of the barrier may depend on the regional shape of the river. At a local scale, different sets of environmental predictors~~gradients~~ affected assemblage composition between river banks or even within a river bank. These findings indicate that environmental filtering is a major cause of lizard assemblage spatial structure in the upper Madeira River, but predictor variables cannot be generalized over the study area. Based on a single study system we demonstrate that lizard assemblages along the forests near the banks of the upper Madeira River are not randomly structured but respond to multiple factors acting at different and hierarchical spatial scales.

Introduction

Investigating historical and ecological factors structuring assemblages may reveal patterns of biodiversity distribution across time and space [1,2]. However, defining mechanisms and processes that potentially affect assemblage structure is often highly dependent on the spatial scale applied [3-5]. Such dependence results from the fact that assemblage composition (e.g. taxonomic diversity) is influenced by complex hierarchical interactions among processes that operate at multiple spatio-temporal scales [6]. In highly heterogeneous habitats such as the Amazonian tropical rainforests the relative contribution of historical and ecological processes to assemblage structuring is poorly understood for many taxa, mainly because multi-scale ecological approaches depend on standardized sampling systems, which have been specifically designed~~design~~ for such purpose [e.g. 7-12]. Regarding lizards, poor knowledge on assemblage structure also results from lack of refined data on individual species distribution [13], despite few unpublished studies have shown assemblage spatial structure defined by environmental heterogeneity [e.g. 14-16].

Formatted: Not Highlight

At broad spatial scales (e.g. Amazon Basin), it has been suggested that many organisms are restrictedly distributed by their inability to cross large rivers. From the classic studies of Alfred R. Wallace on primate distribution across the Amazon Basin [e.g. 17], it has been known that the Amazon River and some of its main tributaries (e.g. Madeira, Negro) may be important biogeographic barriers to dispersal. Testing the Wallace´s hypothesis has revealed the riverine barrier as a major factor explaining limited distribution of plants [18], birds [19-22], frogs [23,12], primates [24,25] and spiny rats [26]. Additionally, studies have shown that gene flow reduced or blocked by a riverine barrier may cause genotypic and phenotypic divergence in Amazonia [27-29]. Specifically for lizards, riverine barriers may cause intraspecific genetic divergence [27], although they do not necessarily produce different

morphotypes [30]. Interspecifically, species distribution regionally limited to a single river bank may cause distinct assemblage compositions between banks [12,31].

At local scales, environmental predictors~~gradients~~ may affect species occurrence and abundance due to the filter effect of the spatial variation in habitat suitability [32,33]. In general, it is expected that habitat-specialist species find inadequate-to-optimum continuums of environmental conditions for survival and reproduction [34]. Environmental filtering has been found in Amazonia for plants [35,36], frogs [37,38], birds [39,40], snakes [41,42] and lizards [43,15]. For lizards~~the latter~~, local assemblages may differ due to variation in individual abundance or species turnover along gradients of distance from water courses [44,31], elevation [45], climate seasonality [46], and number of trees [47,43]. Additionally, lizard assemblages may be indirectly structured by species turnover along gradients of canopy openness affecting the availability of thermoregulation sites [48,49], understory-plant density affecting the availability of foraging sites for perching species [50], and clay content in the soil affecting plant composition and food availability [43].

Formatted: Not Highlight

Formatted: Not Highlight

Integrating multiple spatial scales is relevant to estimating simultaneous effects of historical and ecological factors on assemblage structure, especially in heterogeneous habitats such as rainforests in Amazonia [51]. However, designing a sampling system which is efficient to quantify assemblages and habitats at multiple scales may be challenging. The RAPELD [1] method (Brazilian acronym for rapid sampling plus long-term ecological research) has been shown to be efficient for this purpose in the region of the upper Madeira River [13], due to (i) the adequate distribution of plot sets (5 km^2 each) so that hypotheses based on the effects of historical factors on regional assemblages may be tested (e.g. riverine barriers), and (ii) the plots following altitudinal contours reduce within-plot environmental variation, which allows them to be assumed as environmental units to test hypotheses based on environmental filtering [1]. The rationale behind testing such hypotheses in southwestern

Amazonia is that the Madeira River has been recognized as a barrier to dispersal of Squamata reptiles, which causes species turnover along a longitudinal gradient [52], and the region covers two endemism zones (Rondônia e Inambari) that are distinct regarding geological history and environmental heterogeneity [53].

In this study we use plot-based lizard abundance data from the upper Madeira River (southwestern Amazonian Brazil) to investigate patterns of assemblage structure at two distinct spatial scales. At a regional scale, we test the hypothesis that lizard assemblages differ between the river banks. We expect differences in species composition and abundance as a consequence of the Madeira River historically limiting lizard dispersal. At local scale, we test the hypothesis that environmental heterogeneity causes species turnover, because species are absent or occur at low densities in suboptimal portions of environmental predictors~~gradients~~. Specifically, we quantify the filtering effects on lizard abundance driven by gradients of number of trees, soil nutrient composition, shrub density, elevation, clay and sand content in the soil, and distance from the river bank. We expect that analyzing assemblages from two distinct perspectives will provide us with deep insights into factors that cause and maintain biodiversity at megadiverse regions such as the upper Madeira River.

Materials and methods

Study area

The study area is located near the banks of the upper Madeira River (coordinates of the centroid 08 ° 48004.0 "S; 63 ° 56059.8" W), an important tributary of the Amazon River classified as a white and muddy river with a total length of 1,459 km. The upper Madeira River extends from the outskirts of Porto Velho (state of Rondônia) to about 600 km upstream, in southwestern Amazonia. We also sampled plots close to the Jaci Paraná river, a tributary on the east bank of the upper Madeira river (Fig. 1).

Formatted: English (United States)

Formatted: English (United States)

~~The study area is located near the banks of the upper Madeira River (centroid coordinates 08°48004.0″ S; 63°56059.8″ W), from the outskirts of Porto Velho (Rondônia state) to about 600 km upriver, in southwestern Amazonian Brazil. We also surveyed plots near the Jaci Paraná River, a tributary on the east bank of the upper Madeira River (Fig 1).~~

Formatted: English (United States)

Fig 1. Location of the upper Madeira River, state of Rondônia, Brazil. Sampling 5 km^2 modules (circles) near the banks. Gray circles show modules in the Inambari endemism zone, blue circles are modules in the Rondônia endemism zone [according to 20]. The acronyms summarize sampling modules´ local names: TO = Teotônio, IB = Ilha dos Búfalos, IP = Ilha das Pedras, JL = East Jirau, JR = West Jirau, JP = Jaci-Paraná, MO = Morrinhos. In detail on the left side, the standard configuration of each module, with 14 plots (squares), 250 m-long each, distributed along a gradient of distance from the river bank (0–5,000 m).

In this study we quantified environmental heterogeneity as continuous gradients that may be broadly classified for descriptive purposes in three main habitat types. They mainly differ in canopy height, soil texture, and understory-plant density and species composition [following 54]. In the upland (terra-firme) forests habitats are never flooded by overflowing large rivers, the canopy is 30 m high, and the understory-plant density and clay content in the soil often depends on elevation [55]. The várzea forests are seasonally flooded by overflowing sediment-rich rivers, which produces nutrient-rich soils that are water-saturated for long periods. The canopy is 20 m high, and the understory is rich in bromeliads. The campinaranas are patches of palm tree-rich forests growing on a white-sand soil, which is highly drained and nutrient-poor [54].

The climate of the study area is tropical humid, with annual average temperature at 25.5 °C and average precipitation at 2,287 mm. Precipitation is distributed throughout the year

in well-marked dry (May to September) and rainy (October to April) seasons. During the dry season, small streams can dry completely [56].

Sampling design

We collected lizard abundance data in seven 5 km^2 RAPELD sampling sites (hereinafter modules), that were installed perpendicularly to the river bank. RAPELD [1] is a modification of the Gentry´s sampling method based on 1-ha plots [57], with the main difference being that the RAPELD plot central lines follow the altitudinal curves to reduce environmental variation within plots (PPBio - http://ppbio.inpa.gov.br). We sampled three modules on the east bank of the Madeira River (East-Jirau, Jaci-Paraná and Morrinhos), and four modules on the west bank (West-Jirau, Ilha das Pedras, Ilha dos Búfalos and Teotônio). The average distance between neighboring modules was 120 km. Each RAPELD module was composed of two 5-km long parallel trails, separated by 1 km. We surveyed seven 250 m plots (20 m wide) on each trail, totaling 98 plots (14 plots in each of the seven modules). The plots were distributed along a gradient of distance from the river bank, at 0, 500, 1000, 2000, 3000, 4000 and 5000 m.

We were not able to find lizards in 15 plots, and the excess of zeros in the dataset prevented us to reliably estimate pairwise distances among plots to summarize assemblage composition (see Data analysis). Therefore, we excluded zero-valued plots and our analyzes are based on 83 plots.

Formatted: Not Highlight

Sampling effort

We sampled each plot in four different periods (24 February to 26 April 2010, July 30 to August 19 2010, November 5 to 26 2010, and January 13 to February 4 2011) to cover large portions of the regional variation in temperature and precipitation along a year. We used species' maximum abundance values per plot in the analyzes.

We found lizards using active visual search, with two simultaneous observers positioned 10 m apart. In addition, we supplemented the sampling effort by sweeping the leaf litter and removing debris in a 2 m strip following the center line of the plot. This approach was particularly useful to increase the efficiency of sampling fossorial and leaf-litter species (e.g. Alopoglossidae, Gymnophthalmidae). The searching time in each plot varied between 40 and 60 minutes.

Environmental variables

We measured eight environmental predictors in each plot in order to quantify spatial heterogeneity in habitat suitability.~~We measured eight environmental gradients in each plot, attempting to quantify spatial heterogeneity in habitat quality.~~ We quantified vegetation structure by measuring (i) number of trees and (ii) shrub density. Those predictors~~gradients~~ potentially affect squamates abundance by influencing availability of foraging, resting and thermoregulation sites [58-60]. We also measured edaphic gradients related to soil texture, fertility, and flat-level deviation, which are (iii) clay content, (iv) sand content, (v) nutrient composition (Soil pH, Calcium, Magnesium, Potassium, Zinc and exchangeable Aluminum), (vi) elevation, and (vii) terrain declivity. Those variables potentially affect lizard abundance by causing variation in the overall primary production [61].~~,~~ and availability of invertebrate prey [62]. Additionally, we measured (viii) distance from the river bank, because it has been found as a major factor structuring plant [36] and animal [31,38,39,41] assemblages in Amazonia. The methods used to measure each predictor ~~gradient~~ are described in detail in Appendix 1.

Formatted: Not Highlight

Data analysis

To quantify assemblage composition, we applied the Bray-Curtis index to estimate pairwise distances in species abundance among plots. We reduced dimensionalities using

Principal Coordinate Analysis (PCoA) and represented assemblage composition by the first one or two axes produced (see below).

At regional scale (riverine barrier effects) we modeled the PCoA using all data (83 plots). The two first axes captured 30% (PCoA 1 = 16%. PCoA 2 = 14%) of the original variance in species abundance, and we used them to represent assemblage composition. To assess assemblage structuring, we used Multivariate Analysis of Variance MANOVA to test differences in assemblage composition (PCoA axes 1 and 2) between the river banks. We implemented a MANOVA using the vegan [63] R-package [64].

Analyzes at regional scale revealed two distinct lizard assemblages between the river banks (see Results). In addition, preliminary analyzes at local scale revealed that in two modules (Ilha das Pedras and East Jirau) environmental predictorsgradients may affect assemblage composition in opposite directions compared to the other modules (S2 and S3 Fig). These findings suggested that the banks of the Madeira River and some of the sampling modules within a river bank are distinct environmental units, which contain distinct spatial structures of lizard assemblage composition. Therefore, to assess assemblage structure at local scale we modeled four distinct PCoA ordinations, using data from (i) the west bank, except for the module Ilha das Pedras (37 plots), which captured 86% of the original variance (PCoA 1 = 0.50, PCoA 2 = 0.36); (ii) the east bank, except for the module East Jirau (23 plots), which captured 45% of the original variance (PCoA 1 = 0.30, PCoA 2 = 0.15); (iii) the module Ilha das Pedras (12 plots), which captured 45% of the original variance (PCoA 1 = 0.32, PCoA 2 = 0.13); and (iv) the module East Jirau (11 plots), which captured 85% of the original variance (PCoA 1 = 0.49, PCoA 2 = 0.36).

The environmental predictors gradients measured are expressed in different units and therefore in different orders of magnitude, so we transformed them using the “scale” function of the vegan R-package. This function subtracts mean values from each variable and scales

centralized variables by dividing them by their standard deviation [63]. We used Mixed Linear Models to test the effects of scaled environmental predictors~~gradients~~ on assemblage composition based on data from multiple sampling modules. By using this method, we were able to include sampling modules as random effects to minimize potential abrupt differences in environmental predictors~~gradients~~ and lizard assemblages among the modules analyzed in a same model [65]. We set up two different groups of mixed models, according to the assemblage compositions summarized by PCoA for the west and east banks of the Madeira River. Each group was composed of as many models as necessary to test all possible combinations of environmental predictors~~gradients~~, except for those predictorsthat were highly correlated. For instance, clay and sand content in the soil were not used in a same model because they were highly correlated on both river banks (r ≥ 0.93). In addition, elevation was correlated with terrain declivity on both river banks (r ≥ 0.78) and soil-nutrient composition on the east bank (r = 0.66). The cut-off point was r = 0.51.

For the two modules that were analyzed separately (Ilha das Pedras and East Jirau), it was not necessary to control random effects of sampling sites, so we tested the effects of environmental predictors~~gradients~~ on the assemblage composition using multiple linear regression models. We tested models with assemblage composition (PCoA 1) as dependent variable, and all possible combinations of uncorrelated environmental predictors~~gradients~~ as independent variables.

To select the most parsimonious mixed-effects and multiple-regression models we ranked all the models by the corrected Akaike´s Information Criterion (AICc) [66]. We refined the model selection by penalizing nested models assuming ΔAICc < 2 as a cut-off point. All selected models were validated by normal distribution of residuals (Shapiro-Wilk W > 0.95, P > 0.05 in all cases).

Formatted: Not Highlight

Formatted: Not Highlight

Formatted: Not Highlight

Formatted: Not Highlight

To select the most parsimonious mixed-effects and multiple regression models we ranked all the models by the Akaike´s Information Criterion corrected for few parameters [66]. We refined the model selection by penalizing nested models assuming ΔAICc < 2 as a cut-off point. All selected models were validated by normal distribution of residuals (Shapiro-Wilk W > 0.95, P > 0.05 in all cases).

For visually checking the distribution of lizard abundance values per species along river banks and environmental predictorgradients (only those that significantly affected assemblage composition) we plotted ordinated sampling plots. These graphs will be used in this study for assessing how spread the distributions of abundance values are over the river banks and the environmental heterogeneity measured.

RESULTS

We found 27 lizard species, which are classified in 18 genera and 10 families. The most frequently found species were *Norops fuscoarautus* (Dactyloidae), *Gonatodes humeralis* (Sphaerodactylidae), and *Ameiva ameiva* (Teiidae), which occurred in both banks of the Madeira River, in 55, 49 and 30% of the plots respectively. Contrarily, *Alopoglossus angulatus* (Alopoglossidae) and *Enyalius leechii* (Leiosauridae) were found in one single plot (Table 1).

Table 1. List of lizard species sampled in the upper Madeira River, Brazil. N = total abundance per species, East and West = Madeira river banks filled with presence (1) and absence (0) data.

Family/Species	N	East	West
**Dactyloidae**			
Norops fuscoauratus (D'Orbigny, 1847)	103	1	1
Norops tandai (Wagler, 1830)	2	0	1

Norops ortonii (Cope, 1869)	2	1	1
Dactyloa punctata (Daudin, 1802)	27	1	1
Dactyloa transversalis (Dumeril, 1851)	9	0	1
**Alopoglossidae**			
Alopoglossus angulatus (Linnaeus, 1758)			
**Gymnophthalmidae**	2	0	1
Arthrosaura reticulata (O'Shaughnessy, 1881)	5	1	0
Cercosaura argulus (Peters, 1863)	5	1	1
Cercosaura eigenmanni (Griffin, 1917)	11	1	1
Cercosaura bassleri (Ruibal, 1952)	8	0	1
Iphisa elegans (Gray, 1851)	8	1	1
Loxopholis percarinatum (Muller, 1923)	10	1	1
**Hoplocercidae**			
Enyalioides laticeps (Guichenot, 1855)	3	1	1
Hoplocercus spinosus (Fitzinger, 1843)	2	1	1
**Leiosauridae**			
Enyalius leechii (Boulenger,1885)	2	1	0
**Scincidae**			
Copeoglossum nigropunctatum (Spix, 1825)	14	1	1
**Phyllodactylidae**			
Thecadactylus rapicauda (Houttuyn, 1782)	21	1	1
**Sphaerodactylidae**			
Chatogekko amazonicus (Andersson, 1918)	12	1	1
Gonatodes hasemani (Griffin, 1917)	29	1	1
Gonatodes humeralis (Guichenot, 1855)	432	1	1
**Teiidae**			
Kentropyx altamazonica (Cope, 1876)	14	1	1
Kentropyx calcarata (Spix, 182)	37	1	0
Kentropyx pelviceps (Cope, 1868)	29	0	1
Ameiva ameiva (Linnaeus, 1758)	48	1	1
**Tropiduridae**			
Plica plica (Linnaeus, 1758)	7	1	1
Plica umbra ochrocollaris (Spix, 1825)	21	1	1
Uranoscodon superciliosus (Linnaeus, 758)	5	1	1
**Total**	**868**		

Regional assemblage structuring - Madeira River as a biogeographic barrier

We found 19 species on both banks of the Madeira River, which is equivalent to 70.37% of the total diversity sampled. This finding suggests that most of the species sampled

are widely distributed throughout the study area. However, for several of the species found on both sides of the river (e.g. *Loxopholis percarinatum*, *Kentropyx altamazonica*, *Cercosaura eigenmanni*, *Plica plica*, *Uranoscodon superciliosus*, *Copeoglossum nigropunctatum*), plot-related frequency and abundance were not even between the river banks (Fig 2). Additionally, five species (18.52%) were restricted to the west bank – *Alopoglossus angulatus*, *Norops tandai*, *Dactyloa transversalis*, *Cercosaura bassleri* and *Kentropyx pelviceps*, and three species (11.11%) were restricted to the east bank –*Arthrosaura reticulata*, *Kentropyx calcarata* ~~e~~*and* *Enyalius leechii*. These findings suggest two distinct assemblage compositions delimited by the Madeira River, which is strongly supported by differences in the PCoA scores (based on 83 plots) between the river banks (MANOVA Pillai Trace = 0.315, $F_{1–81}$ = 18.40, P <0.001).

Formatted: Not Highlight

Fig 2. Plots ordinated according to their position of the upper Madeira River (west and east). The heights of the black rectangles are relative to species abundance values.

Local assemblage structuring – the role of environmental predictors ~~gradients~~

Formatted: Font: Bold

On the west bank of the Madeira River (except for the module Ilha das Pedras) three mixed-effects models were selected by ΔAICc < 2 (Table 2). All the selected models consistently returned number of trees as a major gradient affecting assemblage composition (P < 0.001 in all cases). Despite some species occupied large portions of the gradient of number of trees (e.g. *Ameiva ameiva*, *Norops fuscoauratus*), species absence or low abundance in specific intervals between 144 and 613 trees caused species turnover (Fig 3). According to the models selected, assemblage composition was not affected by elevation (P = 0.76), and soil-content of sand (P = 0.84) or clay (P = 0.91).

Table 2. Summary of the results returned by Linear Mixed-Effects Models. The models were set up using data from the west (Teotônio, Ilha dos Búfalos and West Jirau) and east (Morrinhos and Jaci-Paraná) banks of the upper Madeira River. The models were selected by AICc Δ < 2. Shapiro-Wilk tests were applied on the residuals from each model to test normality. Bolded p-values show cases in which the null hypothesis was rejected.

Formatted: Not Highlight

**Margins**	**Fixed effects**	**AICc**	**Weight**	***df***	***t***	***p***	**Total variance**	**Shapiro-Wilk**
**West**	Number of Trees and Elevation	12.89	0.314	Intercept:2.18	-6.92	<**0.001**	54%	P=0.109
				Trees:3.01	18.8	<**0.001**		
				Elevation:1.43	-0.31	0.76		
	Sand and Number of Trees	12.88	0.309	Intercept:3.39	-14.69	**0.001**	69%	P=0.066
				Sand:1.86	-0.19	0.84		
				Trees:3.29	18.8	<**0.001**		
	Clay and Number of Trees	12.88	0.305	Intercept:1.00	-9.07	<**0.001**	76%	P=0.153
				Clay:9.76	0.10	0.91		
				Trees:3.27	18.88	<**0.001**		
**East**	Elevation and Margin distance	2.0	0.412	Intercept:2.30	6.37	<**0.001**	71%	P=0.782
				Elevation:2.30	-6.27	<**0.001**		
				Margim:2.30	1.72	0.09		

	Number of Trees and Elevation	2.0	0.400	Intercept:2.30	5.92	<**0.001**	72%	P=0.413
				Trees:2.10	18.8	0.10		
				Elevation:2.30	-0.31	<**0.001**		

Fig. 3. Plots ordinated according to their position relative the number of trees measured in the west bank of the upper Madeira River, state of Rondônia, Brazil. The heights of the black rectangles depict the relative species abundances.

Three multiple-regression models were selected for Ilha das Pedras sampling module (Table 3), ~~**Fig. 3. Plots ordinated according to their position relative the number of trees.** Measured in the west bank of the upper Madeira River, state of Rondônia, Brazil. The heights of the black rectangles are relative to species abundance values.~~

~~For the Ilha das Pedras module three multiple-regression models were selected (Table 3),~~ all of them containing elevation as an independent variable. This predictors~~gradient~~ significantly affected assemblage composition according to a model constructed with soil sand content as an additional independent variable (P = 0.05) (Fig 4). However, the effects of elevation on the assemblage composition were marginally significant in models containing number of trees (P = 0.06) and soil clay content (P = 0.07) as independent variables.

Table 3. **Summary of the results returned by Linear Mixed-Effects Models.** The models were set up using data from the Ilha das Pedras (west river bank) and East Jirau (east river bank) modules to~~for~~ test the effects of environmental predictors~~gradients~~ on lizard assemblage composition. The models were selected by AICc Δ < 2. Shapiro-Wilk tests were applied on the residuals from each model to test normality. Bolded p-values show cases in which the null hypothesis was rejected.

Margins	Variables	AICc	Weight	Std. error	*t*	P	*F*	r2
West	Number of Trees and Elevation	15.7	0.31	Intercept:8.76	0.00	1.00	2.746	0.37
				Trees:9.19	0.90	0.39		
				Elevation:9.19	-2.06	0.06		
	Sand and Elevation	15.8	0.29	Intercept:8.81	0.00	1.00	2.66	0.37
				Sand:9.21	0.85	0.42		
				Elevation:9.21	-2.18	**0.05**		
	Clay and Elevation	16.2	0.25	Intercept: 1.21	0.1	1.10	2.45	0.35
				Clay:6.12	-0.64	0.53		
				Elevation:1.88	-1.98	0.07		
East	Clay and Distance from the margin	6.5	0.655	Intercept: 5.69	0.00	1.00	15.42	0.81
				Clay: -1.05	-1.69	0.13		
				Margim:2.89	4.63	<**0.001**		
	Sand and Distance from the margin	7.9	0.367	Intercept: 2.30	5.92	<**0.001**	13.07	0.78
				Sand:8.81	1.28	0. 24		
				Margim:2.85	4.15	<**0.001**		

Fig. 4. Partials from a multiple linear model for the Ilha das Pedras module. Model for the effects from the elevation and sand contents in the soil on lizard assemblage composition.

Assemblage composition was summarized by the first axis of an Analysis of Principal Coordinates based on abundance data of the upper Madeira River, state of Rondônia, Brazil. The shades of blue show values of sand content in the soil.

On the east river bank (except for the East Jirau module) two models were selected as most parsimonious. Both models consistently showed strong effects of elevation on assemblage composition (P < 0.001 in both cases). This finding suggests species turnover along an elevational gradient of 69.12–100.59 m (Fig 5). According to the same models, distance from the river bank (P = 0.09) and number of trees (P = 0.1) did not affect assemblage composition.

Fig 5. Plots ordinated according to their position relative to a gradient of elevation (meters above the sea level) in the east bank of the upper Madeira River, state of Rondônia, Brazil. The heights of the black rectangles depict the relative species abundances.

~~**Fig 5. Plots ordinated according to their position relative to a gradient of elevation.** Calculated for the meters above the sea level in the east bank of the upper Madeira River, state of Rondônia, Brazil. The heights of the black rectangles are relative to species abundance values.~~

Two multiple-regression models were selected for the East Jirau module. Both models consistently returned distance from the river bank (Fig 6) as a relevant gradient affecting assemblage composition (P < 0.001 in both cases). Soil-content of sand (P = 0.24) and clay (P = 0.13) did not affect assemblage composition.

Fig 6. Partials from a multiple linear model ~~used~~ from the East-Jirau module. The effects of distance from the river bank, sand and clay contents in the soil on lizard assemblage

composition. Assemblage composition was summarized by the first axis of an Analysis of Principal Coordinates based on abundance data from the East Jirau sampling module, located on the east bank of the upper Madeira River, state of Rondônia, Brazil. The shades of blue show values of sand and clay contents in the soil.

Discussion

At regional scale, we found that lizard assemblages are spatially structured by differences in assemblage composition between river banks. This finding is consistent with large Amazonian rivers acting as dispersal barriers for several organisms, which have caused different species subsets composed of plants [18], birds [19,21], primates [24] and diurnal frogs [12]. At local scale, we showed that lizard assemblages are spatially structured by species turnover along environmental predictors~~gradients~~. However, a set of environmental predictors~~gradients~~ cannot be assumed as generalized predictors among sampling sites. Our overall results are broadly consistent with frog assemblages sampled in the same plots [12], which suggests multi-taxa ecological patterns. We relied on a single dataset to provide understanding about assemblage structure based on interacting historical and ecological processes. Therefore, we highlight the relevance of investigating multi-scale assemblage structuring for ecology and conservation decision making.

In the upper Madeira River, assemblage divergence between river banks has been attributed to historical processes regionally reducing species dispersal [12]. Approximately half of the species present in the assemblage of diurnal frogs (13 species) of that region were restricted to one of the river banks [12]. ~~In the upper Madeira River, assemblage divergence between river banks has been attributed to historical processes regionally reducing species dispersal [12]. Approximately 50% of the species composing a frog assemblage (13 species) were absent in one of the river banks.~~ The smaller proportion of regionally isolated lizard

species (29.63%) is reasonably explained by the lower dispersal capacity of small and site-attached frogs compared with most lizards. Nonetheless, we investigated aseemblages in which about 30% of the sampled species were isolated by the river, and another 30% of the species occurred at low relative frequency or abundance at one of the river banks. This was a sufficiently adequate scenario to assume the river as a historical factor segregating assemblages between the river banks. We highlight that most of regionally isolated species in our sample are widely distributed throughout Amazonia outside our study area [13]. Such inconsistency may be explained by the strength of the river as a dispersal barrier varying along the river course, or even being nullified in response to meandering shapes [67- 69,30]. Additionally, the barriers may be seasonal, because bridges for stepping-stone dispersal may be revealed during the dry season, which allows gene flow between river banks [70]. Therefore, our results for assemblage structure at regional scale should not be extrapolated to unsampled stretches of the Madeira River or other Amazonian rivers, because lizards probably have found multiple dispersal routes through evolutionary time [27].

The isolation of species on one of the river banks may be related to the geomorphological heterogeneity of the Madeira River across our study area. The Madeira river flows over an incisive fluvial valley, with predominantly crystalline and a geologically ancient basement (ca. 16 Ma). The morphodynamical development was mainly influenced by the geomorphological and climatic changes resulting from the Andean Orogeny in the Cenozoic [71], which have produced a relatively stable course along recent geological times [72]. Such stability in the shape of the river course has prevented meandering across most of the study area, which could facilitate for species to cross the river [73]. Exceptionally, the modules located further upstream (East and West Jirau) have rocky outcrops that are exposed in the middle of the river course during the dry season, which can act as bridges for stepping-stone dispersal (field observation). Although lizard species used alternative dispersal routes

to widespread their distribution throughout Amazonia, our study showed that they were regionally prevented from colonizing or maintaining populations on both banks of the upper Madeira River. One could argue that our results of a river-barrier effect are biased due to the low detection probability of lizards, which resulted in false absence of species [74,75]. However, we think that a possible sampling bias was mitigated by the large sampling effort associated to the combination of different sampling methods employed in this study.

Formatted: Font:

Besides affecting assemblage composition, the effect of rivers as barriers can also be observed at the intraspecific level in different biogeographic domains, resulting in genetic and morphological divergence among lizard populations due to restriction to gene flow [30,76,77]. Studies on the genetic and phenotypic differentiation of populations of a same species on opposite banks of the Madeira River should be performed as they might help to understand the initial steps of allopatric speciation in Amazonian lizards.

Although lizard species used alternative dispersal routes to widespread their distribution throughout Amazonia, our study showed that they were regionally prevented from colonizing or maintaining populations on both banks of the upper Madeira River.

At local scale, lizard assemblages were spatially structured by environmental filtering causing non-random assemblage composition. Environmental conditions selected species that were unable to survive and maintain viable conditions in given sampling plots [784]. Despite we sampled species that are generalist in relation to the environmental predictorsgradients measured (e.g. *Ameiva ameiva*, *Norops fuscoauratus*), species for which distributions were restricted to narrow regions of gradients (e.g. *Cercosaura argulus*, *Norops ortonii*, *Uranoscodon superciliosus*) caused species turnover across sampling plots. Species turnover mediated by environmental filtering is a major factor structuring local assemblages in Amazonia [e.g. 41,36,39], and in the upper Madeira River it has efficiently explained

Formatted: Not Highlight

assemblage structure in bats [79~~5~~], frogs [12] and snakes [80~~76~~]. However, we cannot generalize a single environmental dataset as a predictor for assemblage composition in all plots. Environmental predictors for assemblage composition differed between the river banks or even within a river bank. This finding suggests that the scale at which lizard assemblages respond to environmental heterogeneity may be more refined than the classification of the Madeira river banks as distinct endemism zones [81~~77~~,20].

Number of trees was a major factor causing species turnover in the west bank of the Madeira River. This gradient ranged from 144 to 613 trees, which shows that the vegetation structure is quite heterogeneous throughout our study area. Heterogeneity in vegetation structure affects species occurrence and abundance due to variation in the availability of foraging, nesting, resting, and thermoregulating sites [58,60]. Additionally, tree cover may directly affect food availability, protection against predators, light intensity, temperature, humidity and wind speed [59,60]. The evidence for assemblage structuring along a gradient of number of trees is of concern from a conservation point of view, because our study area has been intensely deforested by the agribusiness and large hydroelectric plants [82~~78~~]. It is widely expected that species dependent on high levels of tree cover (e.g. *Norops tandai*, *Norops ortonii*, *Dactyloa transversalis*) will either be locally extinct or migrate to more suitable habitats. ~~Future studies should focus on investigating species turnover at time scales.~~

We found species turnover along an elevational gradient, although this finding was most evident on the east bank of the Madeira River. On the east bank the plots were installed on the depression of the Ji-Paraná river, which generated elevation values below 30 m. Low elevation is often related to outcropping of groundwater and high drainage density [83~~79~~,71], which favors the occurrence of habitat-specific species for high humidity. For instance, *Arthrosaura reticulata* and *Uranoscodon superciliosus* typically occupy humid low areas [84~~0~~,85~~1~~], and in this study those species were found only on the east bank of the Madeira

Formatted: Not Highlight

Formatted: Not Highlight

River. Additionally, elevation indirectly influences assemblage composition because it affects water availability and soil fertility [86~~2~~-87~~4~~], and therefore the overall structure of available habitats [88,89~~5~~]. Extreme variation in elevation may cause behavioral and morphological differentiation in ~~Andean~~ lizards [90~~86~~]. In this study we showed that even subtle variation in elevation (24 to 128 m) may be sufficient for species to be locally filtered. A similar finding was observed using frog assemblage data from the Guiana Shield [37].

The gradient of distance from the river caused species turnover in the East Jirau module. Although habitats may be classified in riparian and non-riparian zones [91~~87~~], gradients of distance from water courses carry multiple continuous interacting variables of microclimate, nutrient availability, vegetation cover and edaphic structure. Habitats continuously changing along gradients of distance from streams (< 12 m wide) have caused species turnover structuring plant [36], frog [38], bird [39] and snake [41] assemblages. We have shown a similar pattern using lizard abundance data, with the main difference being that the gradient we measured refers to the distance from the bank of one of the major tributaries of the Amazon River. However, no significant effect of distance from the river on assemblage composition was observed using data from the other modules~~However, no significant effect of distance from the river on assemblage composition was returned using data from the other modules~~. This finding suggests that assemblages diverging between riparian and non-riparian zones should not be generalized in relation to gigantic rivers, or assemblage segregation should occur at distances that are greater than 5 km away from the river bank.

Some of the results found may be associated to environmental variables that were not explicitly measured in this study. For example, *Hoplocercus spinosus* (Hoplocercidae) occurred on both banks of the Madeira River but occurrence was restricted to plots with rocky outcrops. Such condition was only found in the westernmost sampling modules of the study area (East and West Jirau), where the species finds optimal availability of thermoregulation

Formatted: Not Highlight

Formatted: Not Highlight

and refuge sites [91~~88~~]. This finding reflects relationships between species and habitats that are dependent of biological traits affecting survival [92~~89~~,93~~0~~] and dispersal capacity [94~~1~~,95~~2~~], such as body size, diet [96~~3~~], specificity level in habitat use [89~~1~~], reproductive [49] and foraging mode [97~~76~~]. Therefore, although patterns of assemblage structure are usually described based on dissimilarities among plots regarding subsets of cooccurring species, they may be determined by ecological requirements of individual species.

We have shown that lizard assemblages in the upper Madeira River are structured by scale-dependent hierarchical factors. Historical processes related to the Andes uplift [98~~4~~] have isolated regional assemblages between the river banks, and have also generated distinct habitat patches, which in turn generate distinct local lizard assemblages. It is generally well established that interacting historical and environmental factors explain hierarchical structures of assemblages [5]. However, empirical application is not common because it relies on efficient sampling designs to capture multiple scales [1]. In the megadiverse Amazonian rainforests this has been achieved by a few studies [73,12,31]. Considering the fine levels in which those studies have understood processes affecting biodiversity, efficient methods for multi-scale sampling should be prioritized by ecology and conservation biology.

Acknowledgments

Data collection was logistically supported by Programa de Pesquisas em Biodiversidade (PPBio), Centro de Estudos Integrados da Biodiversidade Amazônica (INCT-CENBAM), and Programa de Conservação da Vida Selvagem da Santo Antônio Energia S.A. Conselho Nacional de Desenvolvimento Científico e Tecnológico (CNPq) and Coordenação de Aperfeiçoamento de Pessoal de Nível Superior (CAPES) granted PhD scholarships to

GMP. CAPES provided a PNPD postdoc grant to RF and CNPq provided productivity grants to ILK and APL. We collected data under RAN-ICMBio / IBAMA permit nº 13777-2.

References

1. Magnusson WE, Braga-Neto R, Pezzini F, Baccaro F, Bergallo H, Penha J, et al. Biodiversidade e monitoramento ambiental integrado: o sistema Rapeld na Amazônia. First ed. Santo André- SP: Attema Editorial; 2013. Available from: https://ppbio.inpa.gov.br/sites/default/files/Biodiversidade_e_monitoramento_ambiental_integrado.pdf. cited 10 March 2019.
2. Pianka ER. Ecology and natural history of desert lizards: analyses of the ecological niche and community structure. New Jersey. Princeton University Press; 1986.
3. Schneider DC. The rise of the concept of scale in Ecology. Bioscience. 2001; 51: 545–556. doi: 10.1641/0006-3568(2001)051[0545:TROTCO]2.0.CO;2.
4. Gaston KJ, Blackburn TM. Macroecology and conservation biology. In: Blackburn TM, Gaston KJ, editors. Macroecology: Concepts and consequences. Oxford: Blackwell Science; 2003. pp. 345–367.
5. Vellend M. Conceptual synthesis in community ecology. Q Rev Biol. 2010; 85: 183–206. doi: 10.1086/652373; PMID: 20565040.
6. Antonelli A, Quijada-Mascareñas A, Crawford AJ, Bates JM, Velazco PM, Wüster W. Molecular studies and phylogeography of Amazonian tetrapods and their relation to geological and climatic models. In: Hoorn C, Wesselingh FP, editors. Amazonia, landscape and species evolution: a look into the past: Blackwell Publishing; 2010. pp. 386–404.

Field Code Changed

Field Code Changed

Field Code Changed

Field Code Changed

7. Buhrnheim CM, Cox-Fernandes C. Structure of fish assemblages in Amazonian Rain-Forest Streams: Effects of habitats and locality. Copeia. 2003; 2: 255–262. doi: 10.1643/0045-8511(2003)003[0255:SOFAIA]2.0.CO;2.

Field Code Changed

8. Barros OG, Cintra R. The effects of forest structure on occurrence and abundance of three owl species (Aves: Strigidae) in the Central Amazon Forest. Rev Bras Zoo. 2009; 26: 86–95. doi: 10.1590/S1984-46702009000100014.

9. Costa FRC, Magnusson WE, Luizão RC. Mesoscale distribution patterns of Amazonian understorey herbs in relation to topography, soil and watersheds. J Ecol. 2005; 93: 863–878. doi: 10.1111/j.1365-2745.2005.01020.x.

Field Code Changed

10. Haugaasen T, Peres CA. Mammal assemblage structure in Amazonian floodedand unflooded forests. J Trop Ecol. 2005; 2: 133–145. doi: 10.1017/S026646740400207X.

Field Code Changed

11. Menin M, Waldez F, Lima AP. Effects of environmental and spatial factors on the distribution of anuran species with aquatic reproduction in central Amazonia. Herpetol J. 2011; 21: 255–261.

12. Dias-Terceiro RG, Kaefer IL, Fraga R, Araújo MC, Simões PI, Lima AP. A matter of scale: historical and environmental factors structure anuran assemblages from the upper Madeira River, Amazonia. Biotropica. 2015; 47: 259–266. doi: 10.1111/btp.12197.

Field Code Changed

13. Ribeiro-Júnior MA. Catalogue of distribution of lizards (Reptilia: Squamata) from the Brazilian Amazonia. I. Dactyloidae, Hoplocercidae, Iguanidae, Leiosauridae, Polychrotidae, Tropiduridae. Zootaxa. 2015; 3983: 001–110. doi: 10.11646/zootaxa.3983.1.1.

Field Code Changed

14. Lobão PSP. Associações no uso do habitat por cinco espécies de lagartos amazônicos. M.Sc. Thesis, Instituto Nacional de Pesquisas da Amazônia. 2008. Available from: https://bdtd.inpa.gov.br/handle/tede/731. cited 10 March 2019.

15. Moraes LFP. Diversidade beta em comunidades de lagartos em duas Ecorregiões distintas na Amazônia. M.Sc. Thesis, Instituto Nacional de Pesquisas da Amazônia, Manaus. 2008. Available from: https://bdtd.inpa.gov.br/handle/tede/736. cited 10 March 2019.

16. Bittencourt S. A insularização como agente de fragmentação florestal em comunidades de lagartos da Amazônia Central. M.Sc. Thesis, Instituto Nacional de Pesquisas da Amazônia. 2008. Available from: https://bdtd.inpa.gov.br/handle/tede/1846. cited 15 March 2019.

17. Wallace AR. On the monkeys of the Amazon. J Nat Hist. 1954; 14 (2): 451–454. doi: 10.1080/037454809494374.

18. Dexter KG, Terborgh JW, Cunningham CW. Historical effects on beta diversity and community assembly in Amazonian trees. PNAS. 2012; 109: 7787–7792. doi: 10.1073/pnas.1203523109.

19. Hayes FE, Sewlal JN. The Amazon River as a dispersal barrier to passerine birds: effects of river width, habitat and taxonomy. J Biogeogr. 2004; 31: 1809–1818. doi: 10.1111/j.1365-2699.2004.01139. x.

20. Ribas CC, Aleixo A, Nogueira ACR, Miyaki CY, Cracraft J. A palaeobiogeographic model for biotic diversification within Amazonia over the past three million years. Proc Bio Sci. 2012; 279: 681–689. doi: 10.1098/rspb.2011.1120.

21. Pomara LY, Ruokolainen K, Young KR. Avian species composition across the Amazon River: the roles of dispersal limitation and environmental heterogeneity. J Biogeogr. 2014; 41: 784–796. doi: 10.1111/jbi.12247.

Field Code Changed

Field Code Changed

Field Code Changed

Field Code Changed

Field Code Changed

Field Code Changed

22. Fernandes AM, Gonzalez J, Wink M, Aleixo A. Multilocus phylogeography of the Wedge-billed Woodcreeper *Glyphorynchus spirurus* (Aves, Furnariidae) in lowland Amazonia: Widespread cryptic diversity and paraphyly reveal a complex diversification pattern. Mol Phylogenet Evol. 2013; 66(1): 270–282. doi: 10.1016/j.ympev.2012.09.033.

23. Simões PI, Lima AP, Magnusson WE. Acoustic and morphological differentiation in the frog *Allobates femoralis*: Relationships with the upper Madeira river and other potential geological barriers. Biotropica. 2008; 40: 607–614. doi: 10.1111/j.1744-7429.2008.00416.x.

24. Ayres JM, Clutton-Brock TH. River boundaries and species range size in Amazonian primates. Am Nat. 1992; 140: 531–537. doi: 10.1086/285427.

25. Boubli JP, Ribas C, Alfaro JWL, et al. Spatial and temporal patterns of diversification on the Amazon: A test of the riverine hypothesis for all diurnal primates of Rio Negro and Rio Branco in Brazil. Mol Phylogenet Evol. 2015; 82: 400–412. doi: 10.1016/j.ympev.2014.09.005.

26. Patton JL, da Silva MNF, Malcolm JR. Gene genealogy and differentiation among arboreal spiny rats (Rodentia: Echimyidae) of the Amazon Basin: a test of the riverine barrier hypothesis. Evol Lett. 1994; 48: 1314–1323. doi: 10.1111/j.1558-5646.1994.tb05315.x.

27. Ávila-Pires TC, Mulcahy DG, Werneck FP, Sites-Jr JW. Phylogeography of the teiid lizard *Kentropyx calcarata* and the Sphaerodactylid *Gonatodes humeralis* (Reptilia: Squamata): testing a geological scenario for the lower Amazon-Tocantins Basins, Amazonia, Brazil. Herpetologica. 2012; 68: 272–287. doi: 10.1655/HERPETOLOGICA-D-11-00021.1.

Field Code Changed

Field Code Changed

Field Code Changed

Field Code Changed

Field Code Changed

28. Kaefer IL, Tsuji-Nishikido BM, Mota EP, Farias IP, Lima AP. The early stages of speciation in Amazonian forest frogs: phenotypic conservatism despite strong genetic structure. Evol Biol. 2013; 40: 228–245. doi: 10.1007/s11692-012-9205-4.

29. Maia GF, Lima AP, Kaefer IL. Not just the river: genes, shapes, and sounds reveal population-structured diversification in the Amazonian frog *Allobates tapajos* (Dendrobatoidea). Biol J Linn Soc Lond. 2017; 121: 95–108. doi: 10.1093/biolinnean/blw017.

30. Souza SM, Rodrigues MT, Cohn-Haft M. Are Amazonia rivers biogeographic barriers for lizards? A study on the geographic variation of the spectacled lizard *Leposoma osvaldoi* Ávila-Pires (Squamata, Gymnophthalmidae). J Herpetol. 2013; 47:511–519. doi: 10.1670/12-124.

31. Moraes LJ, Pavan D, Barros MC, Ribas CC. The combined influence of riverine barriers and flooding gradients on biogeographical patterns for amphibians and squamates in south-eastern Amazonia. J Biogeogr. 2016; 43(11): 2113–2124. doi: 10.1111/jbi.12756.

32. Mariac C, Jehin L, Saïdou AA, Thuillet AC, Couderc M, Sire P, et al. Genetic basis of pearl millet adaptation along an environmental gradient investigated by a combination of genome scan and association mapping. Mol Ecol. 2011; 20(1): 80–91. doi: 10.1111/j.1365-294X.2010.04893.x.

33. Hangartner S, Laurila A, Räsänen K. Adaptive divergence in moor frog (*Rana arvalis*) populations along an acidification gradient: inferences from QST–FST correlations. Evol Letters. 2012; 66(3): 867–881. doi: 10.1111/j.1558-5646.2011.01472.x.

Field Code Changed
Field Code Changed
Field Code Changed
Field Code Changed
Field Code Changed
Field Code Changed

34. Kinupp VF, Magnusson WE. Spatial patterns in the understorey shrub genus Psychotria in central Amazonia: effects of distance and topography. J Trop Ecol. 2005; 21: 363–374. doi: 10.1017/S0266467405002440.
35. Emilio T, Quesada CA, Costa FRC, Magnusson WE, Schietti J, Feldpausch TR, et al. Soil physical conditions limit palm and tree basal area in Amazonian forests. Plant Ecol Divers. 2013; 7: 215–229. doi: 10.1080/17550874.2013.772257.
36. Drucker DP, Costa FRC, Magnusson WE. How wide is the riparian zone of small streams in tropical forests? A test with terrestrial herbs. J Trop Ecol. 2008; 24: 65–74. doi: 10.1017/S0266467407004701.
37. Ribeiro-Jr JW, Lima AP, Magnusson WE. The effect of Riparian Zones on species diversity of frogs in Amazonian Forests. Copeia. 2012; 3: 375–381. doi: 10.1643/CE-11-117.
38. Rojas-Ahumada DP, Landeiro VL, Menin M. Role of environmental and spatial processes in structuring anuran communities across a tropical rain forest. Austral Ecol. 2012; 37: 865–873. doi: 10.1111/j.1442-9993.2011.02330.x.
39. Bueno AS, Bruno RS, Pimentel TP, Sanaiotti TM, Magnusson WE. The width of riparian habitats for understory birds in an Amazonian forest. Ecol Appl. 2012; 22(2): 722–734. doi: 10.1890/11-0789.1.
40. Menger J, Magnusson WE, Anderson MJ, Schlege M, Pe'er G, Henle K. Environmental characteristics drive variation in Amazonian understorey bird assemblages. PLoS ONE. 2017; 2(2): e0171540. doi: 10.1371/journal.pone.0171540.
41. Fraga R, Lima AP, Magnusson WE. Mesoscale spatial ecology of a tropic snake assemblage: the width of riparian corridors in central Amazonia. Herpetol J. 2011; 21: 51–57.

Field Code Changed

Field Code Changed

Field Code Changed

Field Code Changed

42. Fraga R, Ferrão M, Stow AJ, Magnusson WE, Lima AP. Different environmental gradients affect different measures of snake β-diversity in the Amazon rainforests. PeerJ. 2018; 6: e5628. doi: 10.7717/peerj.5628.
43. Pinto MGM. Diversidade Beta, métodos de amostragem e influência de fatores ambientais sobre uma comunidade de lagartos na Amazônia Central. M.Sc. Thesis, Instituto Nacional de Pesquisas da Amazônia. 2006. Available from: https://bdtd.inpa.gov.br/handle/tede/947. cited 15 March 2019.
44. Faria AS, Menin M, Kaefer IL. Riparian zone as a main determinant of the structure of lizard assemblages in upland Amazonian forests. Austral Ecol. 2019; 1: 1–9. doi: 10.1111/aec.12754.
45. Fauth JF, Crother BI, Slowinski EJB. Elevational patterns of species richness, evenness, and abundance of the Costa Rican leaf-litter herpetofauna. Biotropica.1989; 21: 178–185. doi: 10.2307/2388708.
46. Lieberman SS. Ecology of a leaf litter herpetofauna of a neotropical rain litter in functioning of forest ecosystems. Biol Rev. 1986; 81: 1–31.
47. Driscoll DA. Extinction and outbreaks accompany fragmentation of a reptile community. Ecol Appl. 2004; 14: 220–240. doi: 10.1890/02-5248.
48. Vitt LJ, Blackburn DG. Ecology and life history of the viviparous lizard *Mabuya bistriata* (Scincidae) in the Brazilian Amazon. Copeia. 1999; 916–927. doi: 10.2307/1446087.
49. Vitt LJ, Zani PA, Lima AP. Heliotherms in tropical rain forest: the ecology of *Kentropyx calcarata* (Teiidae) and *Mabuya nigropunctata* (Scincidae) in the Curuá-Una of Brazil. J Trop Ecol. 1997; 13: 199–220. doi: 10.1017/S0266467400010415.

Field Code Changed

Field Code Changed

Field Code Changed

Field Code Changed

50. Dixo MBO. Efeito da fragmentação da floresta sobre a comunidade de sapos e lagartos de serapilheira no sul da Bahia. M.Sc. Thesis. Instituto de Biociências da Universidade de São Paulo. 2001.

51. Aleixo A. Knowledge gaps, research priorities, and future perspectives on bird conservation in the Brazilian Amazon. In: De Luc AC, Devele PE, Benck GA, Goerck JM, editors. Áreas importantes para a Conservação das Aves no Brasil. Parte II-Amazônia, Cerrado e Pantanal. 1nd ed. São Paulo -SP: Save Brasil; 2009. pp. 59-69.

52. Ávila-Pires TCS, Vitt LJ, Sartorius SS, Zani PA. Squamata (Reptilia) from four sites in southern Amazonia, with a biogeographic analysis of Amazonian lizards. Bol Mus Para Emílio Goeldi Cienc Nat. 2009; 4(2): 99–118.

53. Schietti J, Martins D, Emilio T, Souza PF, Levis C, Baccaro FB. Forest structure along a 600 km transect of natural disturbances and seasonality gradients in central-southern Amazonia. J Ecol. 2016; 104(5): 1335–1346. doi: 10.1111/1365-2745.12596.

54. Cavalcante MMA. Hidroelétricas do Rio Madeira - RO: território, tecnificação e meio ambiente. M.Sc. Thesis, Universidade Federal do Paraná. 2012.

55. IBGE Instituto Brasileiro de Geografia e Estatística. Recursos naturais e meio ambiente: uma visão do Brasil. Rio de Janeiro: IBGE; 1997. Available from: http://biblioteca.ibge.gov.br/biblioteca-catalogo.html?id=27704&view=detalhes. cited 10 April 2019.

56. Bezerra RB, Trindade AG. Caracterização de parâmetros pluviométricos, térmicos do balanço hídrico climatológico e desmatamento de Porto Velho – RO. Geografia. 2006; 15(1): 65-80. doi: 10.5433/2447-1747.2006v15n1p65.

Field Code Changed

57. Gentry AH. Changes in plant community diversity and floristic composition on environmental and geographical gradients. Ann Mo Bot Gard. 1988; 75: 1–34. doi: 10.2307/2399464.

58. Burger J, Zappalorti RT. Nest site selection by Pine Snakes, *Pituophis melanoleucus*, in the New Jersey Pine Barrens. Copeia.1986; (1): 116–121.

59. Webb JK, Shine R. Out on a limb: conservation implications of tree-hollow use by a threatened snake species (*Hoplocephalus bungaroides*: Serpentes, Elapidae). Biol Conserv. 1997; 81(12): 21–33. doi: 10.1016/S0006-3207(96)00160-7.

Field Code Changed

60. Pringle RM, Webb JK, Shine R. 2003. Canopy structure, microclimate, and habitat selection by a nocturnal snake, *Hoplocephalus bungaroides*. Ecology. 2003; 84(10): 2668–2679. doi: 10.1890/02-0482.

Field Code Changed

61. Cintra BBL, Schietti J, Emilio T, Martins D, Moulatlet G, Souza P, et al. Soil physical restrictions and hydrology regulate stand age and wood biomass turnover rates of Purus-Madeira interfluvial wetlands in Amazonia. Biogeosciences. 2013; 10: 7759–7774. doi: 10.5194/bg-10-7759-2013.

62. Franklin E, Magnusson WE, Luizão FJ. Relative effects of biotic and abiotic factors on the composition of soil invertebrates' communities in an Amazonian savannah. Appl Soil Ecol. 2005; 29: 259–273. doi: 10.1016/j.apsoil.2004.12.004.

Field Code Changed

63. Oksanen J, Blanchet FG, Friendly M, Kindt R, Legendre P, McGlinn D, et al. Vegan: Community Ecology Package. R package version 1.17-1. 2010. Available from: http://CRAN.Rproject.org/package=vegan. cited 2 March 2019.

Field Code Changed

64. R Core Team. R: A language and environment for statistical computing. Vienna, Austria: R Foundation for Statistical Computing. 2019. Available from: https://cran.r-project.org/doc/manuals/r-release/fullrefman.pdf. cited 2 March 2019.

Field Code Changed

65. Laird NM, Ware JH. Random-effects models for longitudinal data. Biometrics. 1982; 38: 963–974.
66. Hurvich CM, Tsai CL. Regression and time series model selection in small samples. Biometrics. 1989; 76(2): 297–307. doi: 10.1093/biomet/76.2.297.
67. Gascon C, Lougheed SC, Bogart JP. Genetic and morphological variation in *Vanzolinius discodactylus*: a test of the river hypothesis of speciation. Biotropica. 1996; 28: 376–387. doi: 10.2307/2389201.
68. Smith AL, Bull CM, Gardner MG, Driscoll DA. Life history influences how fire affects genetic diversity in two lizard species. Mol Ecol. 2014; 23: 2428–2441. doi: 10.1111/mec.12757.
69. Dambros CS, Azevedo RA, Gotelli NJ. Isolation by distance, not rivers, control the distribution of termite species in the Amazonian rain forest. Ecography. 2016; 39: 1–9. doi: 10.6084/m9.figshare.1319582.v10.
70. Gibbs HL, Sovic M, Amazonas D, Chalkidis H, Salazar-Valenzuela D, Moura-Da-Silva AM. Recent lineage diversification in a venomous snake through dispersal across the Amazon River. Biol J Linn Soc Lond. 2018; 123(3): 651–665. doi: 10.1093/biolinnean/blx158.
71. Souza-Filho PWM, Quadros MLES, Scandolara JE, da Silva-Filho EP, Reis MR. Compartimentação morfoestrutural e neotectônica do sistema fluvial Guaporé-Mamoré-alto Madeira, Rondônia-Brasil. Braz J Geol. 1999; 29: 469–476. doi: 11005/10461.
72. Tikuka MM. Geoarqueologia e paleohidrologia da planície aluvial holocênica do rio Madeira entre Porto Velho e Abunã/RO. Amazon. Rev Antrop. 2012; 4: 252–257.

73. Gascon C, Malcolm JR, Patton JL, da Silva MNF, Bogart JP, Lougheed SC, et al. Riverine barriers and the geographic distribution of Amazonian species. PNAS. 2000; 97: 13672–13677. doi: 10.1073/pnas.230136397.

74. Williams BK, Nichols JD, Conroy MJ. Analysis and Management of Animal Populations. Academic Press. 2002; 1–817.

75. Miller DA, Weir LA, McClintock BT, Grant EHC, Bailey LL, Simons TR. Experimental investigation of false positive errors in auditory species occurrence surveys. Ecol App. 2012; 1665-1674.

76. Ansari MH, Cooper SJ, Schwarz MP, Ebrahimi M, Dolman G, Reinberger L, Gardner MG. Plio-Pleistocene diversification and biogeographic barriers in southern Australia reflected in the phylogeography of a widespread and common lizard species. Mol phylogen evol. 2019; 107–119.

77. Lamborot M, Eaton L. The Maipo River as a biogeographical barrier to Liolaemus monticola (Tropiduridae) in the mountain ranges of central Chile. J Zool Syst Evol. 1997;105–111.doi: 0947-5745/97/3503-0 105$14.00/0.

~~74.~~78. Kraft NJB, Adler PB, Godoy O, James EC, Fuller S, Levine JM. Community assembly, coexistence and the environmental metaphor. Funct Ecol. 2014; 29: 592–599. doi: 10.1111/1365-2435.12345.

~~75.~~79. Bobrowiec PED, Tavares VdC. Establishing baseline biodiversity data prior to hydroelectric dam construction to monitoring impacts to bats in the Brazilian Amazon. PloS ONE. 2017; 12(9): e0183036. doi: 10.1371/journal.pone.0183036.

~~76.~~80. Fraga RD, Lima AP, Magnusson WE, Ferrão M, Stow AJ. Contrasting patterns of gene flow for Amazonian snakes that actively forage and those that wait in ambush. J Heredity. 2017; 108(5): 524–534. doi: 10.1093/jhered/esx051.

Formatted: Font: (Default) Times New Roman, 12 pt

Formatted: Font: (Default) Times New Roman, 12 pt, Font color: Auto

Formatted: Font: (Default) Times New Roman, 12 pt, Not Italic, Font color: Auto

Formatted: Font: (Default) Times New Roman, 12 pt, Font color: Auto

Formatted: Font: (Default) Times New Roman, 12 pt, Font color: Auto

Formatted: English (United States), Pattern: Clear

Formatted: Font: (Default) Times New Roman, 12 pt

Formatted: Font: (Default) Times New Roman, 12 pt, Not Italic

Formatted: Font: (Default) Times New Roman, 12 pt

Formatted: Font color: Auto, English (United States), Pattern: Clear

Formatted: Font: (Default) Times New Roman, 12 pt

Formatted: Font: (Default) Times New Roman, 12 pt, Not Italic

Formatted: Font: (Default) Times New Roman, 12 pt

Formatted: Font: (Default) Times New Roman, 12 pt

Formatted: Font color: Auto, English (United States), Pattern: Clear

Formatted: Font: (Default) Times New Roman, 12 pt

Formatted: Font: (Default) Times New Roman, 12 pt, Not Italic

Formatted: Font: Not Italic

Formatted: Font: (Default) Times New Roman, 12 pt

Formatted: Font: (Default) Times New Roman, 12 pt

Formatted: Font: (Default) Times New Roman, 12 pt

Formatted: Font color: Auto, English (United States), Pattern: Clear

Field Code Changed

Field Code Changed

~~77.~~81. Silva JMC, Rylands AB, Fonseca GAB. The fate of the Amazonian areas of endemism. Conserv Biol. 2005; 19: 689–694. doi: 10.1111/j.1523-1739.2005.00705.x.

~~78.~~82. Fearnside MP. Brazil's Madeira River dams: A setback for environmental policy in Amazonian development. Water Altern. 2014; 7: 256–269.

~~79.~~83. Bernardi JVE, Lacerda LD, Dórea JG, et al. Aplicação da análise das componentes principais na ordenação dos parâmetros físico-químicos no alto Rio Madeira e afluentes, Amazônia Ocidental. Geochim Brasiliensis. 2012; 23: 79–90.

~~80.~~84. Hoogmoed MS, Ávila-Pires TC. Studies on the species of the South American lizard genus *Arthrosaura Boulenger* (Reptilia: Sauria: Teiidae), with the resurrection of two species. Zool Meded. 1992; 66(35): 453–484.

~~81.~~85. Vitt LJ, Zani PA, Espósito MC. Historical ecology of Amazonian lizards: implications for community ecology. Oikos. 1999; 87: 286–294. doi: 10.2307/3546743.

~~82.~~86. Daws M, Mullins C, Burslem D, Paton S, Dalling J. Topographic position affects the water regime in a semideciduous tropical forest in Panama. Plant Soil Environ. 2002; 238: 79–90. doi: 10.1023/A:1014289930621.

~~83.~~87. Fischer J, Lindenmayer DB. The sensitivity of lizards to elevation: A case study from south-eastern Australia. Divers Distrib. 2005; 11: 225–233. doi: 10.1111/j.1366-9516.2005.00139.x.

~~84.~~88. Pansonato MP, Costa FRC, Castilho CV, Zuquim G. Spatial scale or amplitude of predictors as determinants of the relative importance of environmental factors to plant community structure. Biotropica. 2013; 45: 299–307. doi: 10.1111/btp.12008.

~~85.~~89. Tews J, Brose U, Grimm V, Tielborger K, Wichmann MC, Schwager M, et al. Animal species diversity driven by habitat heterogeneity/diversity: the importance

Field Code Changed

Field Code Changed

Field Code Changed

of keystone structures. J Biogeogr. 2004; 31: 79–92. doi: 10.1046/j.0305-0270.2003.00994.x.

Field Code Changed

~~86.~~90. Ballinger RE. Intraspecific variation in demography and life history of the lizard, *Sceloporus jarrovi*, along an altitudinal gradient in southeastern Arizona. Ecology. 1979; 60: 901–909. doi: 10.2307/1936858.

Field Code Changed

~~87.~~91. Naiman RJ, Decamps H, Pollock M. The roles of riparian corridors in maintaining regional biodiversity. Ecol Appl. 1993; 3: 209–212. doi: 10.2307/1941822.

Field Code Changed

~~88.~~92. Rocha CFD. Introdução à ecologia de lagartos brasileiros. In: Nascimento LB, Bernerdes AT, Cotta GA, editors. Herpetologia no Brasil. 1nd ed. Minas Geirais, BR: PUCMG, Fundação biodiversidade and Fundação Ezequiel Dias; 1994. pp. 1–68.

~~89.~~93. Richardson JL. Divergent landscape effects on population connectivity in two co-occurring amphibian species. Mol Ecol. 2012; 21: 4437–4451. doi: 10.1111/j.1365-294X.2012.05708.x.

Field Code Changed

~~90.~~94. Fouquet A, Courtois EA, Baudain D, Lima JD, Souza SM, Noonan DP, et al. The trans-riverine genetic structure of 28 Amazonian frog species is dependent on life history. J Trop Ecol. 2015; 31: 361–373. doi: 10.1017/S0266467415000206.

Field Code Changed

~~91.~~95. Brandt R, Navas CA. Life-history evolution on Tropiduridae lizards: influence of lineage, body size and climate. PLoS ONE. 2011; 6: e20040. doi: 10.1371/journal.pone.0020040.

Field Code Changed

~~92.~~96. Cole EM, Bustamante MR, Almeida-Reinoso D, Funk WC. Spatial and temporal variation in population dynamics of Andean frogs: Effects of forest disturbance and evidence for declines. Glob Ecol Conserv. 2014; 1: 60–70. doi: 10.1016/j.gecco.2014.06.002.

Field Code Changed

~~93.~~97. Vitt LJ, Carvalho CMD. Life in the trees: the ecology and life history of *Kentropyx striatus* (Teiidae) in the Lavrado area of Roraima, Brazil, with comments on the life histories of tropical teiid lizards. Can J Zool. 1992; 70(10): 1995–2006. doi: 10.1139/z92-270.

Field Code Changed

~~94.~~98. Hoorn C, Wesselingh FP, Ter Steege H, Bermudez MA, Mora A, Sevink J, et al. Amazonia through time: Andean uplift, climate change, landscape evolution, and biodiversity. Science. 2010; 330(6006): 927–931. doi: 10.1126/science.1194585.

Supporting information captions

S1 Text. Protocols for measuring the environmental predictors~~**gradients**~~**.** Predictors~~Gradients~~ used as independent variables in the ecological models to test lizard assemblage structuring in the upper Madeira River, Amazonian Brazil.

S2 Fig 1. Partials from Mixed Linear Models for the west bank of the Madeira River. Test the effects of environmental predictors~~gradients~~ on lizard assemblages composition (PCoA axis 1). The models were selected by ΔAICc < 2. (A) Ilha das Pedras (B) Ilha dos Búfalos (C) Jirau-West (D) Teotônio.

S3 Fig 2. Partials from Mixed Linear Models for the East bank of the Madeira River. Test the effects of environmental predictors~~gradients~~ on lizard assemblages composition (PCoA axis 1). The models were selected by ΔAICc < 2. (A) Jaci- Paraná (B) Jirau-East (C) Morrinhos.

REBUTTAL LETTER

PONE-D-19-15180
Hierarchical effects of historical and environmental factors on lizard assemblages in the upper Madeira river, Amazonian Brazil
PLOS ONE

Responses to editor comments:

1. Please ensure that your manuscript meets PLOS ONE's style requirements, including those for file naming. The PLOS ONE style templates can be found at http://www.journals.plos.org/plosone/s/file?id=wjVg/PLOSOne_formatting_sample_main_body.pdf *and* http://www.journals.plos.org/plosone/s/file?id=ba62/PLOSOne_formatting_sample_title_authors_affiliations.pdf

Response: All PloS ONE style standards have been revised.

2. *We note that Figure 1 in your submission contain [map/satellite] images which may be copyrighted. All PLOS content is published under the Creative Commons Attribution License (CC BY 4.0), which means that the manuscript, images, and Supporting Information files will be freely available online, and any third party is permitted to access, download, copy, distribute, and use these materials in any way, even commercially, with proper attribution. For these reasons, we cannot publish previously copyrighted maps or satellite images created using proprietary data, such as Google software (Google Maps, Street View, and Earth). For more information, see our copyright guidelines:* http://journals.plos.org/plosone/s/licenses-and-copyright.

We require you to either (1) present written permission from the copyright holder to publish these figures specifically under the CC BY 4.0 license, or (2) remove the figures from your submission:

Response: The satellite image used to compose Figure 1 was changed following the proposed guidelines and a new image was added in its place from The Gateway to Astronaut Photography of Earth (public domain).

Response to Reviewers

1. *My main concern is that all inference here is based on count and presence/absence data, which may be influenced by sampling errors. For instance, you may find 20 individuals of species A and 19 individuals of species B in a given plot, when in fact there was 120 individuals of species A and 20 individuals of species B. This is typically a false-negative type error. Alternatively, you may have misidentified species B, inducing false-positive errors in your counts. Similar problems occur when using presence/absence data when absence is not included and estimated in the model. This subject is well covered in the literature. You may want to have a look at Kéry and Schmidt 2008 (Community Ecology 9: 207-216), Fitzpatrick et al. 2009 (Ecological Applications 19: 1673-79), Miller et al. 2012 (Ecological Applications 22: 1665-74) and so on. Since sampling effort seems to be enough, I recommend applying a modeling technique that accounts for sampling errors (for instance, N-mixture models), which I find more appealing to discuss results arising from count data. However, I understand that you would have to make a major change in your manuscript, especially if results change, which I would not be surprised. At least, I recommend including a paragraph discussing this subject, since I find hard to believe that you have captured the exactly number of individuals and species present at a given site, and thus, the results may be biased.*

Response:

We agree with the reviewer that when we study ecological data based on abundance and presence / absence data, we may be masking sampling errors since every technique is biased and will depend on the probability of species detection. However, as in our study we conducted multiple data collections at every point, combined with more than one sampling method, we think that we minimized sampling bias as much as possible.

As for the suggested modeling technique (N-mixture models): we opted for the second option suggested by the reviewer, which would be to continue using the models already proposed and not change our analyzes. We add a paragraph discussing these type I and II errors and the possible biased results generated from the sampling and that could be altering our results in some way. We justify our decision not to use N-mixture models because the same consist of two linked generalized linear models, however, for the realization of this model, the ordering method chosen by us (principal coordinate analysis - PCoA) cannot be used since it does not support the input of negative values. Given that the PCoA returns negative values of scores on its axes we could not be using this sorting method.

Therefore, we chose to continue with PcoA to the detriment of the other sorting methods that could be substituted to meet the assumption of generalized linear models, since our choices followed the one suggested by GOTELLI & ELLISON (2011, page 446 of the book Principles of Statistics in Ecology). These authors recommend the use of PcoA for “sorting applications in which the objective is to preserve the original multivariate distances between observations in the reduced space (sorting)”, especially when we intend to use the axes generated in the ordering in later analyzes. With regard to the use of other methods such as the widely used NMDS, the axes are not orthogonal, that is, we could not choose just one of the axes as a predictor of our species composition.

We carried out several previous analyzes with other sorting methods, different types of returns, and simulations including the removal of species that presented low abundance. In each simulation we had the precaution to show to other researchers in the area and request their opinions before deciding on the methods presented in this version of our manuscript. Among these simulations, we carried out the NMDS which captured 46.64% of the variation in species composition with two axes and demonstrated that the lizard assemblages differed significantly in relation to the banks of the Madeira River (MANOVA: Pillai trace = 0,205, $F_{1,80}$= 10,229, p <0,001). As for the results of the regressions with the environmental variables, the ordering of the NMDS plots for the left side of the river was only possible using three NMDS axes due to the little variation in the data with two axes (which led us to prefer the analysis main coordinates - PcoA), and as a result captured 70% of the variation in species composition. The explanation for the right margin using two NMDS axes was 57%. As for the possible structuring environmental variables, the effect of the vegetation structure (number of trees) on the left side ($F_{7,38}$=2,567, p=0,04), and for the right margin, only elevation was identified as a factor influencing the composition of the lizard assemblages ($F_{1,70}$=7,737, p<0,001). With all the simulations with our data we can see a strong effect of the modules themselves and the need to analyze the modules Ilha da Pedra (west bank) and Jirau-D (east bank) separately from the rest of the sample units, so we arrive at our current results .

2. *Your sample includes temporal variation (extended for a year, or so) but there are no temporal covariates among your predictors. Lizard activity is usually related to temperature, sunlight, rainfall and so on. You probably sampled plots in different days and different hours within a day, and ciclicity occurs even within individuals. How do you think the absence of temporal predictors affect your results? For instance, Figure 2 shows species ‘abundances on each river bank. Some species seems to be common on one bank, but not the other. Is this really the barrier effect of the river? By ignoring temporal variability, you assume sampling did not vary along the year.*

Response:

Each sampling campaign was carried out at a different time of the year: Campaign I- 24 February to 26 April 2010; Campaign II- July 30 to August 19 2010; Campaign III- November 5 to 26 2010; and Campaign IV- January 13 to

February 4 2011. In each of these campaigns, all plots inserted in the different modules were sampled, that is, each plot was sampled in four different times of the year. Therefore, we think we minimized the sampling bias regarding the presence / absence of species in the plots. In fact, the activity of the lizards is closely related to variations in temperature, light and precipitation in a given environment. With this in mind, the plots were sampled at different times. We believe that our sampling method that combined active visual search, with two simultaneous observers, supplemented by sweeping the leaf-litter, minimized the lack of temporal predictors in the presence/absence results.

3. *Authors suggest that the pattern found – the river acting as a barrier – was important. However, they mention that the lizard fauna found is widely distributed over the Amazon. This is somewhat confusing to me because if species were well-distributed, they would probably be found everywhere. To explain that, authors mention that the barrier effect of the river may vary, suggesting seasonality. This makes me wonder if species are truly isolated, as suggested. The barrier effect could be only temporarily and the results found are explained by the time frame of the study. I would like authors to clarify that point.*

Response:

Actually, most of the lizard species found in our study are widely distributed across Amazonia, and when we say that these species are isolated, we are not trying to extrapolate our results to the entire length of the river, so we were careful to keep that registered in the discussion (from line 385). as we do not have information for other locations along the length of the Madeira River.

We did not want to suggest seasonality when we wrote that the river barrier effect may vary. The different Amazonian rivers (and even different parts of a same large river) have distinct widhts, current speeds, water types and geological dynamics, which may affect their role as an effective geological barrier.

Even widely distributed species in Amazonia are not necessarily found everywhere, since the configuration of the Amazon Basin can be related to a mosaic of different phyto-regions, observed both on a local and regional scale, with different geological ages and origins between the different fractions of the basin, a fact that leads to historical evolutionary differences between areas and that become determinant factors for the differences in lizard species richness in the region. Even within each area of endemism, with no current geographical barriers, ecological differentiation can occur (Ferrão *et al*., 2017; Ortiz et al. 2018). The isolation of lizard species in one of the banks of the river may be related to geomorphological heterogeneity along the course of the Madeira River, and even at different sampling dates, we still observe the restricted presence of some species to certain banks of the river.

Ferrão, M.; Moravec, J.; Fraga, R.; Almeida, A.P.; Kaefer, I.L.; Lima, A.P. 2017. A new species of Scinax from the Purus–Madeira interfluve, Brazilian Amazonia (Anura, Hylidae). *ZooKeys*, 706: 137–162.

Ortiz DA, Lima AP, Werneck FP (2018) Environmental transition zone and rivers shape intraspecific population structure and genetic diversity of an Amazonian rain forest tree frog. Evolutionary Ecology 32(4):359-378

4. I *think your manuscript would benefit from contrasting your results with other lizard systems around the world. For instance, are there examples of rivers acing as barriers for lizards elsewhere? What about other reptiles? What are the covariates related to isolation for lizards or reptiles in general? In addition, you could provide some information about the Amazon basin. For instance, what is the mean width of the big rivers? Finally, concerning the species found. What is the habit of the most abundant species? Did you find terrestrial, arboreal and fossorial species equally? Does you results relate to species 'habits? I believe that finding terrestrial species (the teiids, for instance) may be easier than arboreal (Enyalius?) or fossorial. In the same way, diurnal species will be easier to find during the day and the opposite for nocturnal. How was the sampling scheme? Can you really talk about lizards in general of a guild?*

Response:

We thank the reviewer and agree that some information needs to be added, so we have included in the discussion other examples of lizards in the Amazon and around the world, and data on the covariates associated with restriction of geographic distributions. Information on the width of the largest Amazonian rivers was added to the methodology when mentioning the Madeira River, as it is one of the largest and most important rivers in the Amazon basin. Regarding the species habits, we found the tree species *Gonatodes humeralis*, and *Norops fuscuoratus* as the most abundant, followed by the teiid *Ameiva ameiva*. The fossorial or semi-fossorial species were the ones with the lowest abundances, a fact already expected and observed in different studies on lizard assemblages. We think that the results are related to the habits of the species, as included in the discussion (starting from line 428), where we associate the results of the gradient number of trees to some tree species found.

Regarding the species activity time (more detail in the following answers to the reviewer below), most species are diurnal or crepuscular and few are nocturnal, but easily detectable to experienced researchers in the region.

We observed that our sample is within the richness already highlighted in the local and regional literature for Amazonia, and we think we gave a good representation of how the lizard community in that region is structured.

With regard to the term guild, we decided not to apply because the observed species use different and complementary resources, thus not constituting a specific guild with regard to diet or habitat use, for example.

Minor comments

✓ L62: substitute "design" by "designed"

Response: Replaced in text

✓ L84: change "the latter" by "lizards"

Response: Replaced in text

✓ L161: I am not sure I would exclude those sites since you may overestimate predictor effects.

Response: We decided to maintain the exclusion, as some of these sites coincided with areas of difficult access, for example, due to fallen trunks, making it difficult to travel along the plot, or even partially flooded, and could contribute to the low probability of detection. Thus, we decided to exclude all sites that contained zero individuals.

✓ L166-167: you have four surveys on each plot. I suppose there was variation on specie´s counts over surveys, due to several aspects, including seasonality (which you ignored). I advise on modeling this variability on counts.

Response: Question previously answered.

✓ L168-169: what time did you search? Are all lizards diurnal/nocturnal? Any potential bias here?

Response: Most of Amazonian lizards are diurnal. Even those that start their activity at dusk, such as *Uranoscodon superciliosus* (Linnaeus, 758), are easy to

detect because they usually rest over tree branches while inactive. Regarding campaign schedules, all were carried out during the day. However, the first and last were carried out in the afternoon and early evening, and the second and third were carried out in the morning and early afternoon. Thus, we contemplated different activity periods, maximizing detectability.

- ✓ L177: I would rather call these ‘environmental predictors’, since gradients may remind other things as well.

Response: Replaced in text.

- ✓ L183: change ‘.’ by ‘,’

Response: Replaced in text.

- ✓ L221-223: maybe it is just me, but the way it is written seems that you avoided correlated models, not predictors.

Response: The sentence was rewritten for clarity.

- ✓ L223-226: what was the cut-off point?

Response: From r = 0.51. We added this information in the text.

- ✓ L233: what do you mean by “corrected for few parameters”?

Response: We wanted to say that we did use Akaike´s Information Criterion (AIC) for model selection, but the one corrected for small samples: the AICc. The sentence was poorly written in the first version and rephrased for clarity.

- ✓ L234-235: what about the non-nested models? Why using the cut-off point if important model weight may be left out? Isn´t model averaging a better strategy depending on the case?

Response: I am not sure if I understood the question, and in what our analysis differs from what has already been done in the literature. We stipulated the ΔAICc <2 cut, due to the number of possible models with different combinations between the variables, and this cut would inevitably exclude some models at the expense of those more parsimonious models.

- ✓ L261-262: how can you be sure those species were not there? Table 2. what kind of models did you build? Only those with main effects, excluding additive and interactive effects? Did you try combining all best predictors in one model? Figure 3. which model did you use to plot the relationship between abundance and number of trees? The top model or all three models? Notice that model weights are similar and all of them include the number of trees. Table 3: This table is somewhat hard to read. Please, insert a backspace between West and East or a line between them. In addition, maybe you could reduce the names of the predictors or show their first letters only.

Response: Yes, several models were built, with different combinations between different predictors that did not correlate with each other. In relation to figure 3, we show a direct ordination of the values from the gradient number of trees and the relative abundance of species by plots. With regard to the table, we added additional lines as suggested by the reviewer for a better visualization.

- ✓ L293: again, I would rather call 'predictor' or 'covariate' instead of 'gradient'.

Response: Replaced in text.

- ✓ L294: which model? Not the top model nor the third best-ranked model, if I read the table correctly (please, revise the table configuration). However, all models present similar AIC weights. How was the rationale behind using the only model that presented a significant effect? Did you try model averaging? I would not pick just one model among several to present a result, especially if they diverge. Figure 4. Same comment as in Figure 3.

Response: We decided to graphically show only the significant models ($P < 0.05$). We could really include the images of all the models (which were marginally significant), but we wanted to be synthetic and illustrate these results with a single image due to the length of the article.

✓

L352: there is a typo in this line.

Response: Corrected in text.

✓

L389-390: I am not convinced about that without including temporal predictors in the analysis. Was it too hot or too cold during some of the samplings? Were there thunderstorms or heavy rainfall in, at least, some of the sampling occasions?

Response: Samples were not taken in the rain. When this happened the samplings were postponed.

✓

L400-405: I find the discussion on conservation implications interesting, but I am not sure you provided all the elements for that. For instance, what kind of forest did you measure, native on invasive? Were there pine plantations (which are also categorized as forests)? How is the relationship between lizard assemblage and forests in general?

Response: All sampling modules were installed in original, preserved Amazonian *terra-firme* forests that are not influenced by other types of vegetation.

✓

L419: you start this paragraph stating that species turnover was influenced by the distance from the river. But this was not found in all samples, as you stated in the same paragraph. Given that your samples covered only 5 km from the river, which in the Amazon, does not seem to be a huge area, what distance do you think you would find such effect?

Response: We believe that species turnover is related to several aspects in addition to the distance from the river, and that stipulating a margin distance value for the Amazon is a risk, since the region has a large environmental heterogeneity between the interfluvial regions, and even over small distances of 5km we could have significant results in the turnover of species, especially those less vagile. However, many gaps need to be filled for Amazonian lizards, despite recent efforts to answer such a question.

✓

L427-428: what do you mean by "...was returned..."?

Response: We used returned in the sense that it was observed, but the word was replaced in the text.

✓

L451-452: what do you mean by "efficient methods for multi-scale sampling"? Can you provide suggestions about what you would say is an efficient method?

Response: Methods that present a spatial standardization, easily replicable for different species (modular), and preferably that can be carried out for a long time. An example of a good method is the one used here in our study, the RAPELD-Rapid Assessments and Long-term Ecological Research (Magnusson et al., 2005). These modules are part of a network of permanent standardized transects installed in the Amazon by the Biodiversity Research Program (PPBio) of the Brazilian Science, Technology, Innovations and Communications Ministry (Magnusson et al., 2013). An example of sample desing is shown in the figure below. In this method the plots follow the terrain level curve, minimizing possible topographic (environmental) effects within the plot (sampling unit). RAPELD has already been used in several localities around the world https://ppbio.inpa.gov.br/sites/default/files/Biodiversidade_e_monitoramento_ambiental_integrado.pdf. cited 10 March 2019.

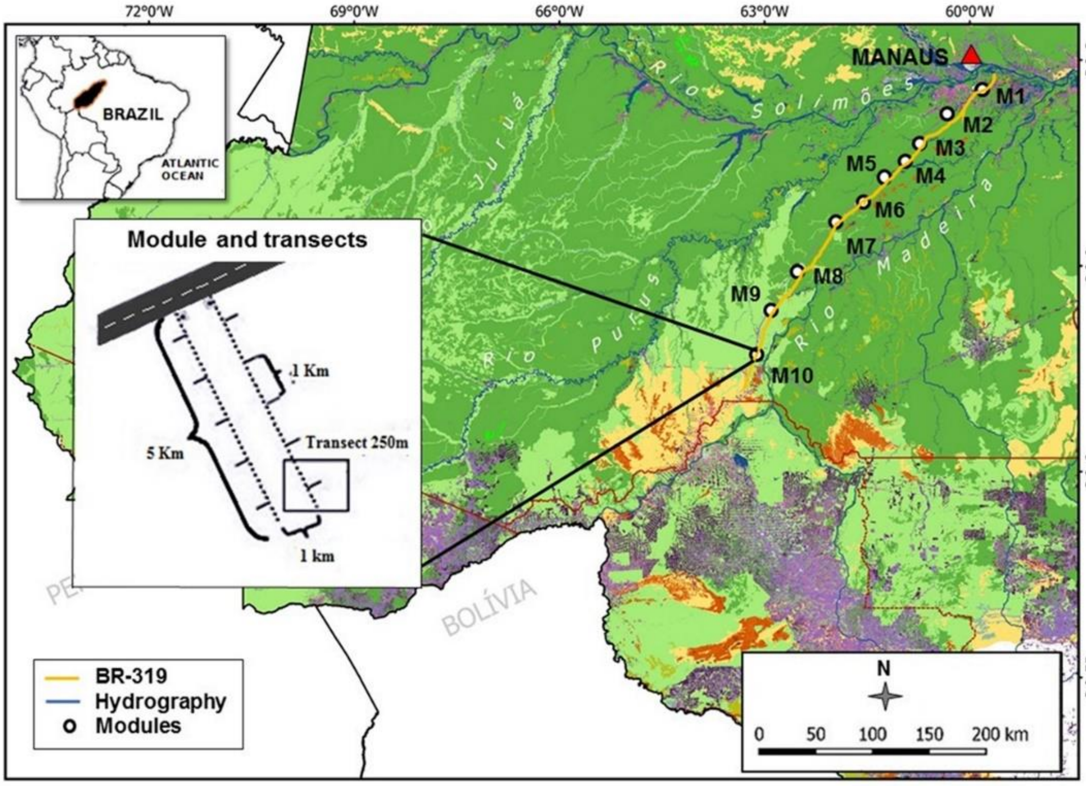

Supplement: Attachment — Submitted filename: PONE-D-19-15180_R1.pdf [file pone.0233881.s005.pdf]
