# Supplementary material for: Hierarchical effects of historical and environmental factors on lizard assemblages in the upper Madeira River, Brazilian Amazonia
Source: PLoS One. 2020 Jun 2;15(6):e0233881. doi: 10.1371/journal.pone.0233881 (PMC7266318; doi:10.1371/journal.pone.0233881)
Supplement: Attachment — Submitted filename: Comments Rev#1.pdf [file pone.0233881.s006.pdf]

Dear all,

A previous reviewer expressed his/her concerns on possible sampling errors due to the methods used to estimate species presence/abundance. The authors answered that some of those errors were minimized by using “more than one sampling method”. Indeed, besides the visual searching, they only referred searching amidst leaf litter or under debris, and stated that this could enhance detection of fossorial/semi-fossorial species. However, there is no strictly fossorial species in the assemblage reported, and such species in reality could barely be found just by sweeping the leaf-litter. Moreover, although the authors provided responses to most of the reviewer’s concerns, they made no comment on the possibility that some of the taxa indeed represent cryptic species. I suggest them to discuss this it a little bit.

Some of the points raised by the reviewer on item 4 of his/her review also deserve additional efforts from the authors. As an example, mean width of the upper Madeira River was not mentioned anywhere, but this is an important issue when hypothesizing it could act as a riverine barrier. Also, information on sampling scheme are not yet clear enough. Visual searches were performed during the day only, or also during the night, since there are nocturnal species in the assemblage studied?

The former reviewer also pointed that “authors suggest that the pattern found – the river acting as a barrier – was important. However, they mention that the lizard fauna found is widely distributed over the Amazon.” He then made some comments on this topic. The authors provided a pertinent response but I think the discussion on this topic could benefit from adding supporting literature on known distributions of some of the species treated therein, which are based in more historical datasets and which clearly show the pattern discussed.

Minor comments

Page 1

Line 3 - "Brasil Amazônico" does not make sense... Brazilian Amazonia would be more appropriated

Page 2

Line 35 - Brazilian Amazonia

Line 36 - you have used "river" in the title and "River" from now on. Check and standardize it throughout the text - River or river.

Line 39 - delete space

Line 44 - "along" would be better

Line 46 - "... over the extensive (nearly xxx km) study area." (I suggest you to briefly mention in the abstract the magnitude of the area to which these results aplly)

Page 3

Line 72 - first cite frogs, then birds. Please also sort citations in ascending order, throughout the text.

Line 72 - first cite spiny rats, then primates

Page 4

Line 82 - insert an Oxford comma before "and"; see additional remarks on it in line 182

Line 82 - Ideally, you should cite them in an evolutionary rank. Then you will need to renumber references. Please also sort citations in ascending order.

Line 84 - sort citations in ascending order

Line 85 - sort citations in ascending order

Page 5

Line 103 – replace “e” by “and”

Line 106 - Brazilian Amazonia

Line 116 - basin (the "megadiverse region" you want to better understand is that one under the influence of the river, not the river itself).

Line 120 - delete space

Line 120 - insert space

Line 122 - As one of your hypothesis treats the upper Madeira as a barrier, please add some information on relevant characteristics of the river itself (e.g., river width).

Line 123 - Brazilian

Line 123 - River (standardize)

Line 124 - River

Line 124 - check standards of the journal (Fig instead of Fig.). Additionally, please add some information on the endemism zones (Rondônia and Inambari), so the mention to them can be properly evaluated when analyzing the elements in your Fig 1.

Page 6

Line 126 - And what does it mean? These two endemism zones are only briefly mentioned in the Introduction and here in the legend for Figure 1. You should briefly discuss on the implications of this in your results.

Page 7

Line 153 - check and standardize throughout the text: you used either with or without hyphen

Line 154 - see comment on line 153

Lines 165-166 - Dates should follow a standardized sequence (e.g.,day-month-year, as you used here)

Line 172 – there is no fossorial species in your list.

Line 174 - at what time of the day/night?

Page 8

Line 178 – replace squamates by lizards

Line 181 – soil in lowercase

Line 182 - Note: check journal style and consistently use (or do not use) the Oxford comma throughout all the text. In this paragraph, as an example, you alternatively used it (see line 180 - "soil texture, fertility, and flat-level") and did not use it (e.g., in lines 179 - "...foraging, resting and thermoregulation sites", and line 182 - "...Potassium, Zinc and exchangeable Aluminum").

Line 184 – remove the dot before citation [61]

Page 9

Line 204 - along the same river bank (replace "within a" by "along the same")

Page 10

Line 224 - insert space between "predictors" and "that"

Line 247 – bring to the suggested point the mention to Table 1, or, alternatively, to a point immediately after the mention of the three most abundant species. The information on the number of plots where each species was found do not appear in the table.

Page 11

Line 251 - remove the mention to Table 1 here, and insert it at any point in the previous sentence. There is no information in Table 1 on the number of plots where each species was found.

Table 1 – check correspondence of values to each species: the values highlighted possibly correspond to *Alopoglossus angulatus*. Please relocate them to the correct line of the table.

Page 12

Table 1 – complete year of description of *Kentropyx calcarata* and of *Uranoscodon superciliosus*

Line 265 - insert an Oxford comma after *bassleri*

Line 267 - "and" not in italics. Please also add an Oxford comma before "and" .

Line 272 - replace "of" by "in" ; replace “and” by “or”

Line 273 – add “bank” after “east”

Page 13

Line 286 - insert an Oxford comma after “Búfalos”

Line 288 - standardize mention to the Akaike's criterion: "?AICc < 2", as used before.

Page 14

Table 2 - correct the word "margim". Check it in every Table. It appears more than once.

Line 292 – insert "to" before "the number"

Page 15

Line 305 - standardize mention to the Akaike's criterion: "?AICc < 2", as used before.

Table 3 - correct the word "margim". Check it in the table.

Page 16

Table 3 - correct the word "margim".

Lines 312-313 – Principal Coordinates Analysis (cite it according to the abbreviation PCA)

Page 17

Line 331 - “East Jirau” without hyphen. Standardize it throughout the text.

Line 332 - insert an Oxford comma between "sand, and clay"

Line 333 - see comments on line 312.

Line 342 - ...[18], diurnal frogs [12], birds... (cite diurnal frogs before birds)

Line 346 – “...with those obtained for frog assemblages”

Page 18

Lines 355-356 or elsewhere: did you ever discuss on the possibility of taxonomic bias contributing to this scenario? There are consistent and recent efforts to investigate the taxonomic status of frog species in the region, and such efforts are unparalleled regarding lizards. Maybe you can use recent literature to briefly discuss on that. Only 19% of the species in Table 1 were described in the 20th century, while 31% of the frogs considered in Dias-Terceiro et al. (2015) were described in 21th century and other three species were recognized as undescribed by the same authors.

Line 360 - you could mention the distribution patterns for some of the species considered herein, depicted in Ribeiro Jr. (2015), as an additional source of evidence

that the pattern you found is real, even though a few specimens were recorded during your study (the distribution shown for *E. leechii*, as an example, based in a large dataset).

Lines 362-363 - again, taxonomic bias may be also one of causes of such inconsistencies. What you are calling *Kentropyx altamazonica*, as an example, potentially represents a cryptic species, as other taxa in your Table 1. This is out of the scope of your work, but cannot be ignored

Line 363 - sort citations in ascending order

Page 19

Line 385 - you basically used "active visual search". Did you additionally use a systematic search using leaf litter plots? Or you merely searched leaf litter anurans instead, by "sweeping the leaf litter and removing debris"? Looking carefully at every microhabitat visually accessible and inspecting potencial retreats are common procedures during visual searches.

Line 401 - cite in an evolutionary rank

Line 403 – replace "within a" by "along a same"

Page 20

Line 410 - of which zoological group? is this a general trend for chordates? for reptiles? for tropical reptiles? your supporting references herein (58-60) are for snakes, including a temperate species. Can you replace or complement them with any citation on the effects of vegetation heterogeneity on tropical lizards?

Line 413 - insert an Oxford comma after “humidity”

Page 21

Line 435 - insert an Oxford comma after “cover”

Line 437 – “...snake [41], and bird [39] assemblages.”

Line 447: insert “upper” before Madeira River

Page 22

Line 463 - sort citations in ascending order

Page 33

Line 725 - italicize *Liolaemus monticola*

Page 34

Line 746 - not in italics

Page 36

Line 803 - West Jirau (standardize)

Line 801 - check

Line 805 - check

Line 806 - East Jirau (standardize)

Line 806 – delete extra space before “Paraná”
