# Supplementary material for: Hierarchical effects of historical and environmental factors on lizard assemblages in the upper Madeira River, Brazilian Amazonia
Source: PLoS One. 2020 Jun 2;15(6):e0233881. doi: 10.1371/journal.pone.0233881 (PMC7266318; doi:10.1371/journal.pone.0233881)
Supplement: Attachment — Submitted filename: Response to Reviewers R2 5May2020.pdf [file pone.0233881.s007.pdf]

Manaus, May 5th 2020.

RESPONSE LETTER

PONE-D-19-15180 - Second round of review

Hierarchical effects of historical and environmental factors on lizard assemblages in the upper Madeira River, Brazilian Amazonia

Dear Dr. Stefan Lötters,

Thank you very much for such detailed revisions on our manuscript. Please find below our point-by-point letter addressing all comments and suggestions. Responses are marked in blue.

Kind regards,

The authors

Responses to comments:

Dear all,

A previous reviewer expressed his/her concerns on possible sampling errors due to the methods used to estimate species presence/abundance. The authors answered that some of those errors were minimized by using "more than one sampling method". Indeed, besides the visual searching, they only referred searching amidst leaf litter or under debris, and stated that this could enhance detection of fossorial/semi-fossorial species. However, there is no strictly fossorial species in the assemblage reported, and such species in reality could barely be found just by sweeping the leaf-litter. Moreover, although the authors provided responses to most of the reviewer's concerns, they made no comment on the possibility that some of the taxa indeed represent cryptic species. I suggest them to discuss this it a little bit.

Response: Thank you very much for these comments. In this revised version of the manuscript we highlighted that "search on the vegetation and on the leaf litter was systematically conducted in the first and second half of each visual sampling in the plots, respectively, thus constituting two different and complementary methods". With regard to the assemblage of fossorial species, now we acknowledge that it was not sampled in this study, thus restricting our sample universe and reinforcing confidence to our results. Now we state in the abstract and methods that "we use arboreal and leaf-litter lizard abundance data".

Some of the points raised by the reviewer on item 4 of his/her review also deserve additional efforts from the authors. As an example, mean width of the upper Madeira River was not mentioned anywhere, but this is an important issue when hypothesizing it could act as a riverine barrier. Also, information on sampling scheme are not yet clear enough. Visual searches were performed during the day only, or also during the night, since there are nocturnal species in the assemblage studied?

Response: We added the followed passages in order to attend to these pertinent suggestions: "...and its width varies from about 0,5 to 10 km depending on the river flow. The Madeira separates the Inambari and Rondônia endemism zones located along its left (west) and right (east) margins, respectively [20].". "The searching time in each plot varied between 40 and 60 minutes and was always conducted during the day.". We are not restricting the assemblage to diurnal lizards in the manuscript because species that are usually active during the night were also sampled in daytime hours.

The former reviewer also pointed that “authors suggest that the pattern found – the river acting as a barrier – was important. However, they mention that the lizard fauna found is widely distributed over the Amazon.” He then made some comments on this topic. The authors provided a pertinent response but I think the discussion on this topic could benefit from adding supporting literature on known distributions of some of the species treated therein, which are based in more historical datasets and which clearly show the pattern discussed.

Response: In Discussion, we clearly state that "We highlight that most of regionally isolated species in our sample are widely distributed throughout Amazonia outside our study area [13]...". In addition, in this revised version we acknowledge in the Abstract that "This finding suggests species have been historically isolated at one of the river banks, **or that distinct geomorphological features influence species occurrence at each river bank**.".

Minor comments Page 1

Line 3 - "Brasil Amazônico" does not make sense... Brazilian Amazonia would be more appropriated

Response: Replaced by "Brazilian Amazonia" throughout the text.

Page 2

Line 36 - you have used "river" in the title and "River" from now on. Check and standardize it throughout the text - River or river.

Response: Replaced by "Madeira River" throughout the manuscript.

Line 39 - delete space

Response: Space suppressed.

Line 44 - "along" would be better

Response: Replaced by "along" in three phrases.

Line 46 - "... over the extensive (nearly xxx km) study area." (I suggest you to briefly mention in the abstract the magnitude of the area to which these results aplly)

Response: Replaced by "over the extensive (nearly 500 km) study area. ".

Page 3

Line 35 - Brazilian Amazonia

Response: Replaced by "Brazilian Amazonia".

Line 72 - first cite frogs, then birds. Please also sort citations in ascending order, throughout the text.

Response: Replaced by "...explaining limited distribution of plants, frogs, birds, spiny rats, and primates". Citations were sorted in ascending order in this new version of the manuscript.

Line 72 - first cite spiny rats, then primates

Response: Replaced by "...explaining limited distribution of plants, frogs, birds, spiny rats, and primates".

Page 4

Line 82 - insert an Oxford comma before "and"; see additional remarks on it in line 182

Response: Comma added.

Line 82 - Ideally, you should cite them in an evolutionary rank. Then you will need to renumber references. Please also sort citations in ascending order.

Response: Now cited in evolutionary rank.

Line 84 - sort citations in ascending order

Response: Citations were sorted in ascending order in this new version of the manuscript.

Line 85 - sort citations in ascending order

Response: Citations were sorted in ascending order in this new version of the manuscript.

Page 5

Line 116 - basin (the "megadiverse region" you want to better understand is that one under the influence of the river, not the river itself).

Response: We added "basin".

Line 120 - delete space

Response: Space deleted.

Line 120 - insert space

Response: Space inserted.

Line 122 - As one of your hypothesis treats the upper Madeira as a barrier, please add some information on relevant characteristics of the river itself (e.g., river width).

Response: We added the following passage: "and its width varies from about 0.5 to 10 km depending on the river flow.".

Line 123 - Brazilian

Response: "Brazilian" inserted.

Line 123 - River (standardize)

Response: Standardized in uppercase letter.

Line 124 - River

Response: Changed to "River".

Line 124 - check standards of the journal (Fig instead of Fig.). Additionally, please add some information on the endemism zones (Rondônia and Inambari), so the mention to them can be properly evaluated when analyzing the elements in your Fig 1.

Response: Standardized to "Fig" instead of "Fig.". We added the following passage: "The Madeira separates the Inambari and Rondônia endemism zones located along its left (west) and right (east) margins, respectively [20].".

Line 126 - And what does it mean? These two endemism zones are only briefly mentioned in the Introduction and here in the legend for Figure 1. You should briefly discuss on the implications of this in your results.

Response: We added the following passage to Discussion: "In the upper Madeira River, assemblage divergence between river banks has been attributed to historical processes regionally reducing species dispersal [12], and delimiting the Amazonian endemism zones Inambari and Rondônia [20].".

Page 7

Line 153 - check and standardize throughout the text: you used either with or without hyphen

Response: Standardized as East and West Jirau (without hyphen). Jaci-Paraná has a hyphen because this is the original name of the locality.

Line 154 - see comment on line 153

Response: Standardized as East and West Jirau (without hyphen). Jaci-Paraná has a hyphen because this is the original name of the locality.

Line 103 – replace “e” by “and”

Response: Replaced by "and".

Line 106 - Brazilian Amazonia

Response: Replaced by "Brazilian Amazonia".

Lines 165-166 - Dates should follow a standardized sequence (e.g.,day-month- year, as you used here)

Response: Replaced by "We sampled each plot in four different periods (24 February to 26 April 2010, 30 July to 19 August 2010, 5 November to 26 November 2010, and 13 January to 4 February 2011)".

Line 172 – there is no fossorial species in your list.

Response: "fossorial" was excluded.

Line 174 - at what time of the day/night?

Response: Altered to "The searching time in each plot varied between 40 and 60 minutes and was always conducted during the day.".

Page 8

Line 178 – replace squamates by lizards '

Response: Changed to "lizard".

Line 181 – soil in lowercase

Response: "soil" now in lowercase.

Line 182 - Note: check journal style and consistently use (or do not use) the Oxford comma throughout all the text. In this paragraph, as an example, you alternatively used it (see line 180 - "soil texture, fertility, and flat-level") and did not use it (e.g., in lines 179 - "...foraging, resting and thermoregulation sites", and line 182 - "...Potassium, Zinc and exchangeable Aluminum").

Response: We opted to use the Oxford comma throughout the manuscript. The entire manuscript was revised with regard to the commas.

Line 184 – remove the dot before citation [61]

Response: Dot removed.

Page 9

Line 204 - along the same river bank (replace "within a" by "along the same")

Response: Changed to "...modules along the same river bank...".

Page 10

Line 224 - insert space between "predictors" and "that"

Response: space inserted.

Line 247 – bring to the suggested point the mention to Table 1, or, alternatively, to a point immediately after the mention of the three most abundant species. The information on the number of plots where each species was found do not appear in the table.

Response: We brought the mention of Table 1 to the point immediately after the three most abundant species.

Page 11

Line 251 - remove the mention to Table 1 here, and insert it at any point in the previous sentence. There is no information in Table 1 on the number of plots where each species was found.

Response: Mention removed.

Table 1 – check correspondence of values to each species: the values highlighted possibly correspond to *Alopoglossus angulatus*. Please relocate them to the correct line of the table.

Response: Values relocated to *Alopoglossus angulatus.*

Page 12

Table 1 – complete year of description of *Kentropyx calcarata* and of

Uranoscodon superciliosus

Response: Values completed: 1825 and 1758, respectively.

Line 265 - insert an Oxford comma after *bassleri*

Response: Comma added.

Line 267 - "and" not in italics. Please also add an Oxford comma before "and" .

Response: Replaced by "*Kentropyx calcarata,* and *Enyalius leechii...*".

Line 272 - replace "of" by "in" ; replace “and” by “or”

Response: Changed to "Plots ordinated according to their position in the upper Madeira River (west or east bank).".

Line 273 – add “bank” after “east”

Response: Changed to "Plots ordinated according to their position in the upper Madeira River (west or east bank).".

Page 13

Line 286 - insert an Oxford comma after “Búfalos”

Response: Oxford comma added.

Line 288 - standardize mention to the Akaike's criterion: "?AICc < 2", as used before.

Response: Standardized to "ΔAICc < 2".

Page 14

Table 2 - correct the word "margim". Check it in every Table. It appears more than once.

Response: Word corrected to "margin" throughout the table.

Line 292 – insert "to" before "the number"

Response: Corrected to "position relative to the number of trees...".

Page 15

Line 305 - standardize mention to the Akaike's criterion: "?AICc < 2", as used before.

Response: Standardized.

Table 3 - correct the word "margim". Check it in the table.

Response: corrected to "margin".

Page 16

Table 3 - correct the word "margim".

Response: Word corrected.

Lines 312-313 – Principal Coordinates Analysis (cite it according to the abbreviation PCA)

Response: Replaced by "Principal Coordinates Analysis".

Page 17

Line 331 - “East Jirau” without hyphen. Standardize it throughout the text.

Response: East Jirau was standardized without hyphen.

Line 332 - insert an Oxford comma between "sand, and clay"

Response: Comma inserted.

Line 333 - see comments on line 312.

Response: Changed to "Principal Coordinates Analysis".

Line 342 - ...[18], diurnal frogs [12], birds... (cite diurnal frogs before birds)

Response: "diurnal frogs" now cited before "birds".

Line 346 – “...with those obtained for frog assemblages”

Response: Replaced by "Our overall results are broadly consistent with those obtained for frog assemblages sampled in the same plots...".

Page 18

Lines 355-356 or elsewhere: did you ever discuss on the possibility of taxonomic bias contributing to this scenario? There are consistent and recent efforts to investigate the taxonomic status of frog species in the region, and such efforts are unparalleled regarding lizards. Maybe you can use recent literature to briefly discuss on that. Only 19% of the species in Table 1 were described in the 20th century, while 31% of the frogs considered in Dias-Terceiro et al. (2015) were described in 21th century and other three species were recognized as undescribed by the same authors.

Response: With regard to this pertinent comment, in the revised version we state that "The smaller proportion of regionally isolated lizard species (29.63%) is reasonably explained by the lower dispersal capacity of small and site-attached frogs compared with most lizards. A taxonomic bias may be also contributing to this scenario, since there are consistent and recent efforts to investigate the taxonomic status of frog species in the region [12], and such efforts are unparalleled regarding lizards.".

Line 360 - you could mention the distribution patterns for some of the species considered herein, depicted in Ribeiro Jr. (2015), as an additional source of evidence that the pattern you found is real, even though a few specimens were recorded during your study (the distribution shown for *E. leechii*, as an example, based in a large dataset).

Response: In Discussion, now we clearly state that "Even though the riverine-restricted geographic distribution of species such as *Kentropyx calcarata* observed in this study is supported by basin-level data, we highlight that most of regionally isolated species in our sample are widely distributed throughout Amazonia outside our study area [13].". In addition, in this revised version we acknowledge in the Abstract that "This finding suggests species have been historically isolated at one of the river banks, **or that**

distinct geomorphological features influence species occurrence at each river bank.".

Lines 362-363 - again, taxonomic bias may be also one of causes of such inconsistencies. What you are calling *Kentropyx altamazonica*, as an example, potentially represents a cryptic species, as other taxa in your Table 1. This is out of the scope of your work, but cannot be ignored

Response: In the revised version we wrote that "The smaller proportion of regionally isolated lizard species (29.63%) is reasonably explained by the lower dispersal capacity of small and site-attached frogs compared with most lizards. A taxonomic bias may be also contributing to this scenario, since there are consistent and recent efforts to investigate the taxonomic status of frog species in the region [12], and such efforts are unparalleled regarding lizards.".

Line 363 - sort citations in ascending order

Response: sorted in ascending order.

Page 19

Line 385 - you basically used "active visual search". Did you additionally use a systematic search using leaf litter plots? Or you merely searched leaf litter anurans instead, by "sweeping the leaf litter and removing debris"? Looking carefully at every microhabitat visually accessible and inspecting potencial retreats are common procedures during visual searches.

Response: Now we clearly state in Discussion: "However, we think that a possible sampling bias was mitigated by the large sampling effort associated to the combination of different **visual** sampling methods employed in this study.". We rephrased Methods for clarity and reached the followed wording: "We found lizards using active visual search, with two simultaneous observers positioned 10 m apart. In addition, we supplemented the sampling effort by sweeping the leaf litter and removing debris in a 2 m strip following the center line of the plot. This approach was particularly useful to increase the efficiency of sampling leaf-litter species (e.g. Alopoglossidae,

Gymnophthalmidae). Search on the vegetation and on the leaf litter was systematically and respectively conducted in the first and second half of each visual sampling in the plots, thus constituting two different and complementary methods. The searching time in each plot varied between 40 and 60 minutes and was always conducted during the day.".

Line 401 - cite in an evolutionary rank

Response: Cited in evolutionary rank: "frogs [12], snakes [79], and bats [80].". References were reordered in the list.

Line 403 – replace "within a" by "along a same"

Response: Replaced by "along a same riverbank".

Page 20

Line 410 - of which zoological group? is this a general trend for chordates? for reptiles? for tropical reptiles? your supporting references herein (58-60) are for snakes, including a temperate species. Can you replace or complement them with any citation on the effects of vegetation heterogeneity on tropical lizards?

Response: Changed to: "Heterogeneity in vegetation structure affects occurrence and abundance of tropical squamates due to variation in the availability of foraging, nesting, resting, and thermoregulating sites [58,60].". We changed "species" by "tropical squamates", and replaced the reference from a temperate environment [60] by a tropical one. We used references on snakes because we did not find examples related to lizards - probably due to the relative few studies on tropical lizards.

Line 413 - insert an Oxford comma after “humidity”

Response: Comma added after "humidity".

Page 21

Line 435 - insert an Oxford comma after “cover”

Response: Comma added after "cover".

Line 437 – “...snake [41], and bird [39] assemblages.”

Response: reordered in an evolutionary rank.

Line 447: insert “upper” before Madeira River

Response: changed to "both banks of the upper Madeira River...".

Page 22

Line 463 - sort citations in ascending order

Response: In the megadiverse Amazonian rainforests this has been achieved by a few studies [12,31,73].

Page 33

Line 725 - italicize *Liolaemus monticola*

Response: "*Liolaemus monticola*" italicized.

Page 34

Line 746 - not in italics

Response: Italics removed from "Boulenger".

Page 36

Line 803 - West Jirau (standardize)

Response: Standardized to "West Jirau".

Line 801 - check

Response: Changed to "Effect of environmental predictors on lizard assemblages composition".

Line 805 - check

Response: "Effect of environmental predictors on lizard assemblages composition".

Line 806 - East Jirau (standardize)

Response: Standardized to "East Jirau".

Line 806 – delete extra space before “Paraná”

Response: Extra space deleted.
