# Supplementary material for: Hierarchical effects of historical and environmental factors on lizard assemblages in the upper Madeira River, Brazilian Amazonia
Source: PLoS One. 2020 Jun 2;15(6):e0233881. doi: 10.1371/journal.pone.0233881 (PMC7266318; doi:10.1371/journal.pone.0233881)
Supplement: Attachment — Submitted filename: Response to Reviewers.docx [file pone.0233881.s004.docx]

**REBUTTAL LETTER**

PONE-D-19-15180
**Hierarchical effects of historical and environmental factors on lizard assemblages in the upper Madeira river, Amazonian Brazil**
PLOS ONE

**Responses to editor comments:**

*1. Please ensure that your manuscript meets PLOS ONE's style requirements, including those for file naming. The PLOS ONE style templates can be found at* <http://www.journals.plos.org/plosone/s/file?id=wjVg/PLOSOne_formatting_sample_main_body.pdf> *and* <http://www.journals.plos.org/plosone/s/file?id=ba62/PLOSOne_formatting_sample_title_authors_affiliations.pdf>

**Response:** All PloS ONE style standards have been revised.

2. *We note that Figure 1 in your submission contain [map/satellite] images which may be copyrighted. All PLOS content is published under the Creative Commons Attribution License (CC BY 4.0), which means that the manuscript, images, and Supporting Information files will be freely available online, and any third party is permitted to access, download, copy, distribute, and use these materials in any way, even commercially, with proper attribution. For these reasons, we cannot publish previously copyrighted maps or satellite images created using proprietary data, such as Google software (Google Maps, Street View, and Earth). For more information, see our copyright guidelines:* <http://journals.plos.org/plosone/s/licenses-and-copyright>*.*

*We require you to either (1) present written permission from the copyright holder to publish these figures specifically under the CC BY 4.0 license, or (2) remove the figures from your submission:*

**Response:** The satellite image used to compose Figure 1 was changed following the proposed guidelines and a new image was added in its place from The Gateway to Astronaut Photography of Earth (public domain).

**Response to Reviewers**

1. *My main concern is that all inference here is based on count and presence/absence data, which may be influenced by sampling errors. For instance, you may find 20 individuals of species A and 19 individuals of species B in a given plot, when in fact there was 120 individuals of species A and 20 individuals of species B. This is typically a false-negative type error. Alternatively, you may have misidentified species B, inducing false-positive errors in your counts. Similar problems occur when using presence/absence data when absence is not included and estimated in the model. This subject is well covered in the literature. You may want to have a look at Kéry and Schmidt 2008 (Community Ecology 9: 207-216), Fitzpatrick et al. 2009 (Ecological Applications 19: 1673-79), Miller et al. 2012 (Ecological Applications 22: 1665-74) and so on. Since sampling effort seems to be enough, I recommend applying a modeling technique that accounts for sampling errors (for instance, N-mixture models), which I find more appealing to discuss results arising from count data. However, I understand that you would have to make a major change in your manuscript, especially if results change, which I would not be surprised. At least, I recommend including a paragraph discussing this subject, since I find hard to believe that you have captured the exactly number of individuals and species present at a given site, and thus, the results may be biased.*

**Response:**

We agree with the reviewer that when we study ecological data based on abundance and presence / absence data, we may be masking sampling errors since every technique is biased and will depend on the probability of species detection. However, as in our study we conducted multiple data collections at every point, combined with more than one sampling method, we think that we minimized sampling bias as much as possible.

As for the suggested modeling technique (N-mixture models): we opted for the second option suggested by the reviewer, which would be to continue using the models already proposed and not change our analyzes. We add a paragraph discussing these type I and II errors and the possible biased results generated from the sampling and that could be altering our results in some way. We justify our decision not to use N-mixture models because the same consist of two linked generalized linear models, however, for the realization of this model, the ordering method chosen by us (principal coordinate analysis - PCoA) cannot be used since it does not support the input of negative values. Given that the PCoA returns negative values of scores on its axes we could not be using this sorting method. Therefore, we chose to continue with PcoA to the detriment of the other sorting methods that could be substituted to meet the assumption of generalized linear models, since our choices followed the one suggested by GOTELLI & ELLISON (2011, page 446 of the book Principles of Statistics in Ecology). These authors recommend the use of PcoA for “sorting applications in which the objective is to preserve the original multivariate distances between observations in the reduced space (sorting)”, especially when we intend to use the axes generated in the ordering in later analyzes. With regard to the use of other methods such as the widely used NMDS, the axes are not orthogonal, that is, we could not choose just one of the axes as a predictor of our species composition.

We carried out several previous analyzes with other sorting methods, different types of returns, and simulations including the removal of species that presented low abundance. In each simulation we had the precaution to show to other researchers in the area and request their opinions before deciding on the methods presented in this version of our manuscript. Among these simulations, we carried out the NMDS which captured 46.64% of the variation in species composition with two axes and demonstrated that the lizard assemblages differed significantly in relation to the banks of the Madeira River (MANOVA: Pillai trace = 0,205, F_1,80_= 10,229, p <0,001). As for the results of the regressions with the environmental variables, the ordering of the NMDS plots for the left side of the river was only possible using three NMDS axes due to the little variation in the data with two axes (which led us to prefer the analysis main coordinates - PcoA), and as a result captured 70% of the variation in species composition. The explanation for the right margin using two NMDS axes was 57%. As for the possible structuring environmental variables, the effect of the vegetation structure (number of trees) on the left side (F_7,38_=2,567, p=0,04), and for the right margin, only elevation was identified as a factor influencing the composition of the lizard assemblages (F_1,70_=7,737, p<0,001). With all the simulations with our data we can see a strong effect of the modules themselves and the need to analyze the modules Ilha da Pedra (west bank) and Jirau-D (east bank) separately from the rest of the sample units, so we arrive at our current results .

1. *Your sample includes temporal variation (extended for a year, or so) but there are no temporal covariates among your predictors. Lizard activity is usually related to temperature, sunlight, rainfall and so on. You probably sampled plots in different days and different hours within a day, and ciclicity occurs even within individuals. How do you think the absence of temporal predictors affect your results? For instance, Figure 2 shows species ‘abundances on each river bank. Some species seems to be common on one bank, but not the other. Is this really the barrier effect of the river? By ignoring temporal variability, you assume sampling did not vary along the year.*

**Response:**

Each sampling campaign was carried out at a different time of the year: Campaign I- 24 February to 26 April 2010; Campaign II- July 30 to August 19 2010; Campaign III- November 5 to 26 2010; and Campaign IV- January 13 to February 4 2011. In each of these campaigns, all plots inserted in the different modules were sampled, that is, each plot was sampled in four different times of the year. Therefore, we think we minimized the sampling bias regarding the presence / absence of species in the plots. In fact, the activity of the lizards is closely related to variations in temperature, light and precipitation in a given environment. With this in mind, the plots were sampled at different times. We believe that our sampling method that combined active visual search, with two simultaneous observers, supplemented by sweeping the leaf-litter, minimized the lack of temporal predictors in the presence/absence results.

2. *Authors suggest that the pattern found – the river acting as a barrier – was important. However, they mention that the lizard fauna found is widely distributed over the Amazon. This is somewhat confusing to me because if species were well-distributed, they would probably be found everywhere. To explain that, authors mention that the barrier effect of the river may vary, suggesting seasonality. This makes me wonder if species are truly isolated, as suggested. The barrier effect could be only temporarily and the results found are explained by the time frame of the study. I would like authors to clarify that point.*

**Response:**

Actually, most of the lizard species found in our study are widely distributed across Amazonia, and when we say that these species are isolated, we are not trying to extrapolate our results to the entire length of the river, so we were careful to keep that registered in the discussion (from line 385). as we do not have information for other locations along the length of the Madeira River.

We did not want to suggest seasonality when we wrote that the river barrier effect may vary. The different Amazonian rivers (and even different parts of a same large river) have distinct widhts, current speeds, water types and geological dynamics, which may affect their role as an effective geological barrier.

Even widely distributed species in Amazonia are not necessarily found everywhere, since the configuration of the Amazon Basin can be related to a mosaic of different phyto-regions, observed both on a local and regional scale, with different geological ages and origins between the different fractions of the basin, a fact that leads to historical evolutionary differences between areas and that become determinant factors for the differences in lizard species richness in the region. Even within each area of endemism, with no current geographical barriers, ecological differentiation can occur (Ferrão *et al*., 2017; Ortiz et al. 2018). The isolation of lizard species in one of the banks of the river may be related to geomorphological heterogeneity along the course of the Madeira River, and even at different sampling dates, we still observe the restricted presence of some species to certain banks of the river.

Ferrão, M.; Moravec, J.; Fraga, R.; Almeida, A.P.; Kaefer, I.L.; Lima, A.P. 2017. A new species of Scinax from the Purus–Madeira interfluve, Brazilian Amazonia (Anura, Hylidae). *ZooKeys*, 706: 137–162.

Ortiz DA, Lima AP, Werneck FP (2018) Environmental transition zone and rivers shape intraspecific population structure and genetic diversity of an Amazonian rain forest tree frog. Evolutionary Ecology 32(4):359-378

3. I *think your manuscript would benefit from contrasting your results with other lizard systems around the world. For instance, are there examples of rivers acing as barriers for lizards elsewhere? What about other reptiles? What are the covariates related to isolation for lizards or reptiles in general? In addition, you could provide some information about the Amazon basin. For instance, what is the mean width of the big rivers? Finally, concerning the species found. What is the habit of the most abundant species? Did you find terrestrial, arboreal and fossorial species equally? Does you results relate to species ‘habits? I believe that finding terrestrial species (the teiids, for instance) may be easier than arboreal (Enyalius?) or fossorial. In the same way, diurnal species will be easier to find during the day and the opposite for nocturnal. How was the sampling scheme? Can you really talk about lizards in general of a guild?*

**Response:**

We thank the reviewer and agree that some information needs to be added, so we have included in the discussion other examples of lizards in the Amazon and around the world, and data on the covariates associated with restriction of geographic distributions. Information on the width of the largest Amazonian rivers was added to the methodology when mentioning the Madeira River, as it is one of the largest and most important rivers in the Amazon basin.

Regarding the species habits, we found the tree species *Gonatodes humeralis*, and *Norops fuscuoratus* as the most abundant, followed by the teiid *Ameiva ameiva*. The fossorial or semi-fossorial species were the ones with the lowest abundances, a fact already expected and observed in different studies on lizard assemblages. We think that the results are related to the habits of the species, as included in the discussion (starting from line 428), where we associate the results of the gradient number of trees to some tree species found.

Regarding the species activity time (more detail in the following answers to the reviewer below), most species are diurnal or crepuscular and few are nocturnal, but easily detectable to experienced researchers in the region.

 	We observed that our sample is within the richness already highlighted in the local and regional literature for Amazonia, and we think we gave a good representation of how the lizard community in that region is structured.

With regard to the term guild, we decided not to apply because the observed species use different and complementary resources, thus not constituting a specific guild with regard to diet or habitat use, for example.

**Minor comments**

- L62: substitute "design" by "designed"

**Response:** Replaced in text

- L84: change "the latter" by "lizards"

**Response:** Replaced in text

- L161: I am not sure I would exclude those sites since you may overestimate predictor effects.

**Response:** We decided to maintain the exclusion, as some of these sites coincided with areas of difficult access, for example, due to fallen trunks, making it difficult to travel along the plot, or even partially flooded, and could contribute to the low probability of detection. Thus, we decided to exclude all sites that contained zero individuals.

- L166-167: you have four surveys on each plot. I suppose there was variation on specie´s counts over surveys, due to several aspects, including seasonality (which you ignored). I advise on modeling this variability on counts.

**Response:** Question previously answered.

- L168-169: what time did you search? Are all lizards diurnal/nocturnal? Any potential bias here?

**Response:** Most of Amazonian lizards are diurnal. Even those that start their activity at dusk, such as *Uranoscodon superciliosus* (Linnaeus, 758), are easy to detect because they usually rest over tree branches while inactive. Regarding campaign schedules, all were carried out during the day. However, the first and last were carried out in the afternoon and early evening, and the second and third were carried out in the morning and early afternoon. Thus, we contemplated different activity periods, maximizing detectability.

- L177: I would rather call these ‘environmental predictors’, since gradients may remind other things as well.

**Response:** Replaced in text.

- L183: change ‘.’ by ‘,’

**Response:**	Replaced in text.

- L221-223: maybe it is just me, but the way it is written seems that you avoided correlated models, not predictors.

**Response:** The sentence was rewritten for clarity.

- L223-226: what was the cut-off point?

**Response:** From r = 0.51. We added this information in the text.

- L233: what do you mean by “corrected for few parameters”?

**Response:** We wanted to say that we did use Akaike´s Information Criterion (AIC) for model selection, but the one corrected for small samples: the AICc. The sentence was poorly written in the first version and rephrased for clarity.

- L234-235: what about the non-nested models? Why using the cut-off point if important model weight may be left out? Isn´t model averaging a better strategy depending on the case?

**Response:** I am not sure if I understood the question, and in what our analysis differs from what has already been done in the literature. We stipulated the ∆AICc <2 cut, due to the number of possible models with different combinations between the variables, and this cut would inevitably exclude some models at the expense of those more parsimonious models.

- L261-262: how can you be sure those species were not there?
  Table 2. what kind of models did you build? Only those with main effects, excluding additive and interactive effects? Did you try combining all best predictors in one model?
  Figure 3. which model did you use to plot the relationship between abundance and number of trees? The top model or all three models? Notice that model weights are similar and all of them include the number of trees.
  Table 3: This table is somewhat hard to read. Please, insert a backspace between West and East or a line between them. In addition, maybe you could reduce the names of the predictors or show their first letters only.

**Response:** Yes, several models were built, with different combinations between different predictors that did not correlate with each other. In relation to figure 3, we show a direct ordination of the values from the gradient number of trees and the relative abundance of species by plots. With regard to the table, we added additional lines as suggested by the reviewer for a better visualization.

- L293: again, I would rather call ‘predictor’ or ‘covariate’ instead of ‘gradient’.

**Response:** Replaced in text.

- L294: which model? Not the top model nor the third best-ranked model, if I read the table correctly (please, revise the table configuration). However, all models present similar AIC weights. How was the rationale behind using the only model that presented a significant effect? Did you try model averaging? I would not pick just one model among several to present a result, especially if they diverge.
  Figure 4. Same comment as in Figure 3.

**Response:** We decided to graphically show only the significant models (P < 0.05). We could really include the images of all the models (which were marginally significant), but we wanted to be synthetic and illustrate these results with a single image due to the length of the article.

- L352: there is a typo in this line.

**Response:** Corrected in text.

- L389-390: I am not convinced about that without including temporal predictors in the analysis. Was it too hot or too cold during some of the samplings? Were there thunderstorms or heavy rainfall in, at least, some of the sampling occasions?

**Response:** Samples were not taken in the rain. When this happened the samplings were postponed.

- L400-405: I find the discussion on conservation implications interesting, but I am not sure you provided all the elements for that. For instance, what kind of forest did you measure, native on invasive? Were there pine plantations (which are also categorized as forests)? How is the relationship between lizard assemblage and forests in general?

**Response:** All sampling modules were installed in original, preserved Amazonian *terra-firme* forests that are not influenced by other types of vegetation.

- L419: you start this paragraph stating that species turnover was influenced by the distance from the river. But this was not found in all samples, as you stated in the same paragraph. Given that your samples covered only 5 km from the river, which in the Amazon, does not seem to be a huge area, what distance do you think you would find such effect?

**Response:** We believe that species turnover is related to several aspects in addition to the distance from the river, and that stipulating a margin distance value for the Amazon is a risk, since the region has a large environmental heterogeneity between the interfluvial regions, and even over small distances of 5km we could have significant results in the turnover of species, especially those less vagile. However, many gaps need to be filled for Amazonian lizards, despite recent efforts to answer such a question.

- L427-428: what do you mean by "...was returned..."?

**Response:** We used returned in the sense that it was observed, but the word was replaced in the text.

- L451-452: what do you mean by "efficient methods for multi-scale sampling"? Can you provide suggestions about what you would say is an efficient method?

**Response:** Methods that present a spatial standardization, easily replicable for different species (modular), and preferably that can be carried out for a long time. An example of a good method is the one used here in our study, the RAPELD- Rapid Assessments and Long-term Ecological Research (Magnusson et al., 2005). These modules are part of a network of permanent standardized transects installed in the Amazon by the Biodiversity Research Program (PPBio) of the Brazilian Science, Technology, Innovations and Communications Ministry (Magnusson et al., 2013). An example of sample desing is shown in the figure below. In this method the plots follow the terrain level curve, minimizing possible topographic (environmental) effects within the plot (sampling unit). RAPELD has already been used in several localities around the world <https://ppbio.inpa.gov.br/sites/default/files/Biodiversidade_e_monitoramento_ambiental_integrado.pdf>. cited 10 March 2019.

The lizards along the road BR-319 in the Purus-Madeira interfluve, Brazilian Amazonia (Squamata, Lacertilia). Herpetology Notes 12: 689-697.
